# Aggregation‐Induced Phosphorescence of a *Trans*‐Bis(iminomethylpyrolato)Platinum Complex Bearing a Polymethylene Vaulted Structure: Chain Length–Dependent Solid‐State Emissions

**DOI:** 10.1002/chem.202501670

**Published:** 2025-07-31

**Authors:** Soichiro Kawamorita, Shufang Huang, Atsushi Yoshida, Shuichi Suzuki, Takeshi Naota

**Affiliations:** ^1^ Department of Chemistry Graduate School of Engineering Science The University of Osaka Machikaneyama Toyonaka Osaka 560–8531 Japan

**Keywords:** aggregation‐induced emission, minimum energy crossing point, phosphorescence, platinum

## Abstract

Aggregation‐induced emission (AIE) has recently attracted considerable attention. Although phosphorescent transition‐metal complexes exhibit significant potential as aggregation‐induced emission luminogens (AIEgens), their exploration and utilization have remained limited, partly due to the insufficient understanding of the underlying mechanisms. Here, we report a new series of platinum(II) complexes, *trans*‐bis(2‐iminomethylpyrrolato)platinum derivatives (**1a**–**d**), which feature vaulted polymethylene chains of varying lengths (n = 9–12) that bridge over the metal atom. Notably, racemic complexes **1b** and **1d** (with chain lengths of 10 and 12) exhibit strong yellow emission in the crystalline state (quantum yields of 24% and 33%, respectively), despite showing no emission in solution. X‐ray diffraction analysis revealed face‐to‐face dimer packing in these emissive crystals, suggesting that dimer formation contributes to their enhanced emission. In contrast, complexes with odd‐numbered chain lengths (**1a** and **1c**) showed no such emission enhancement. Density functional theory (DFT) calculations revealed that the minimum energy crossing point (MECP) between the excited triplet and ground state lies at significantly higher energy in the dimeric structure than in the monomer, suggesting suppression of nonradiative decay in the crystalline state. This work provides a design strategy for inducing solid‐state phosphorescence by controlling the 3D molecular structure to facilitate favorable molecular stacking while suppressing nonradiative decay pathways.

## Graphical abstract

Platinum(II) complexes featuring a vaulted polymethylene bridge exhibit efficient AIE. An odd‐even effect in chain length is also observed in the AIE behavior, where strong phosphorescence emerges from dimer formation in crystals. Theoretical calculations imply that dimerization raises the MECP, suppressing nonradiative decay.

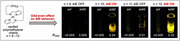

## 1 Introduction

Luminescent molecules that change their photophysical properties in response to external stimuli have emerged as versatile materials for imaging and sensing applications in molecular biology,^[^ ^1^ ^]^ polymer science,^[^ ^2^ ^]^ supramolecular chemistry,^[^ ^3^ ^]^ and flow mechanics.^[^ ^4^ ^]^ The aggregation‐induced emission luminogens (AIEgens) are representative materials that drastically change their luminescent properties in response to external environmental stimuli, exhibiting enhanced luminescence in the solid state despite showing no or weak luminescence in the solution state.^[^ ^5^ ^]^ Conventional molecules exhibit aggregation‐caused quenching (ACQ) due to molecular stacking,^[^ ^6^ ^]^ while the AIEgens have a molecular design where deactivation of the excited state is effectively suppressed in the aggregated state.

The mechanism of aggregation‐induced emission (AIE) was initially regarded as due to the nonradiative deactivation caused by internal conversion, such as the intramolecular rotation or vibration of substituents in the solution phase, and the restriction of the intramolecular motion (RIM) in the solid state leading to high emission efficiency.^[^ ^5b^ ^]^ Based on this mechanism, extensive research into molecular design incorporating multiple rotating substituents, such as phenyl groups. More recently, it has been suggested that this explanation does not fully account for certain deactivation mechanisms. The restricted access to conical intersections (RACI) mechanism has been proposed, in which deactivation via crossing points between the excited state and the ground state, reached through molecular distortion, is suppressed in the solid state.^[^ ^7^ ^]^

Some d‐block transition metal complexes, such as those containing Ir(III), Pt(II), Au(I), and Re(II) atoms, exhibit phosphorescence due to their strong spin‐orbit coupling, making them widely applicable in various sensing and imaging materials,^[^ ^8^, ^9^ ^]^ with particular significance in the development of organic light‐emitting diodes (OLEDs).^[^ ^10^ ^]^ However, even in such phosphorescent metal complexes, ACQ is known to result in phosphorescence quenching in aggregated states.^[^ ^11^ ^]^ Therefore, the development of phosphorescent metal complexes with AIE properties and the elucidation of their mechanisms are crucial for molecular design and various applications of phosphorescent AIEgens.

In particular, phosphorescent Au(I) and Pt(II) complexes possessing linear and planar coordination structures tend to induce molecular stacking and lead to ACQ. On the other hand, these complexes have exposed d orbitals, which facilitate metal‐metal interactions and can alter or exchange the emission mechanism itself in the solid state.^[^ ^12^ ^]^ Therefore, in these complexes, 3D molecular design significantly influences AIE properties. However, unlike the extensively studied AIE mechanisms in organic molecules, those in metal complexes remain underdeveloped.^[^ ^13^, ^14^ ^]^

**Scheme 1:**
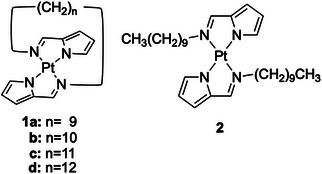
Molecular structures of vaulted and nonvaulted complexes **1** and **2**.

As part of our program to develop phosphorescent metal complexes with tailored 3D structures,^[^ ^15^ ^]^ we have found that *trans*‐bis(iminomethylphenoxy)platinum, which has a vaulted structure with polymethylene or PEG chains bridging over the metal atom, shows efficient AIE properties.^[^ ^15a,d,e,h,k,m^ ^]^ This molecular design prevents the nonemissive continuous stacking of planar platinum complexes and promotes suitable aggregation structures for emission; however, the detailed mechanism behind this AIE phenomenon remains unclear. In this study, we discovered a unique AIE phenomenon in *trans*‐bis(iminomethylpyrolato)platinum complexes **1** having a vaulted polymethylene chain, as illustrated in Scheme 1. These complexes exhibit polymorphism, with strong emission observed in crystals formed under certain recrystallization conditions, whereas other crystalline forms remain nonemissive. In addition, no emission is observed in solution. Interestingly, the polymethylene chain length affects the AIE phenomena: while no emission was observed with chain lengths of n = 9 (**1a**) and 11 (**1c**), strong emission was observed with chain lengths of n = 10 (**1b**) and 12 (**1d**) in the crystalline state. This odd‐even effect in emission behavior suggests that subtle changes in molecular geometry and packing induced by the chain length critically influence the excited‐state deactivation process. These molecules are particularly suited as a model AIEgen for investigating excited‐state deactivation mechanisms, as they can form both highly emissive and nonemissive crystalline states depending on the conditions. Therefore, this study also aims to elucidate the AIE characteristics of this molecule through theoretical calculations, focusing on excited‐state and minimum energy crossing point (MECP)^[^ ^15^, ^16^ ^]^ between S_0_ and T_1_ to evaluate the ease of nonradiative deactivation.

## 2 Results and Discussion

**Figure 1:**
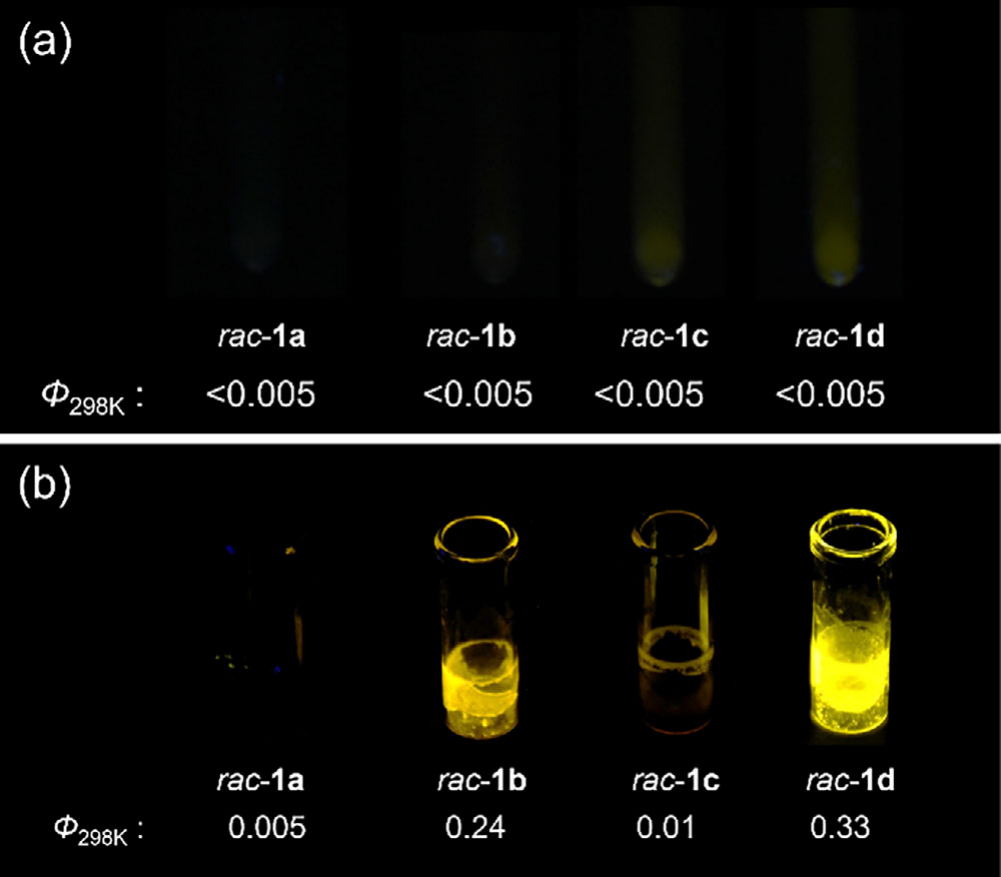
Photographs of complexes *rac*‐**1a**–**d** under UV irradiation (365 nm) in a) solution and b) crystalline state obtained from acetonitrile at 5 °C, along with their quantum efficiencies.

The solution of the vaulted complexes **1a**–**d** (racemic mixture, 2‐MeTHF, 1.0 × 10^−4^ M) shows no emission (Figure 1a), while the strongly yellow‐emissive crystals were obtained for *rac*‐**1b** (*Φ*_298K_ = 0.24) and *rac*‐**1d** (*Φ*_298K_ = 0.33) with polymethylene chain lengths of 10 and 12 through the natural evaporation from acetonitrile solution at 5 °C (Figure 1b and Table 1), exhibiting remarkable AIE phenomena. No emission was observed in the crystalline state for *rac*‐**1a** and *rac*‐**1c**, which have polymethylene chain lengths of 9 and 11, respectively (Figure 1b). This indicates that the emergence of the AIE phenomenon is dependent on the length of the bridging polymethylene chain. The complex **2**, which lacks a vaulted structure and has linear alkyl chains, exhibits nonemissive behavior both in solution and in the solid state (Table S1, S2), indicating that the molecular framework itself does not possess luminescent properties at room temperature.

**Table 1:** Photophysical data for **1** and **2**.

Entry	Complex	State	Recryst. Temp.	λ_max_^[^ ^a^ ^]^ [nm]	*Φ*_298K_^[^ ^[a]^, ^[Link]^ ^]^
1	*rac*‐**1a**	Crystal^[^ ^c^ ^]^	5 °C	569	0.005
2	*rac*‐**1b**	Crystal	5 °C	564	0.24
3	*rac*‐**1c**	Crystal	5 °C	558	0.01
4	*rac*‐**1d**	Crystal	5 °C	557	0.33
5	(*R*)‐**1d**	Crystal	5 °C	537	<0.005
6	(*S*)‐**1d**	Crystal	5 °C	537	<0.005
7	*rac*‐**1d**	2‐MeTHF Solution^[^ ^d^ ^]^	‐	542	<0.005
9	*rac*‐**1d**	Crystal	25 °C	539	0.02
10	*rac*‐**1d**	Crystal	60 °C	539	<0.005
11	**2**	2‐MeTHF Solution^[^ ^d^ ^]^	‐	547	<0.005
12	**2**	Crystal	5 °C	549	<0.005
13	**2**	Crystal	25 °C	546	<0.005
14	**2**	Crystal	60 °C	547	<0.005

^[a]^ *λ*_ex_ = 420 nm for **1** and 430 for **2**.

^[b]^Determined by the absolute method using an integrating sphere.

^[c]^The crystal was obtained from acetonitrile.

^[d]^1.0 × 10^−4^ M.

**Figure 2:**
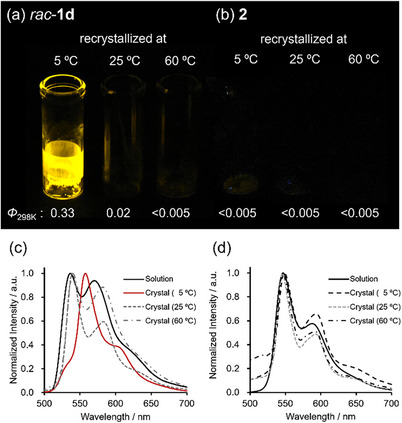
Photographs of crystals of complexes a) *rac*‐**1d** and b) **2** obtained at 5, 25, and 60 °C from acetonitrile under UV irradiation (365 nm). Emission spectra of c) *rac*‐**1d** and d) **2** in 2‐MeTHF solution (1.0 × 10^−4^ M) and their crystalline states obtained at the respective temperatures (*λ*_ex_ = 420 nm for *rac*‐**1d**, and 430 nm for **2**). The shoulder observed around 520 nm in (d) originates from residual excitation light due to the very weak emission of **2**.

The AIE properties exhibited by compounds **1b** and **1d** are dependent on the recrystallization conditions, which were investigated as described below. Emission properties of *rac*‐**1d** and nonvaulted **2** were evaluated in crystalline states obtained by varying recrystallization temperatures (Figure 2; see also Figures S17–S26 for complementary photophysical properties). The crystalline states were obtained by natural evaporation of acetonitrile solutions at three different temperatures: 5 °C, 25 °C, and 60 °C. The crystals obtained at 5 °C exhibited strong emission, as shown in Figure 2a. Notably, in contrast to typical AIE‐active compounds, the addition of water as a poor solvent to THF solutions of *rac*‐**1d** did not lead to enhanced emission but instead resulted in nonemissive precipitation (Figure S27). Meanwhile, those obtained at 25 °C and 60 °C displayed weak or no emission, with *Φ*_298K_ values of 0.02 and < 0.005, respectively. In contrast, complex 2 remained nonemissive under all recrystallization conditions, as clearly shown by the photographs in Figure 2b. The emission spectra of *rac*‐**1d** and **2** in solution, as well as those in the crystalline states obtained at different recrystallization temperatures, are shown in Figure 2c,d. The emission maximum of *rac*‐**1d** in solution was 542 nm. Crystals obtained at 25 °C and 60 °C exhibited *λ*_max_ values of 539 nm, whereas the strongly emissive crystals obtained at 5 °C showed a red‐shifted *λ*_max_ of 557 nm (Figure 2c, Table 1). For complex **2**, both in solution and in each crystalline state, the emission maxima were observed around 547 nm, with little change in the emission maxima across these states (Figure 2d). TDDFT calculations (Figures S28–S31 and Table S1 and S2) suggest that these emission bands arise from a mixture of intraligand charge transfer (ILCT) and metal‐to‐ligand charge transfer (MLCT) states. In the case of the dimeric crystalline form obtained at 5 °C, the observed red‐shift is consistent with stabilization of the ILCT/MLCT excited state via intermolecular interactions within the dimer packing structure.

The emission lifetime of the emissive *rac*‐**1d** crystal obtained at 5 °C was 2.55 *µs*, with radiative decay constant (*k*_r_) and nonradiative decay constant (*k*_nr_) of 13 × 10^4^ s^−1^ and 26.3 × 10^4^ s^−1^, respectively (Figures S44, S45, Table S2). The weak emissive crystals prepared at 25 °C had a lifetime of 0.07 µs, with *k*_r_ of 29.4 and *k*_nr_ of 1440 s^−1^, indicating that the significant increase in *k*_nr_ compared to the emissive crystal.

The complexes **1** have planar chirality due to the vaulted structure with alkyl chains, and interestingly, the crystals from optically pure compounds show only nonemissive or weakly emissive properties, even in the case of **1b** and **1d** (Figure S32). These results suggest that the strong emission in the crystalline state is governed by the length of the alkyl vaulted chains, recrystallization conditions, and the chirality of the molecules.

**Figure 3:**
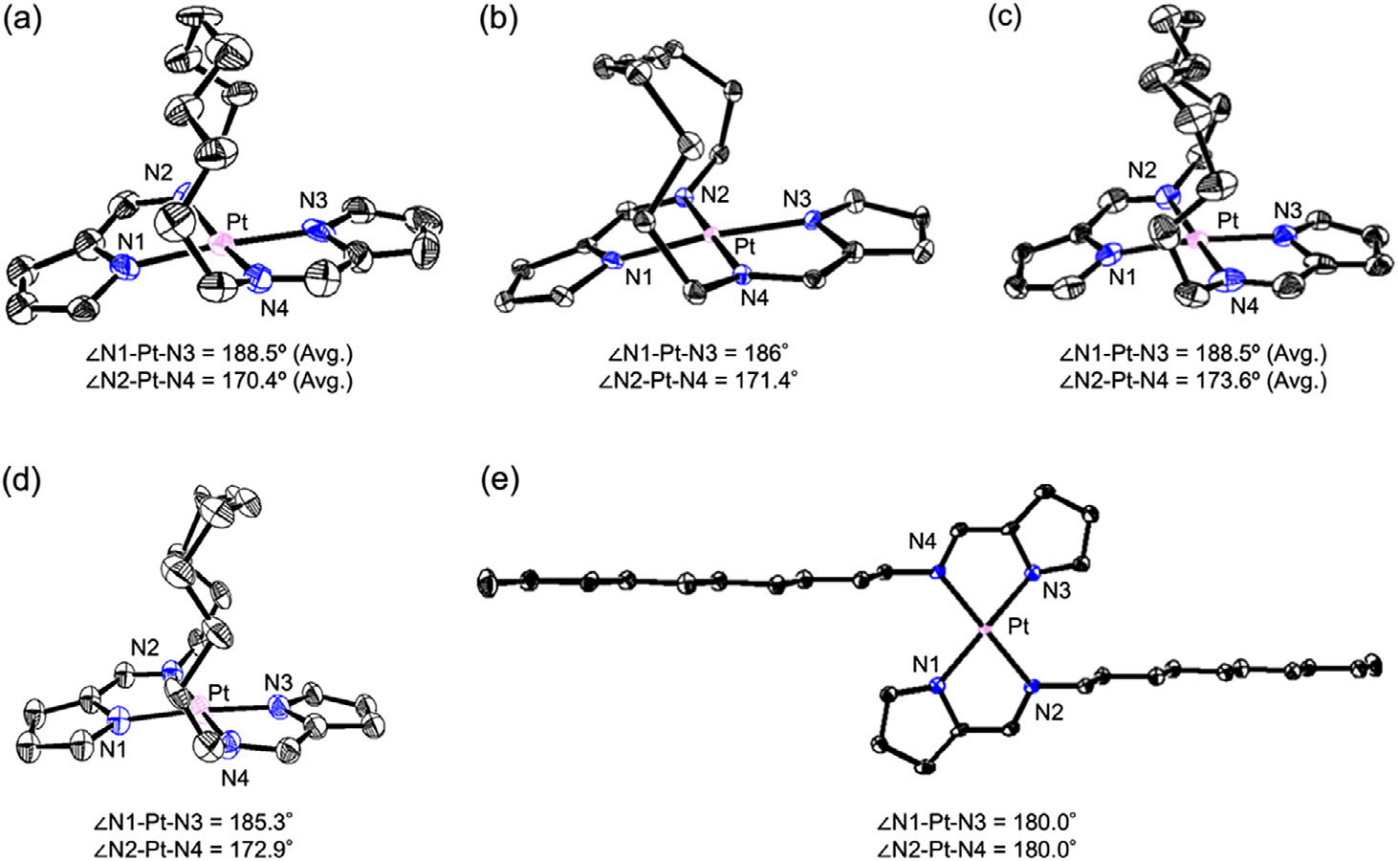
ORTEP representations and angles of N1‐Pt‐N3 and N2‐Pt‐N4 of (a) *rac*‐**1a**, (b) *rac*‐**1b**, (c) *rac*‐**1c**, (d) *rac*‐**1d** and (e) **2**. Thermal ellipsoids are shown at the 50% probability level.

**Figure 4:**
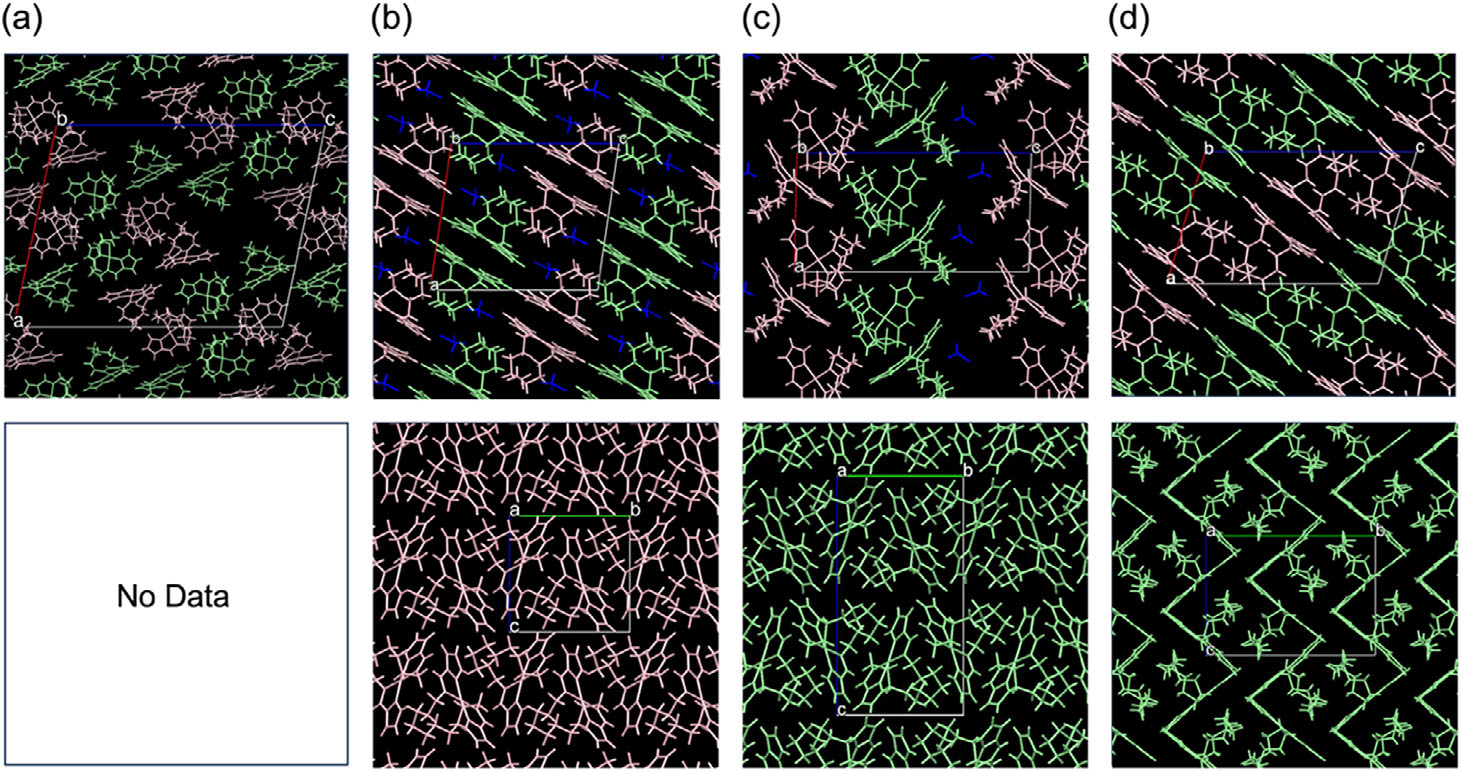
Packing structures obtained from single‐crystal X‐ray analysis for compounds a) **1a**, b) **1b**, c) **1c**, and d) **1d**. The upper row represents the racemic mixture, while the lower row corresponds to the enantiomerically. The pink and green molecules represent the *S*‐ and *R*‐enantiomer, respectively. The packing of a racemic mixture of **1b** and **1c** includes acetonitrile as the crystallization solvent.

The molecular structure (Figure 3) and packing structure in the crystalline state (Figure 4 and Tables S5–S7) of the complexes **1a**–**d** and **2** were elucidated through X‐ray diffraction (XRD) analysis of the single crystals obtained from acetonitrile solution at 5 °C.^[^ ^17^ ^]^ The molecular structures presented in Figure 3 correspond to the racemic forms of **1a**–**d**, and reveal a characteristic vaulted geometry. In the molecular structure of the complex **1a,** the vaulted alkyl chain lifts the imine nitrogens N2 and N4 above the platinum atom, resulting in an N2‐Pt‐N4 angle of 170.4° (averaged value of multiple molecular units). In contrast, the pyrrole ring nitrogen N1 and N3 are lowered below the platinum atom, giving an N1‐Pt‐N3 angle of 188.5° (averaged value) (Figure 3a). This characteristic structural distortion is consistently observed across the vaulted complexes **1a**–**d**. Specifically, the N2–Pt–N4 angles for **1b**, **1c**, and **1d** are 171.4°, 173.6°, and 172.9°, respectively, while the corresponding N1–Pt–N3 angles are 186.0°, 188.5°, and 185.3° (Figure 3b–3d). In the complex **2**, the four nitrogen atoms and the platinum atom are placed in the same plane, with N1‐Pt‐N3 and N2‐Pt‐N4 angles both at 180.0° (Figure 3e).

Recrystallization was performed for both racemic and enantiomerically pure forms of the vaulted complexes, yielding a total of seven distinct crystal structures (Figure 4). Suitable single crystals of the enantiopure form of **1a**, however, could not be obtained despite repeated attempts. The emissive crystals of the racemic **1b** and **1d** have dimer structures, where the two homochiral platinum coordination planes interact face‐to‐face (Figure 4b upper and 4d upper). The Pt···Pt distance is 3.30 Å for **1b** and 3.38 Å for **1d**, both of which are shorter than the sum of the van der Waals radii of platinum (3.5 Å), indicating the presence of Pt‐Pt interactions. The π‐π stacking interactions between two platinum coordination planes are also observed. This face‐to‐face dimer structure can also reasonably explain the red shift in the emission spectrum (Figure 2c). In contrast, the nonemissive crystals do not form dimers but instead stack with C─H···π interactions between the alkyl chains and the aromatic rings (Figure 4a,b bottom, 4c and 4d bottom; see also Figure S33 for the crystal structures obtained at 25 and 60 °C). These findings clearly demonstrate that the face‐to‐face dimer structure plays a governing role in enabling strong emission, highlighting the critical importance of solid‐state packing in dictating the photophysical properties of these complexes. The crystal packing of nonvaulted **2** has continuous π‐π and C─H···π interactions (Figure S34), which reasonably explains its nonemissive nature. These findings clearly demonstrate that the face‐to‐face dimer structure plays a governing role in enabling strong emission, highlighting the critical importance of solid‐state packing in dictating the photophysical properties of these complexes.

**Figure 5:**
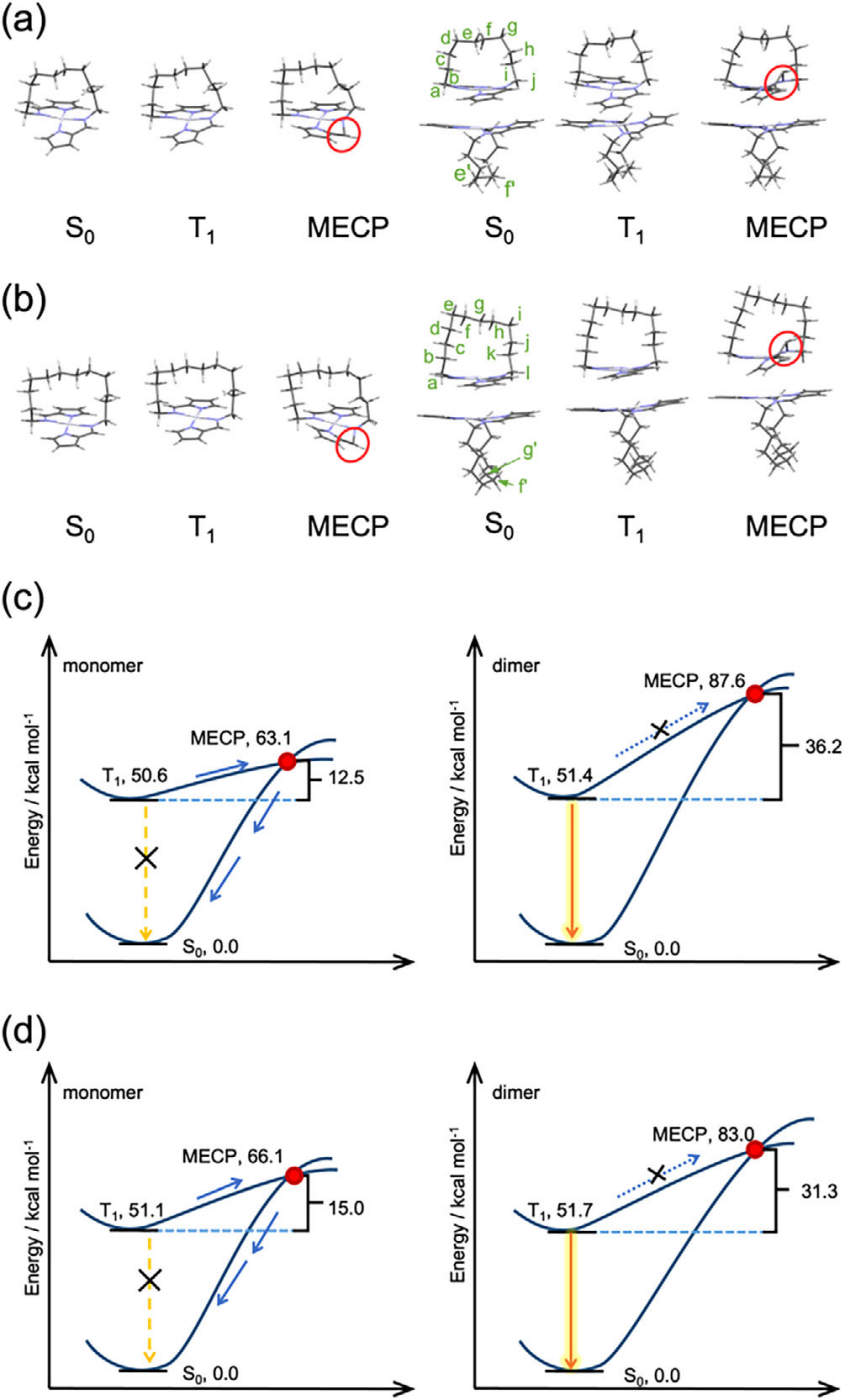
DFT‐calculated molecular structures and energy diagrams of S_0_, T_1,_ and MECP states for **1b** and **1d**, with monomers shown on the left and dimers shown on the right. The calculated molecular structures are shown for a) **1b** and b) **1d**. The corresponding energy diagrams for the S_0_, T_1_, and MECP states are shown for c) **1b** and d) **1d**.

To gain insights into how the dimer structures in the emissive crystals contribute to their emissive nature, DFT calculations were performed. The ground state (S_0_), triplet excited state (T_1_), and MECP between S_0_ and T_1_ were calculated for both the monomer and dimeric structures of **1b** and **1d**. The molecular structures obtained from crystal packing were used for geometry optimizations. For the calculation of the dimer structures, the Pt‐Pt distances and the torsion angles between the two topmost carbon atoms in the alkyl chains (torsion angles Ce‐Cf‐Cf’‐Ce’ for **1b** and Cf‐Cg‐Cg’‐Cf’ for **1d**, as shown in Figure 5) were constrained to reproduce the compressed environment of the crystalline state. The CAM‐B3LYP functional and the def2‐TZVP basis set were employed for all calculations, and the RIJCOSX approximation was used to accelerate the calculations, with the def2/J auxiliary basis set applied to further enhance computational efficiency.

For the **1b** monomer (Figure 5a), no significant structural changes were observed in either the S₀ or T₁ state, and the planarity of the platinum coordination plane was maintained. In contrast, the molecular structure at the MECP exhibited a pronounced out‐of‐plane bending at one of the imine groups, with the bending direction opposite to that of the vaulted alkyl chain. A similar trend was observed for the dimeric form of 1b: both the S₀ and T₁ states retained a planar geometry. However, the MECP structure showed a significant out‐of‐plane distortion at the imine group, with the bending direction now aligned with the vaulted chain. This reversal in bending direction relative to the monomeric MECP is likely induced by steric repulsion from the face‐to‐face stacked neighboring molecule in the dimer, which hinders distortion toward the thermodynamically favorable direction. These characteristic molecular structures in each excited state were also observed in **1d** (Figure 5b). These characteristic structural features in each electronic state were also observed for **1d**, which exhibited a similar pattern of distortion at the MECP (Figure 5b). Notably, this MECP‐specific distortion is consistent with our previous findings that dimer formation enhances phosphorescence in related vaulted Pt(II) complexes,^[^ ^15a,c,k^ ^]^ and also aligns with theoretical predictions of excited‐state distortion pathways in such systems.^[^ ^15m^ ^]^

The energies of the T_1_ and MECP states for monomeric **1b** are 50.6 and 63.1 kcal/mol, relative to the S_0_ energy, respectively, with an energy gap of 12.5 kcal/mol (Figure 5c, left). For dimeric **1b**, the corresponding values are 51.4 and 87.6 kcal/mol, with an energy gap of 36.2 kcal/mol, which is significantly larger than that of the monomer (Figure 5c, right). This trend is also observed in **1d**, as shown in Figure 5d. For monomeric **1d**, the T_1_ and MECP energies are 51.1 and 66.1 kcal/mol, respectively, with an energy gap of 15.0 kcal/mol, and the corresponding values for the dimer are 51.7 and 83.0 kcal/mol, with an energy gap of 31.3 kcal/mol. In both **1b** and **1d**, although the T_1_ state energies between the monomer and dimer do not differ significantly, the MECP energies in the dimers are much higher. This increase in MECP energy is attributed to the geometric constraint imposed by the face‐to‐face dimer arrangement, which prevents the imine group from adopting the thermodynamically favorable out‐of‐plane bending direction observed in the monomeric MECP. In contrast, the packing structures of the nonemissive crystals lacking such dimeric interactions provide sufficient spatial freedom around the imine moiety to accommodate the distortion required at the MECP (Figures S35–S39). As a result, the dimeric structure must distort along a less favorable coordinate, thereby raising the MECP energy barrier and suppressing nonradiative decay pathways, which contributes to the observed solid‐state phosphorescence (see Figure S40 for comparison with monomeric MECP geometries of **1a**, **1c**, and **2**).

**Figure 6:**
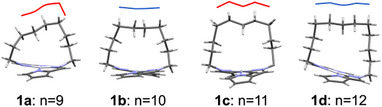
The front view of complexes **1a**‐**d** as determined by XRD analysis, with emphasis lines to highlight the carbon atoms at the top of the vaulted chains.

The odd‐even effect, where strongly emissive crystals are observed for **1b** and **1d** (even‐numbered polymethylene chains) but not for **1a** and **1c** (odd‐numbered), may arise from the relative stability of the dimer structures in the crystalline state. As shown in Figure 6, the top parts of the vaulted chains are relatively flat in even‐numbered complexes but exhibit a zigzag arrangement in odd‐numbered ones. This difference may influence the efficiency of van der Waals and C─H─π interactions between adjacent molecules in the dimer. While a more detailed energetic evaluation is necessary, these structural trends offer a plausible rationale for the observed odd‐even effect in emission behavior.

**Figure 7:**
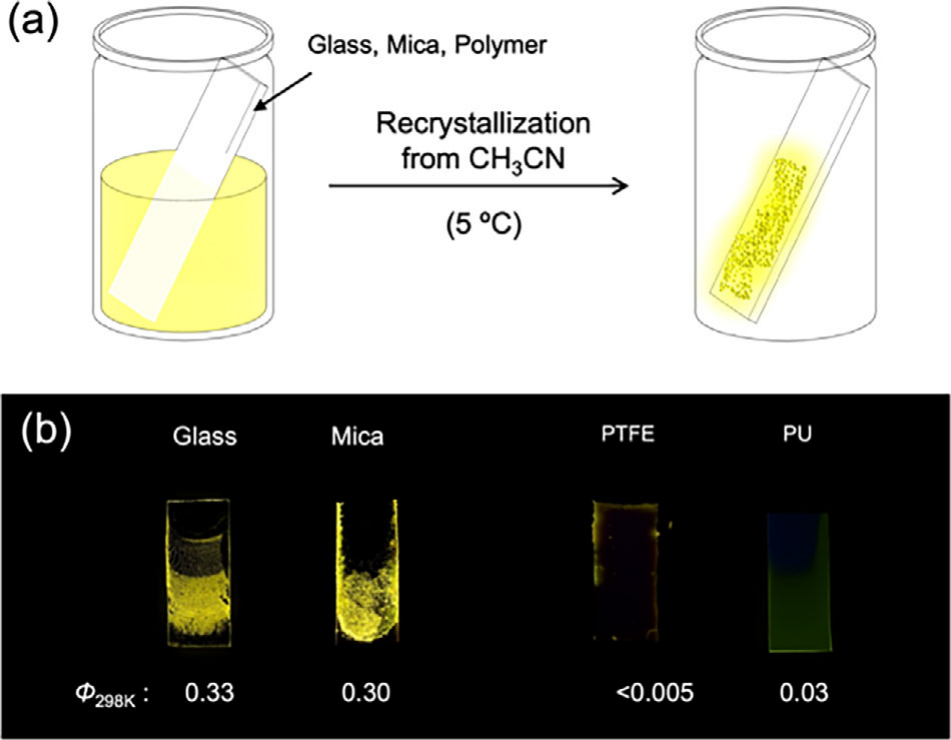
a) Schematic illustration of the crystallization methods for *rac*‐**1d** on plates of different substrates. b) Photographs and quantum yield of the aggregate on the plates under UV irradiation (365 nm).

The material used for crystallization also affects the emission intensity in the racemic mixtures of **1**, and crystallizations on substrates other than glass plates were investigated. The plates made of glass, mica, polytetrafluoroethylene (PTFE), and polyurethane (PU) were placed in a test tube, and crystals of *rac*‐**1d** were grown on their surfaces by slowly evaporating the solution at 5 °C (Figure 7a). Strongly emissive crystals were obtained on the glass and mica plates (*Φ* = 0.33 and 0.30, respectively), whereas nonemissive aggregates formed on the PTFE and PU plates (Figure 7b). This material‐dependent contrast in luminescence, due to the unique sensitivity of the molecule to crystallization conditions, highlights its potential utility in imaging and sensing applications.

**Figure 8:**
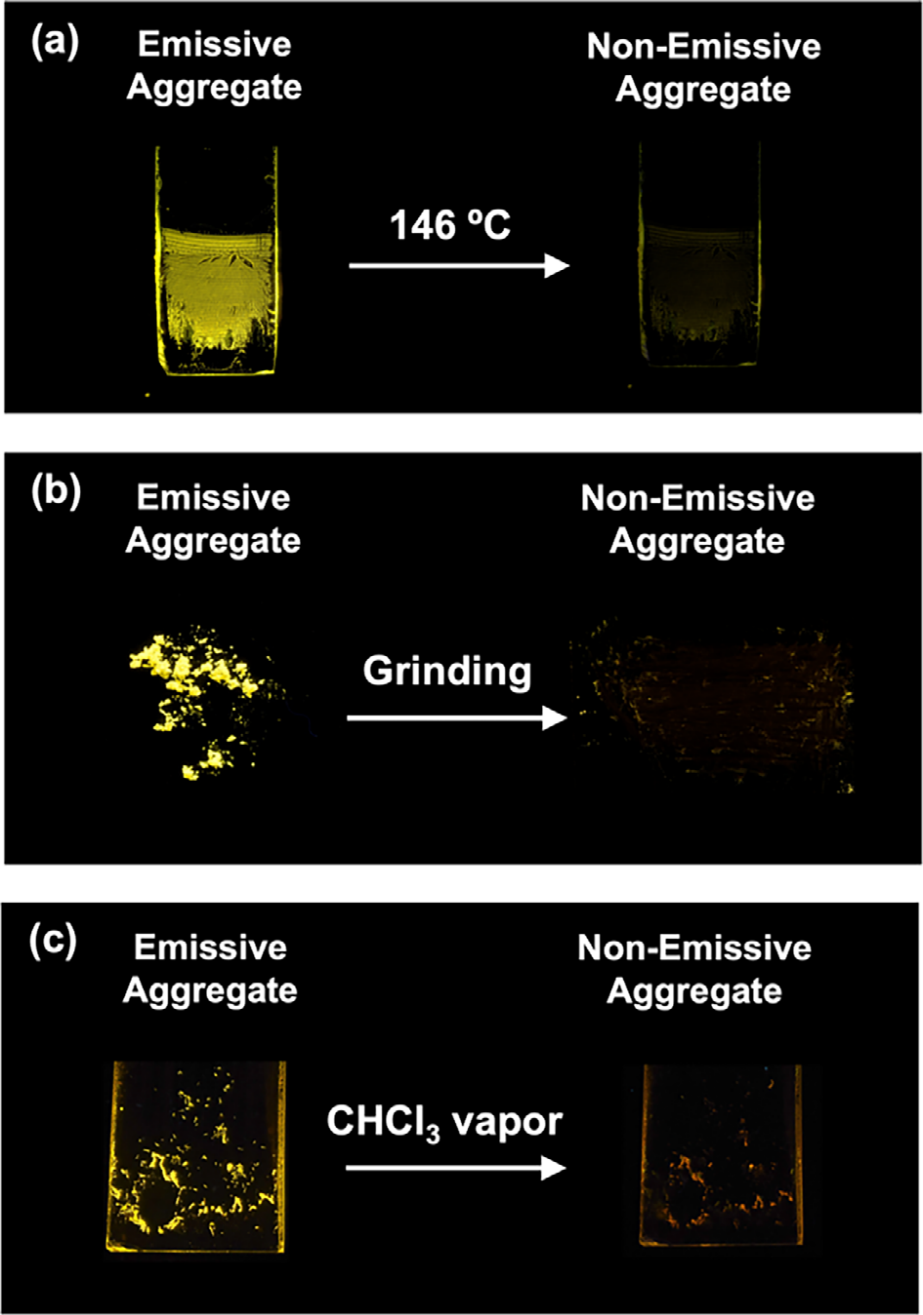
The photos of the emission quenching experiment of the emissive aggregate of *rac*‐**1d** by using a) heat, b) grinding stimuli, and c) chloroform vapor. These photos were taken at room temperature under UV irradiation (365 nm).

The emissive crystals of *rac*‐**1d** can be transformed into nonemissive aggregates by external stimuli such as heat, mechanical force, and solvent vapor. Heating the emissive crystal of *rac*‐**1d** to 150 °C followed by cooling to room temperature yields a nonemissive aggregate without decomposition (Figure 8a). This suggests that the emissive crystal is a thermodynamically metastable state, while the nonemissive form represents a more stable state, as further supported by DSC analysis (Figure S41). Moreover, the emission of *rac*‐**1d** crystals can be turned off by grinding with a spatula, indicating mechano‐responsive behavior (Figure 8b and Figure S42). The exposure of the emissive crystal of *rac*‐**1d** to chloroform vapor transforms it into a nonemissive aggregate (Figure 8c and Figure S43). These stimulus‐induced changes in emission behavior indicate that the crystal of *rac*‐**1d** is responsive to external stimuli. However, the transition from the emissive to the nonemissive state was found to be irreversible under the conditions tested. While this limits the reversibility of the material, it still suggests potential utility in certain types of sensing applications.

## 3 Conclusion

In summary, we developed trans‐bis(iminopyrrolato)platinum(II) complexes featuring vaulted polymethylene bridges, whose solid‐state emissive behavior is highly sensitive to molecular structure, crystallization conditions, chirality, and even the substrate surface. Strong yellow phosphorescence was observed specifically under recrystallization conditions using acetonitrile at 5 °C, with racemic complexes bearing even‐numbered polymethylene chains (**1b**: C10 and **1d**: C12) deposited on glass substrates. In contrast, the nonvaulted analog consistently showed no emission under any tested conditions, highlighting the essential role of the vaulted structure in enabling emissive molecular packing. DFT calculations revealed that the face‐to‐face dimer packing in the emissive crystals raises the MECP energy and thereby suppresses nonradiative decay, leading to enhanced phosphorescence. This emissive state is metastable and can be reversibly switched to a thermodynamically stable, nonemissive state via heat, mechanical force, or solvent vapor. These findings highlight a novel approach to activating phosphorescence in platinum complexes through steric control of molecular packing using vaulted polymethylene chains. This strategy not only advances the understanding of AIEE in transition‐metal complexes but also offers new possibilities for designing smart luminescent materials for sensing and imaging applications.

## Conflict of Interest

The authors declare no conflict of interest.

## Supporting information



Supporting Information

## Data Availability

The data that support the findings of this study are available in the supplementary material of this article.
