# Supplementary material for: Aggregation‐Induced Phosphorescence of a Trans‐Bis(iminomethylpyrolato)Platinum Complex Bearing a Polymethylene Vaulted Structure: Chain Length–Dependent Solid‐State Emissions
Source: Chemistry. 2025 Jul 31;31(45):e01670. doi: 10.1002/chem.202501670 (PMC12351440; doi:10.1002/chem.202501670)
Supplement: Supplementary file 1 — Supporting Information [file CHEM-31-e01670-s001.docx]

Contents

**Experimental Information** 3

[**Figure S1.** ^1^H (500 MHz) and ^13^C NMR spectra (125 MHz) of **1a** in CDCl_3_. 7](#_Toc201433010)

[**Figure S2.** ^1^H (500 MHz) and ^13^C NMR spectra (125 MHz) of **1b** in CDCl_3_. 8](#_Toc201433011)

[**Figure S3.** ^1^H (500 MHz) and ^13^C NMR spectra (125 MHz) of **1c** in CDCl_3_. 9](#_Toc201433012)

[**Figure S4.** ^1^H (500 MHz) and ^13^C NMR spectra (125 MHz) of **1d** in CDCl_3_. 10](#_Toc201433013)

[**Figure S5.** ^1^H (500 MHz) and ^13^C NMR spectra (125 MHz) of **2** in CDCl_3_. 11](#_Toc201433014)

[**Figure S6.** COSY spectrum of **1a** in CDCl_3_ (298 K, 500 MHz). 12](#_Toc201433015)

[**Figure S7.** COSY spectrum of **1b** in CDCl_3_ (298 K, 500 MHz). 13](#_Toc201433016)

[**Figure S8.** COSY spectrum of **1c** in CDCl_3_ (298 K, 500 MHz). 14](#_Toc201433017)

[**Figure S9.** COSY spectrum of **1d** in CDCl_3_ (298 K, 500 MHz). 15](#_Toc201433018)

[**Figure S10.** COSY spectrum of **2** in CDCl_3_ (298 K, 500 MHz). 16](#_Toc201433019)

[**Figure S11.** NOESY spectrum of **1a** in CDCl_3_ (298 K, 500 MHz). 17](#_Toc201433020)

[**Figure S12.** NOESY spectrum of **1b** in CDCl_3_ (298 K, 500 MHz). 18](#_Toc201433021)

[**Figure S13.** NOESY spectrum of **1c** in CDCl_3_ (298 K, 500 MHz). 19](#_Toc201433022)

[**Figure S14.** NOESY spectrum of **1d** in CDCl_3_ (298 K, 500 MHz). 20](#_Toc201433023)

[**Figure S15.** NOESY spectrum of **2** in CDCl_3_ (298 K, 500 MHz). 21](#_Toc201433024)

[**Figure S16.** HMQC spectrum of **2** in CDCl_3_ (298 K, 500 MHz). 22](#_Toc201433025)

[**Figure S17.** UV–vis absorption spectra of *rac*-**1a**–**d** and **2** in 2-MeTHF (1.0 × 10⁻⁴ M). 23](#_Toc201433026)

[**Figure S18.** Solid-state UV–vis absorption spectra of complexes *rac*-**1a**–**d** and **2**. 23](#_Toc201433027)

[**Figure S19.** Solid-state UV–vis absorption spectra of *rac*-**1d** crystals. 24](#_Toc201433028)

[**Figure S20.** Solid-state UV–vis absorption spectra of **2** crystals. 24](#_Toc201433029)

[**Figure S21.** Normalized excitation spectra of *rac*-**1d** crystals. 25](#_Toc201433030)

[**Figure S22.** Normalized excitation spectra of **2** crystals. 25](#_Toc201433031)

[**Figure S23.** Normalized emission spectra of *rac*-**1a**–**d** and **2** in 2-MeTHF (1.0 × 10⁻⁴ M) at 77 K. 26](#_Toc201433032)

[**Figure S24.** Normalized emission spectra of *rac*-**1a** in solution and crystalline states at room temperature. 26](#_Toc201433033)

[**Figure S25.** Normalized emission spectra of *rac*-**1b** in solution and crystalline states at room temperature. 27](#_Toc201433034)

[**Figure S26.** Normalized emission spectra of *rac*-**1c** in solution and crystalline states at room temperature. 27](#_Toc201433035)

[**Figure S27.** Photographs of *rac*-**1d** in H₂O/THF mixed solvents (1.0 × 10⁻⁴ M). 28](#_Toc201433036)

[**Figure S29.** Molecular orbital energy diagrams of dimer of **1b** and **d**. 29](#_Toc201433037)

[**Figure S30.** Selected molecular orbitals of **1a**–**d** relevant to T₁. 30](#_Toc201433038)

[**Figure S31.** Selected molecular orbitals of dimer of **1b** and **d** relevant to T₁. 31](#_Toc201433039)

[**Table S1.** Calculated energies of selected molecular orbitals for **1a**–**d**. 32](#_Toc201433040)

[**Table S2.** Calculated energies of selected molecular orbitals for dimer of **1b** and **d**. 32](#_Toc201433041)

[**Table S3.** Photophysical data for *rac*-**1a**–**1d** and **2** in 2-MeTHF (1.0 × 10^–4^ M). 33](#_Toc201433042)

[**Table S4.** Photophysical data of *rac*-**1a**–**1d** and **2** in crystalline state. 34](#_Toc201433043)

[**Figure S32.** Photographs of (*S*) and (*R*)-**1a**–**d** crystals (recrystallized from acetonitrile at 5 ^o^C). 35](#_Toc201433044)

[**Figure S33.** Crystal packing structure of *rac*-**1d** obtained from acetonitrile at 25 and 60 °C. 36](#_Toc201433045)

[**Figure S34.** Crystal packing structure of **2** obtained from acetonitrile at 5 °C. 37](#_Toc201433046)

[**Figure S35.** Intermolecular interactions observed around the Pt(II) planes in the crystal packing of *rac*-**1a**. 38](#_Toc201433047)

[**Figure S36.** Intermolecular interactions observed around the Pt(II) planes in the crystal packing of *rac*-**1b**. 39](#_Toc201433048)

[**Figure S37.** Intermolecular interactions observed around the Pt(II) planes in the crystal packing of *rac*-**1c**. 40](#_Toc201433049)

[**Figure S38.** Intermolecular interactions observed around the Pt(II) planes in the crystal packing of *rac*-**1d**. 41](#_Toc201433050)

[**Figure S39.** Intermolecular interactions observed around the Pt(II) planes in the crystal packing of (*S*)-**1b**, (*R*)-**1c**, and (*R*)-**1d**. 42](#_Toc201433051)

[**Figure S40.** DFT-calculated molecular structures and energy diagrams of S₀, T₁ and MECP states for complexes **1a**, **1c** and **2**. 43](#_Toc201433052)

[**Figure S41.** Differential scanning calorimetry diagram of *rac*-**1d** crystal showing the solid–solid transition. 44](#_Toc201433053)

[**Figure S42.** Powder X-ray diffraction (PXRD) patterns of *rac*-**1d** under different conditions (stimuli-responsive behavior upon grinding). 45](#_Toc201433054)

[**Figure S43.** Powder X-ray diffraction (PXRD) patterns of *rac*-**1d** under different conditions (stimuli-responsive behavior upon CHCl₃ vapor). 45](#_Toc201433055)

[**Figure S44.** Emission decay curves for *rac*-**1a**–**d** and **2** in 2-MeTHF (1.0 × 10⁻⁴ M). 46](#_Toc201433056)

[**Figure S45.** Emission decay curves for crystals of *rac*-**1a–1d** obtained from acetonitrile. 47](#_Toc201433057)

[**Table S5.** Crystallographic data for *rac*-**1a**–**1d (**crystal obtained from acetonitrile at 5 °C). 48](#_Toc201433058)

[**Table S6.** Crystallographic data for (*S*)-**1b**, (*R*)-**1c**, (*R*)-**1d** and **2** **(**crystal obtained from acetonitrile at 5 °C). 49](#_Toc201433059)

[**Table S7.** Crystallographic data for *rac*-**1a** crystals obtained from acetonitrile at 25 and 60 °C. 50](#_Toc201433060)

[**References** 51](#_Toc201433061)

**Experimental Information**

**General**: Melting points were measured on a glass plate on Yanagimoto micro melting point apparatus. IR spectroscopy was performed using a Jasco FT/IR-410 spectrometer (for **1b–1d** and **2**) and a FT/IR-460 plus (JASCO Co., Ltd.) with an ATR PRO450-S accessory (JASCO Co., Ltd.) (for **1a**). ^1^H, ^13^C NMR, COSY, and NOESY spectra for **1b–1d** and **2** were recorded on a Varian Unity–Inova 500 spectrometer (500 MHz for ^1^H NMR, 125 MHz for ^13^C NMR). ^1^H and ^13^C NMR, COSY, and NOESY spectra for **1a** were recorded on a JEOL ECZ-500 (500 MHz for ^1^H NMR, 125 MHz for ^13^C NMR). Chemical shifts are denoted in *δ*-unit (ppm) relative to tetramethylsilane. The splitting patterns are designated as follows: s (singlet), d (doublet), t (triplet), q (quartet), m (multiplet), and br (broad). Preparative GPC was performed using a Japan Analytical Industry LaboACE/LC-5060 instrument equipped with two JAIGEL-2HR columns. Gel permeation chromatography was performed using a recycling preparative HPLC system (Japan Analytical Industry, LC-908) equipped with a JAIGEL-2H column. High-resolution mass spectroscopy (HRMS) was performed using a Bruker micrOTOF II-OCU spectrometer. UV-vis spectra were obtained using a Jasco V650 spectrometer. Emission spectra were acquired with a Jasco FP-6500 spectrometer. Absolute quantum yields were determined using a Jasco FP-6500 spectrometer equipped with a Jasco ISN-470 integrating sphere. Emission lifetime measurements were conducted using a system including a Continuum PowerPrecision 9010 Nd:YAG laser, a Princeton Instruments Acton SP 2300 monochromator, a Hamamatsu R928 photomultiplier tube and a Tektronix TDS220 digital oscilloscope. All solution phase photophysical measurements were performed using fully dissolved, visually transparent samples in 2-MeTHF. Elemental analyses were performed by using a PerkinElmer 2400II CHN elemental analyzer. Optical rotation was measured on a Jasco DIP-370 digital polarimeter.

**X-ray structure determination**: Crystals employed for X-ray diffraction studies were obtained by recrystallization from acetonitrile solution (3.65 mM) for vaulted complexes **1a–d** and saturated acetonitrile solution for non-vaulted complex **2**, and data were collected using a Rigaku XtaLAB P-200 diffractometer with graphite monochromated Mo Kα radiation (λ = 0.71075 Å). Their structures were solved by direct methods and refined using the full-matrix least-squares method. In the subsequent refinement, the function ∑ω(Fo^2^–Fc^2^)^2^ was minimized, where Fo and Fc are the observed and calculated structure factor amplitudes, respectively. The positions of non-hydrogen atoms were determined from difference Fourier electron-density maps and refined anisotropically. All the ORTEP illustrations were generated using ORTEP-3.

**Computational Methods:** All computations were performed using the ORCA 6.0.0 package (ref 18). Geometry optimizations and vibrational frequency calculations were carried out for 1 and 2 in the singlet ground state, triplet excited state, and minimum energy crossing point (MECP), using density functional theory (DFT) with the CAM-B3LYP functional and the def2-TZVP basis set for all atoms. The RIJCOSX approximation with the def2/J auxiliary basis set was employed to reduce computational cost. Unless otherwise noted, all calculations were performed using ORCA’s default integration grids and SCF convergence criteria. Vibrational frequencies were calculated analytically for the optimized singlet and triplet geometries to confirm the nature of the stationary points and to obtain zero-point vibrational energy (ZPVE) and thermochemical corrections. For the MECP geometries, numerical frequency calculations were used due to limitations in ORCA. MECP structures were located using the built-in procedure to locate the crossing point between the lowest singlet and triplet states. Time-dependent DFT (TDDFT) calculations were also performed at the same level of theory (CAM-B3LYP/def2-TZVP with RIJCOSX and def2/J) to estimate the electronic excited states of the optimized structures.

**Materials:** PtCl_2_(CH_3_CN)_2_ was prepared according to the reported procedure (ref 19). Pyrrole-2-carboxaldehyde (Fujifilm Wako Pure Chemical), 1,9-diaminodecane (Wako), 1,10-diaminodecane (Wako), 1,11-diaminoundecane (TCI), 1,12-diaminododecane (Wako), 1-decylamine (Fujifilm Wako Pure Chemical), ethanol, potassium carbonate, dimethyl sulfoxide, toluene, *n*-hexane, and ethyl acetate were commercially available and used without further purification. *N,N'*-bis[(1*H*-pyrrol-2-yl)methylene]-1,*ω*-alkanediamines and 1-[(1*H*-pyrrol-2-yl)methyleneamino]decane were prepared by the reaction of pyrrole-2-carboxaldehyde with the corresponding 1,*ω*-alkanediamines and decylamine by refluxing in EtOH.

**Synthesis of 1a**–**1d**: A series of complexes **1** was prepared by stirring a mixture of PtCl_2_(CH_3_CN)_2_ (5.00 mmol), *N,N'*-Bis[(1*H*-pyrrol-2-yl)methylene]-1,*ω*-alkanediamines (5.00 mmol), and K_2_CO_3_ (32.5 mmol) in DMSO (50.0 mL) and toluene (200 mL) at 140 °C for 24 h. The resulting mixture was filtered, and the toluene was removed under reduced pressure. chloroform (80.0 mL) was then added to the residue, and the solution was washed with water (100 mL × 3) and brine (100 mL × 2), followed by drying over MgSO_4_. After removing the solvent under reduced pressure, the resulting crude product was purified by column chromatography (SiO₂, chloroform/hexane = 1:10) and GPC (CH_2_Cl_2_) to afford complexes **1a–d**. The optically pure complexes (*R*)- and (*S*)-**1a–d** were obtained from the racemic mixture using a recycling preparative HPLC system (Japan Analytical Industry, LC-9201) with a Daicel Chiralpak IA column [chloroform: Hexane (2 : 3)]. The absolute configurations of (+) and (–)-**1b**–**d** were unambiguously determined by single-crystal X-ray diffraction analysis as (+)-(*R*) and (–)-(*S*), respectively. The configuration of (+) and (–)-**1a** was assigned by analogy based on the elution order in chiral HPLC. In all cases, the first-eluted enantiomer was identified as (–)-(*S*), and the second-eluted as (+)-(*R*) under the employed separation conditions.

**Synthesis of 2**: A complex **2** was prepared by stirring a mixture of PtCl_2_(CH_3_CN)_2_ (2.80 mmol), 1-[(1*H*-pyrrol-2-yl)methyleneamino]decane (5.60 mmol), and K_2_CO_3_ (18.2 mmol) in DMSO (14.0 mL) and toluene (56.0 mL) at 140°C for 24 h. The resulting mixture was filtered and extracted with EtOAc (100 mL × 2), washed with brine (100 mL × 3), and followed by drying over MgSO_4_. After removing the solvent under reduced pressure, the resulting crude product was purified by column chromatography (SiO₂, chloroform) and GPC (CH_2_Cl_2_) to afford complex **2**.


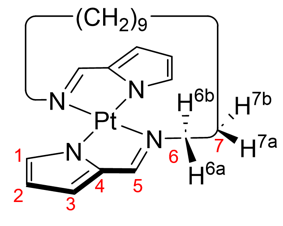
For *rac*-**1a**: Yellow solid (1%); m.p. 158–159 °C; IR (ATR): 2924, 2853, 1578, 1456, 1391, 1337, 1318, 1213, 1038, 727 cm^-1^; ^1^H NMR (CDCl_3_, 500 MHz): *δ* 1.21–1.31 (m, 4H), 1.32–1.40 (m, 2H), 1.42–1.50 (m, 2H), 1.60–1.74 (m, 4H), 2.10–2.20 (m, 2H, H^7b^), 3.38 (ddd, *J* = 12.2, 12.2, 3.9 Hz, 2H, H^6a^), 4.34 (ddd, *J* = 12.2, 3.9, 3.9 Hz, 2H, H^6b^), 6.30 (dd, *J* = 3.8, 1.8 Hz, 2H, H^2^), 6.75 (dd, *J* = 3.8, 0.9 Hz, 2H, H^3^), 7.11 (br, 2H, H^1^), 7.66 ((s, 1.28H) and (d, *J*_195Pt, H_ = 80 Hz, 0.72H), H^5^); ^13^C NMR (CDCl_3_, 125 MHz): *δ* 22.4, 27.3, 27.6, 29.0, 59.5 (C^6^), 111.3 (C^2^), 116.2 (C^3^), 135.8 (C^1^), 140.7 (C^4^), 162.4 (C^5^); HRMS (ESI) *m/z* calcd for C_19_H_27_N_4_^195^Pt, [M+H]^+^ 506.1878, found 506.1897. Anal. Calcd for C_19_H_26_N_4_Pt: C, 45.14; H, 5.18; N, 11.08%. Found: C, 45.40; H, 5.20; N, 11.00%.

For (*R*) and (*S*)-**1a**: (*R*)-**1a**: [*α*]_D_^27^ = +732 ± 12 (*c* 0.050, chloroform), (*S*)-**1b**: [*α*]_D_^27^ = –730 ± 10 (*c* 0.050, chloroform). M.p. for (*S*)-**1a**: 187.0–188.0 °C.


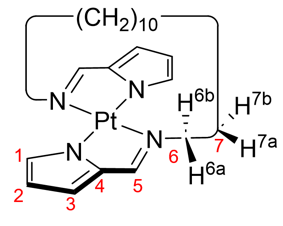
For *rac*-**1b**: Yellow solid (8%); m.p. 205–206 °C; IR (KBr): 2924, 2849, 1590, 1458, 1393, 1340, 1319, 1216, 1040, 723 cm^-1^; ^1^H NMR (CDCl_3_, 500 MHz): *δ* 0.92–1.02 (m, 2H), 1.18–1.38 (m, 6H), 1.38–1.51 (m, 4H), 1.65–1.77 (m, 2H; H^7a^), 2.34–2.48 (m, 2H, H^7b^), 3.29 (ddd, *J* = 12.5, 12.5, 1.8 Hz, 2H, H^6a^), 4.38 (ddd, *J* = 12.5, 2.8, 2.8 Hz, 2H, H^6b^), 6.29 (dd, *J* = 3.9, 2.1 Hz, 2H, H^2^), 6.74 (dd, *J* = 3.9, 0.9 Hz, 2H, H^3^), 7.10 (br, 2H, H^1^), 7.63 [(s, 1.32H) and (d, *J*_195Pt-H_ = 78 Hz, 0.68H), H^5^]; ^13^C NMR (CDCl_3_, 125 MHz): *δ* 23.2, 25.8, 27.6, 27.9, 60.4 (C^6^), 111.2 (C^2^), 116.5 (C^3^), 136.1 (C^1^), 140.8 (C^4^), 162.2 (C^5^); HRMS (FAB) *m/z* calcd for C_20_H_28_N_4_^195^Pt, [M]^+^ 519.1962, found 519.1986. Anal. Calcd for C_20_H_28_N_4_Pt: C, 46.24; H, 5.43; N, 10.78%. Found: C, 46.32; H, 5.41; N, 10.76%.
For (*R*) and (*S*)-**1b**: (*R*)-**1b**: [*α*]_D_^27^ = +712 ± 11 (*c* 0.050, chloroform), (*S*)-**1b**: [*α*]_D_^27^ = –714 ± 9 (*c* 0.050, chloroform). M.p. for (*S*)-**1b**: 207.0–208.0 °C.


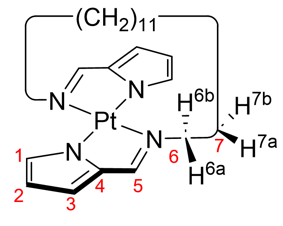
For *rac*-**1c**: Yellow solid (10%); m.p. 185–186 °C; IR (KBr): 2928, 2852, 1593, 1460, 1394, 1342, 1320, 1041, 731 cm^-1^; ^1^H NMR (CDCl_3_, 500 MHz): *δ* 1.12–1.24 (m, 4H), 1.24–1.43 (m, 8H), 1.44–1.58 (m, 4H), 2.15–2.27 (m, 2H, H^7b^), 3.30 (ddd, *J* = 12.2, 12.2, 2.5 Hz, 2H, H^6a^), 4.38 (ddd, *J* = 12.2, 3.6, 3.6 Hz, 2H, H^6b^), 6.30 (dd, *J* = 3.8, 1.9 Hz, 2H, H^2^), 6.75 (dd, *J* = 3.8, 1.0 Hz, 2H, H^3^), 7.11 (br, 2H; H^1^), 7.60 [(s, 1.32H) and (d, *J*_195Pt-H_ = 78 Hz, 0.68H), H^5^]; ^13^C NMR (CDCl_3_, 125 MHz): *δ* 24.5, 27.2, 27.4, 27.7, 29.3, 60.1 (C^6^), 110.9 (C^2^), 116.7 (C^3^), 136.0 (C^1^), 140.9 (C^4^), 162.3 (C^5^); HRMS (FAB): *m/z* calcd for C_21_H_30_N_4_^195^Pt, [M]^+^ 533.2118, found 533.2118. Anal. Calcd for C_19_H_26_N_4_Pt: C, 47.27; H, 5.67; N, 10.50%. Found: C, 47.33; H, 5.49; N, 10.34%.
For (*R*) and (*S*)-**1c**: (+)-**1c**: [*α*]_D_^27^ = +682 ± 13 (*c* 0.049, chloroform); [*α*]_D_^27^ = –684 ± 10 (*c* 0.050, chloroform). M.p. for (*S*)-**1c**: 215.0–216.0 °C.


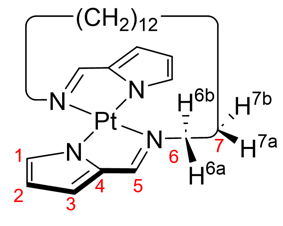
For *rac*-**1d**: Yellow solid (5%); m.p. 184–185 °C; IR (KBr): 2920, 2855, 1595, 1463, 1393, 1344, 1321, 1278, 1190, 1127, 1143, 1004, 940, 732, 681, 604 cm^-1^; ^1^H NMR (CDCl_3_, 500 MHz,): *δ* 0.92–1.03 (m, 2H), 1.05–1.15 (m, 2H*^alkyl^*), 1.17–1.42 (m, 10H), 1.43–1.53 (m, 4H), 2.17–2.27 (m, 2H, H^7b^), 3.30 (ddd, *J* = 12.6, 12.6, 1.5 Hz, 2H, H^6a^), 4.39 (ddd, *J* = 12.6, 3.0, 3.0 Hz, 2H, H^6b^), 6.18 (dd, *J* = 3.8, 2.0 Hz, 2H, H^2^), 6.73 (dd, *J* = 3.8, 0.7 Hz, 2H, H^3^), 7.09 (br, 2H, H^1^), 7.57 [(s, 1.32H) and (d, *J*_195Pt-H_ = 78 Hz, 0.68H), H^5^]; ^13^C NMR (CDCl_3_, 125 MHz): *δ* 23.0, 26.7, 27.0, 27.1, 29.0, 60.3 (C^6^), 110.7 (C^2^), 116.6 (C^3^), 136.0 (C^1^), 140.5 (C^4^), 162.3 (C^5^); HRMS (FAB): *m/z* calcd for C_22_H_32_N_4_^195^Pt, [M]^+^ 547.2277, found 547.2254. Anal. Calcd for C_22_H_32_N_4_Pt: C, 48.25; H, 5.89; N, 10.23%. Found: C, 48.40; H, 5.90; N, 10.19%.
For (*R*) and (*S*)-**1d**: (*R*)-**1d**: [*α*]_D_^27^ = +619 ± 8 (*c* 0.058, chloroform); (*S*)-**1d**: [*α*]_D_^27^ = –625 ± 10 (*c* 0.051, chloroform). M.p for (*S*)-**1d**: 222–223 °C.

For **2:** Orange solid (48%); m.p. 152–153 °C; IR (KBr): 2918, 2850, 1606, 1472, 1326, 1044, 938, 727, 681, 602 cm^–1^; ^1^H NMR (CDCl_3_, 500 MHz): *δ* 0.87 (t, *J* = 7.0 Hz, 6 H, H^15^), 1.23–1.39 (m, 28 H, H^8–14^), 1.85 (tt, *J* = 7.3, 7.3 Hz, 4 H, H^7^), 3.49 (t, *J* = 7.3 Hz, 4 H, H^6^), 6.29 (dd, *J* = 3.7, 2.0 Hz, 2 H, H^2^), 6.74 (dd, *J* = 3.7, 0.7 Hz, 2 H, H^3^), 7.05 (dd, *J* = 2.0, 0.7 Hz, 2 H, H^1^), 7.57 [(s, 1.32H) and (d, *J*_195Pt-H_ = 78 Hz, 0.68H), H^5^]; ^13^C NMR (CDCl_3_, 125 MHz): *δ* 14.1 (C^15^), 18.5 (C^14^), 22.7 (C^8^), 26.6 (C^12^), 29.3 (C^11^), 29.5, 31.2 (C^7^), 31.9 (C^13^), 60.0 (C^6^), 110.7 (C^2^), 117.2 (C^3^), 135.6 (C^1^), 140.6 (C^4^), 162.2 (C^5^); HRMS (APCI): m/z calcd for C_30_H_51_N_4_^195^Pt, [M+H]^+^ 662.3759, found 662.4323. Anal. Calcd for C_30_H_50_N_4_Pt: C, 54.44; H, 7.62; N, 8.47%. Found: C, 54.53; H, 7.86; N, 8.46%.


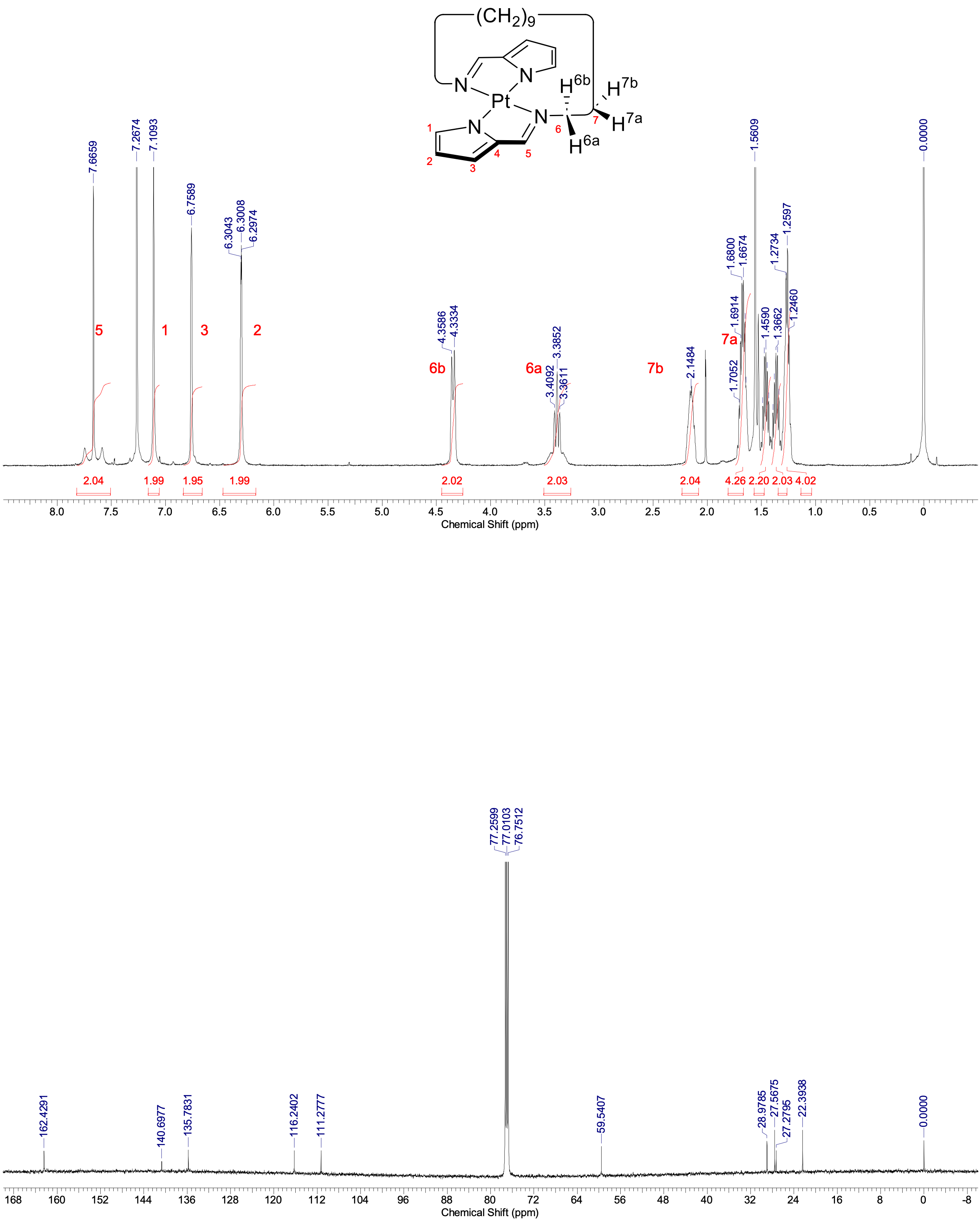


1. ^1^H (500 MHz) and ^13^C NMR spectra (125 MHz) of **1a** in CDCl_3_.


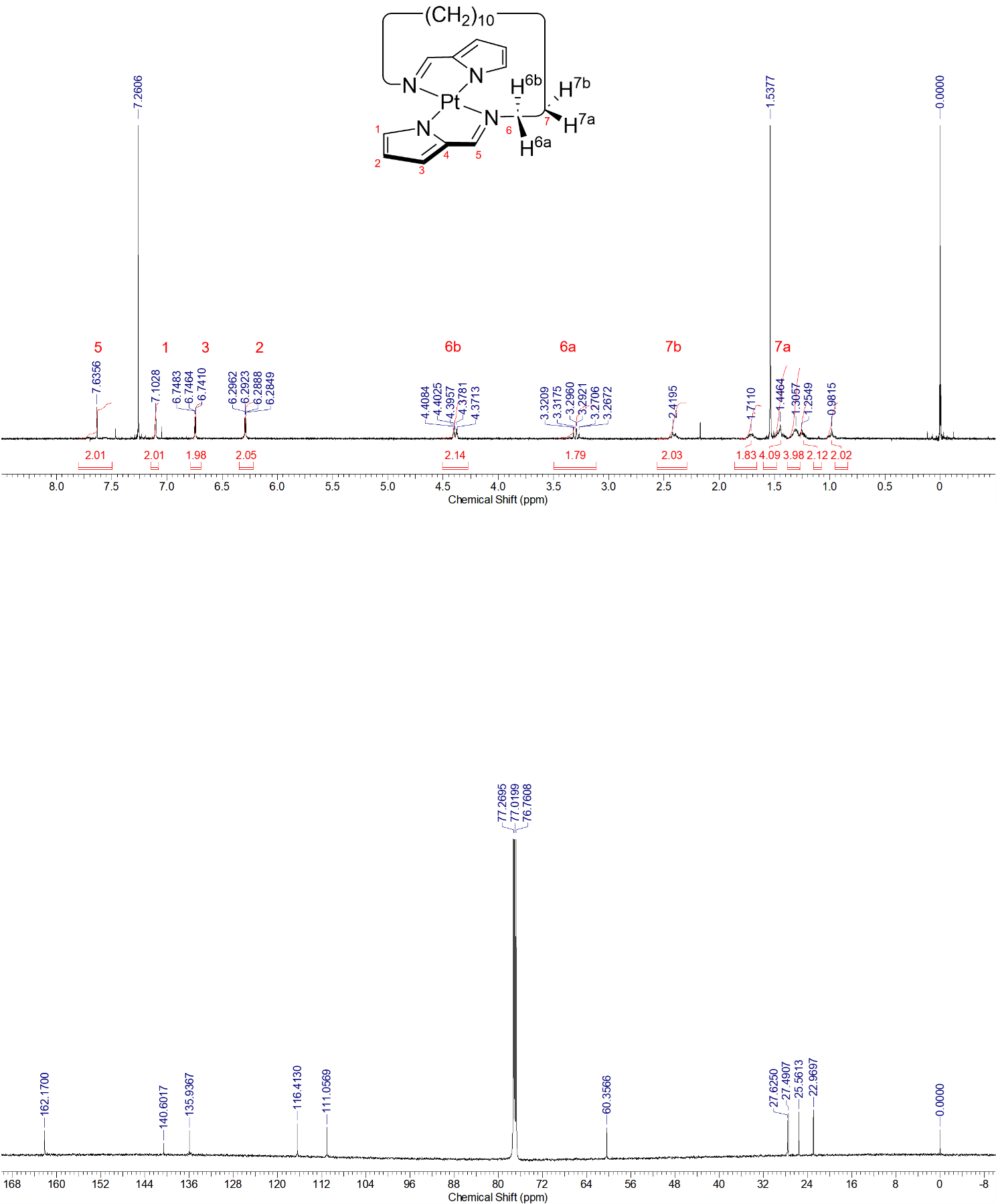


1. ^1^H (500 MHz) and ^13^C NMR spectra (125 MHz) of **1b** in CDCl_3_.


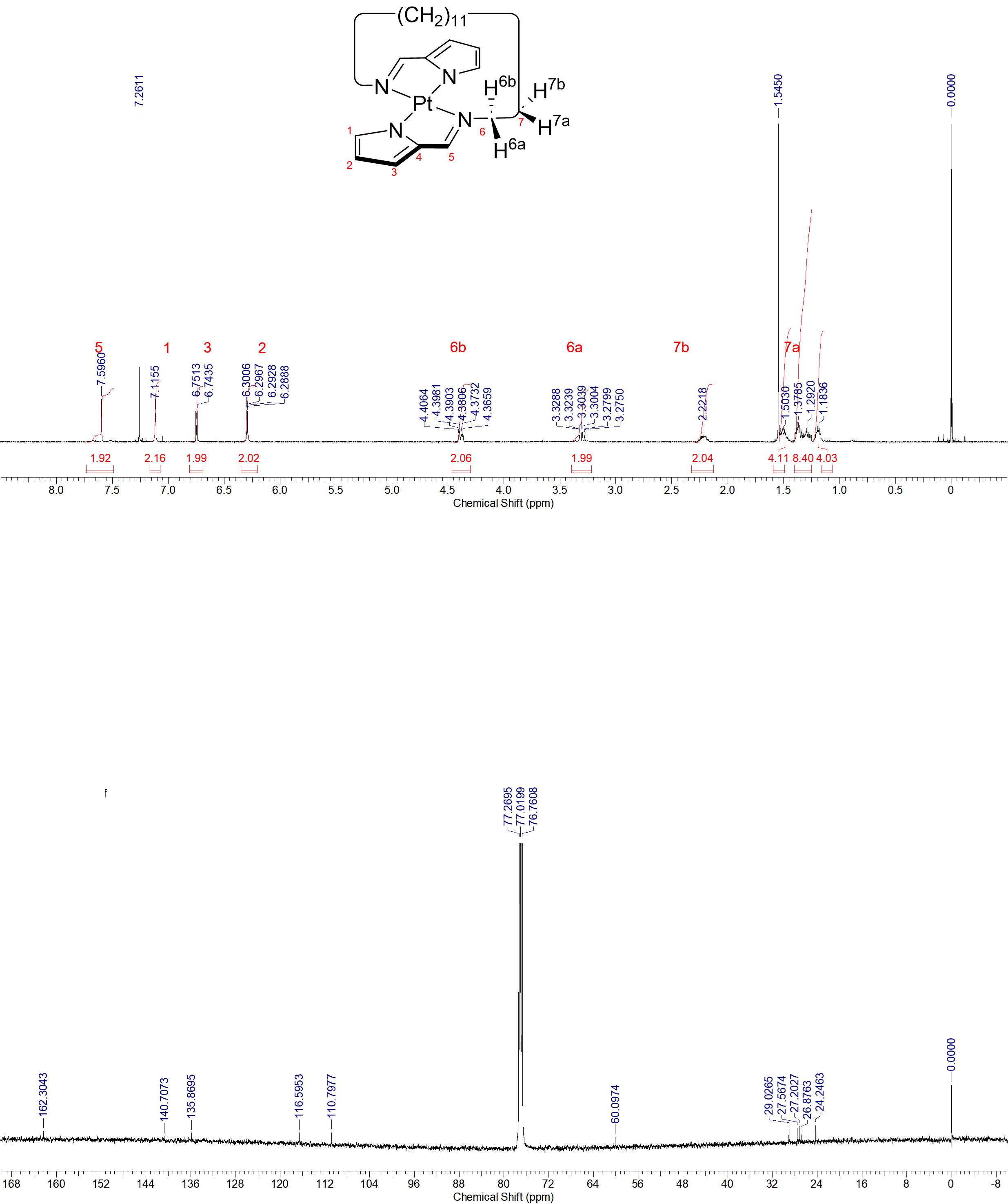


1. ^1^H (500 MHz) and ^13^C NMR spectra (125 MHz) of **1c** in CDCl_3_.


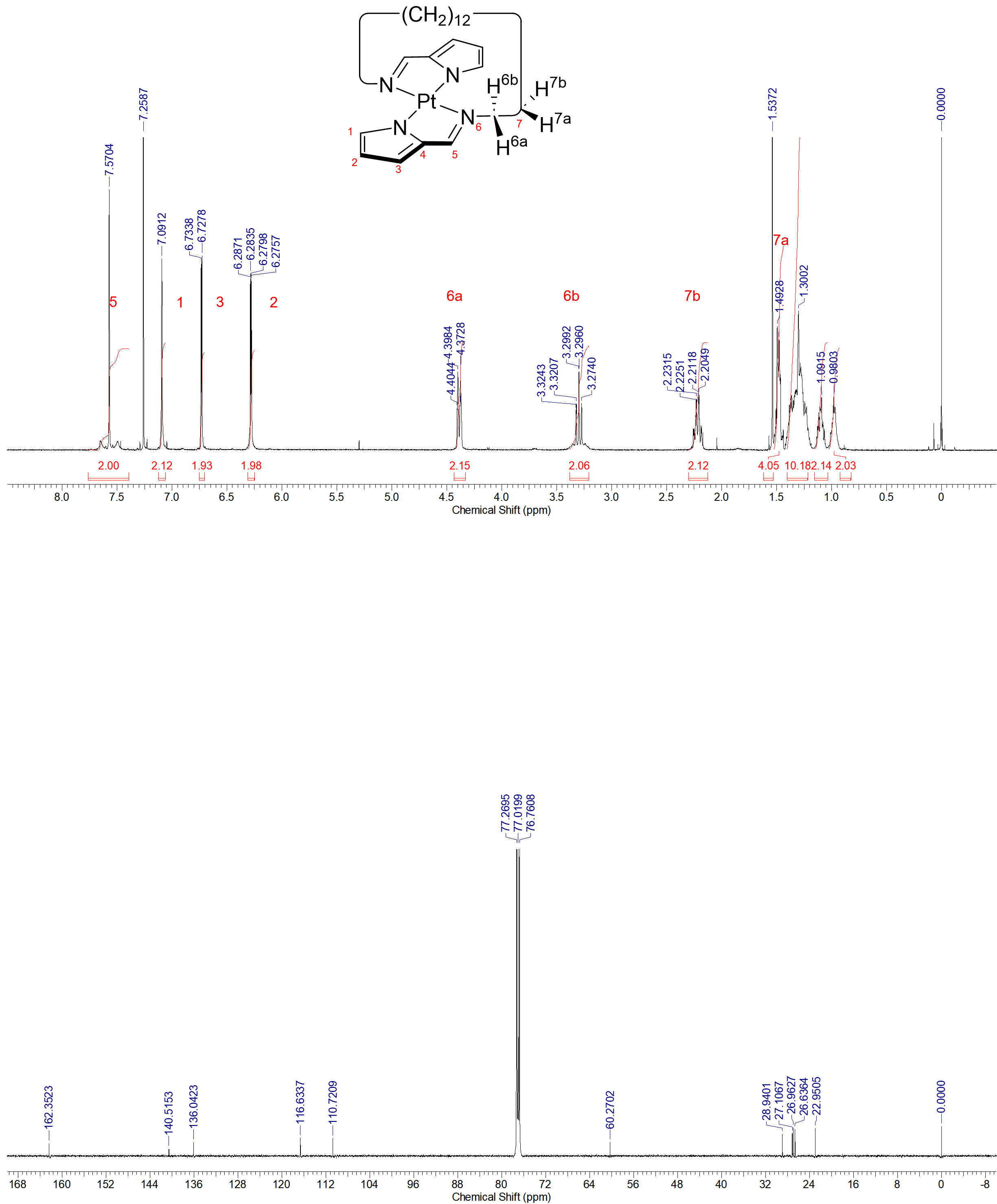


1. ^1^H (500 MHz) and ^13^C NMR spectra (125 MHz) of **1d** in CDCl_3_.


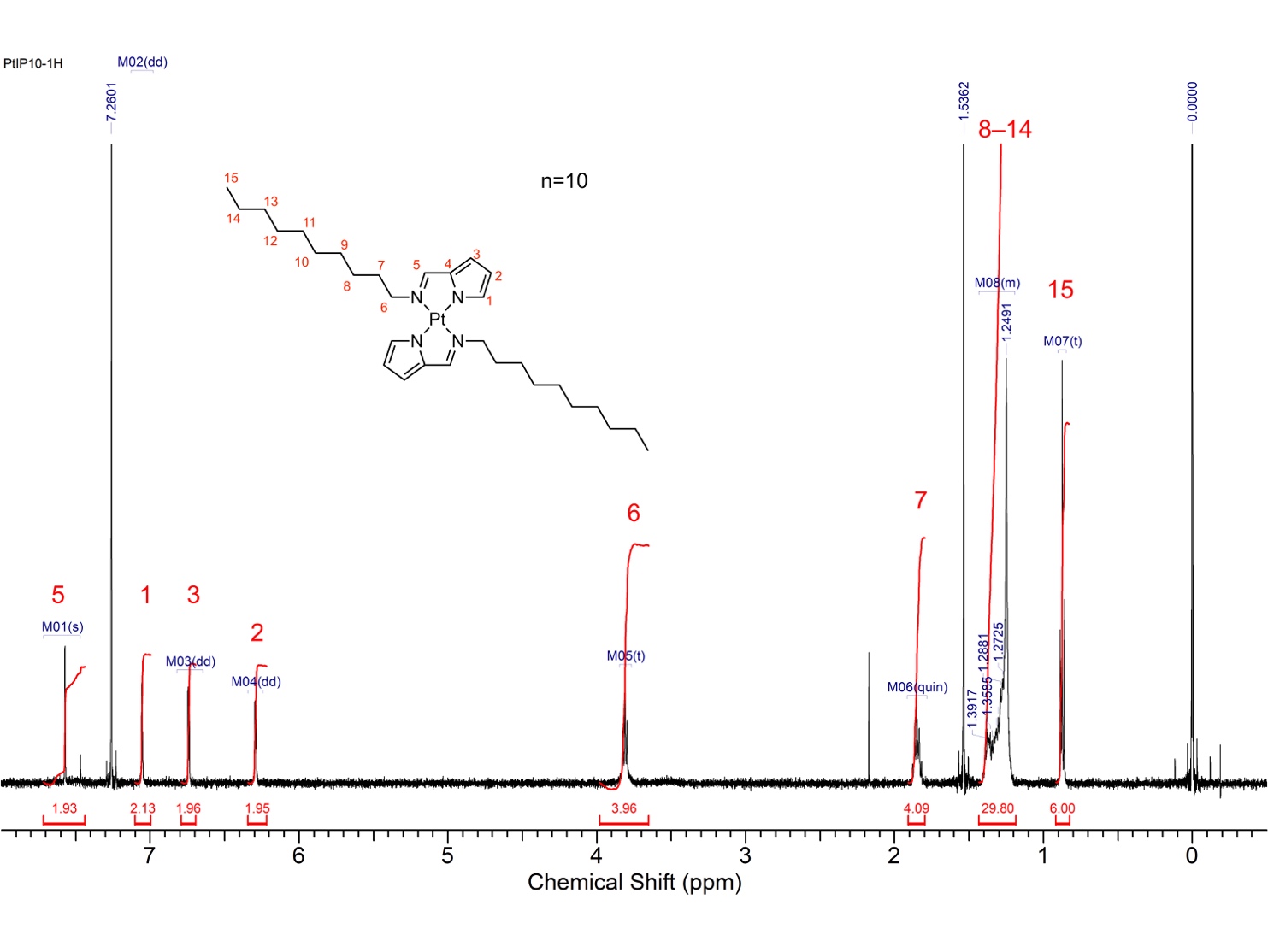


**
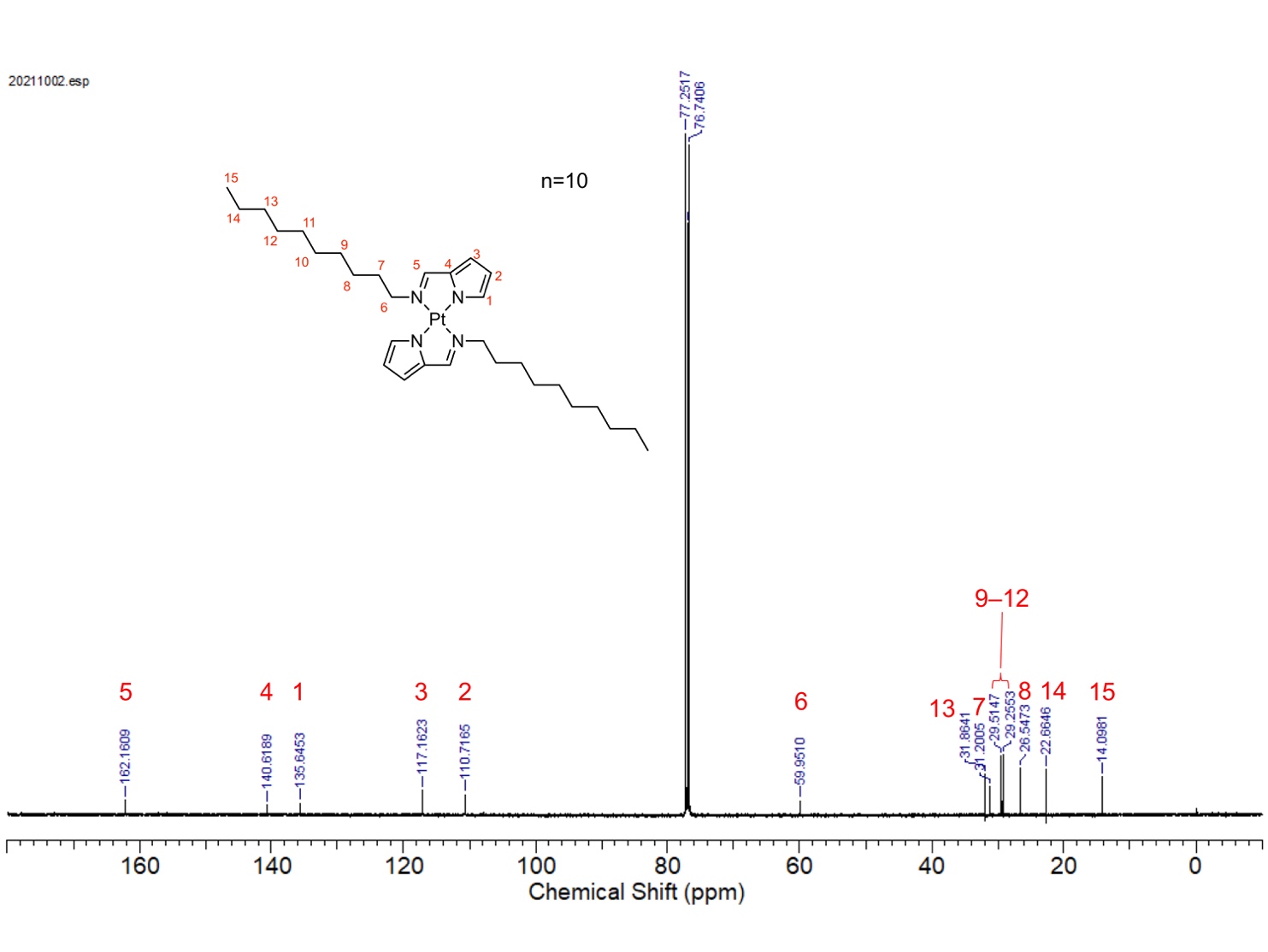
**

1. ^1^H (500 MHz) and ^13^C NMR spectra (125 MHz) of **2** in CDCl_3_.


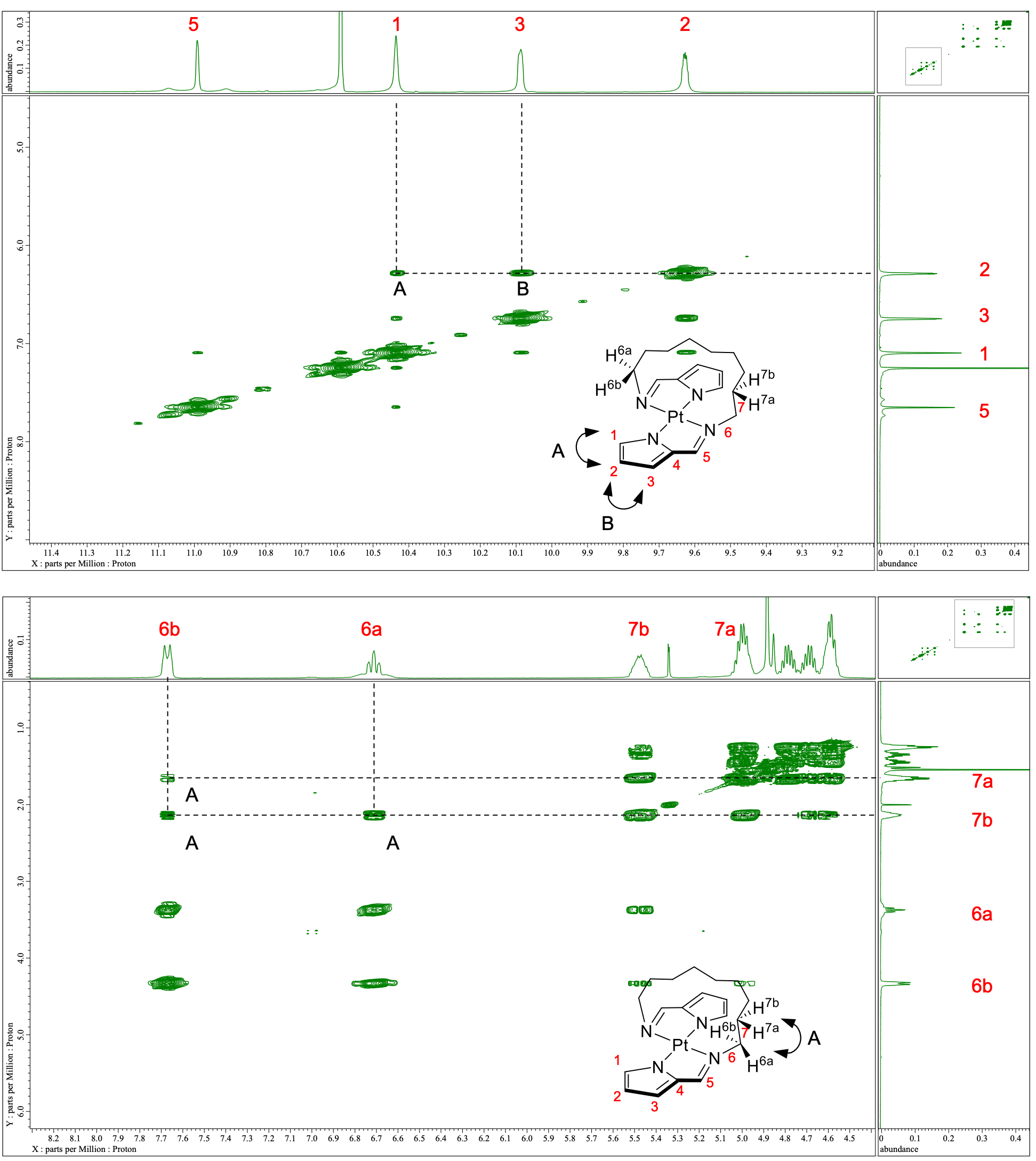


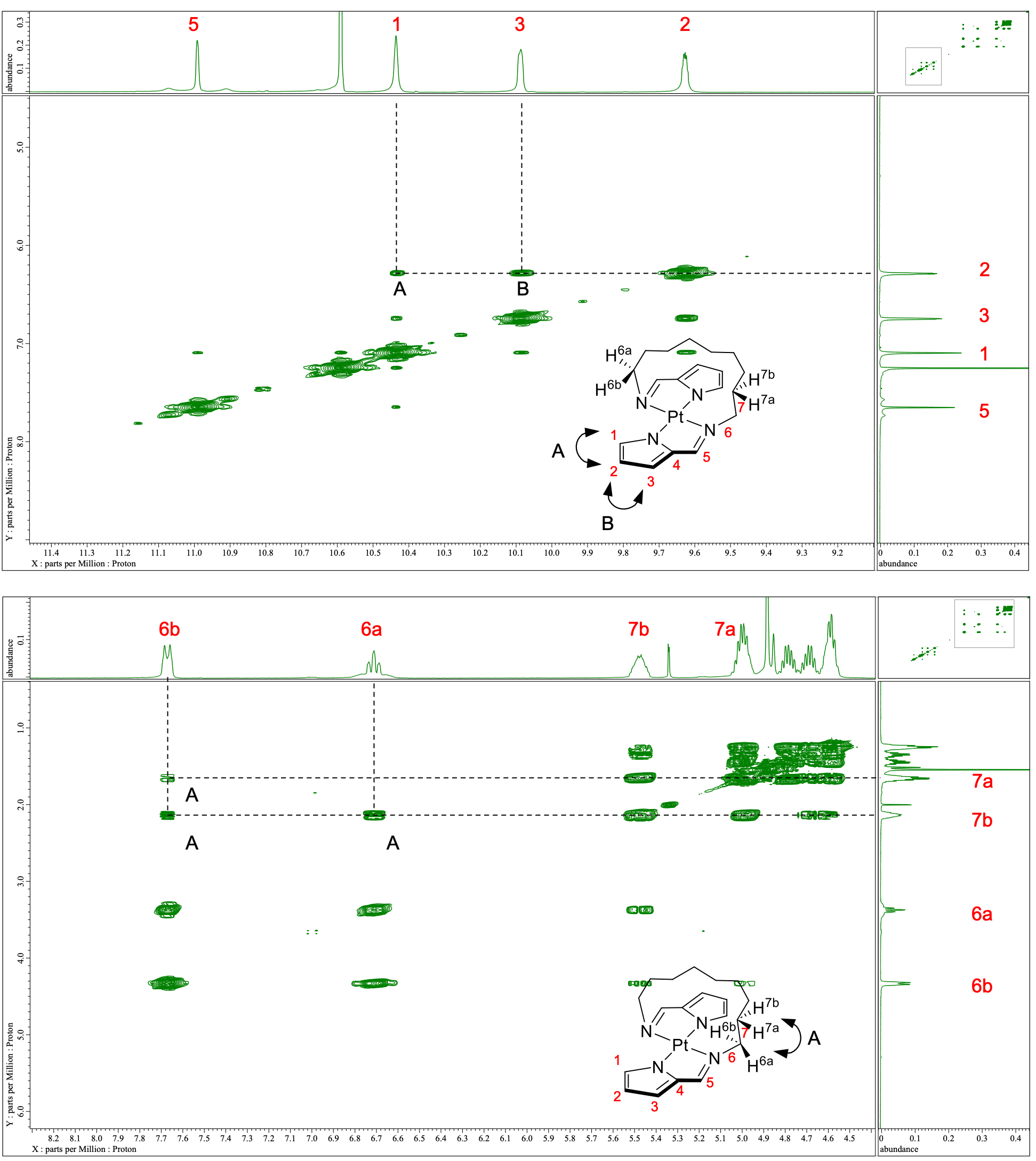


1. COSY spectrum of **1a** in CDCl_3_ (298 K, 500 MHz).


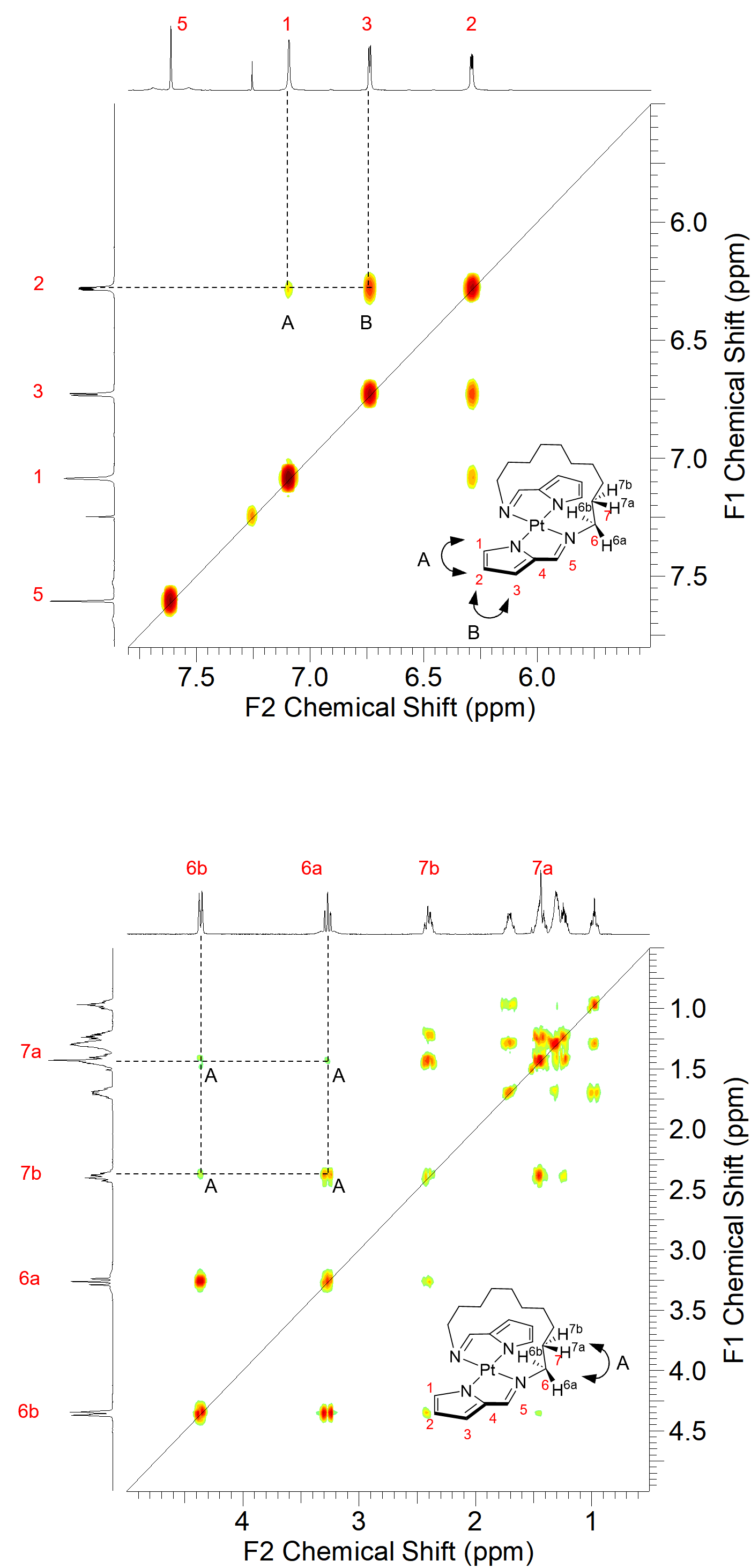


1. COSY spectrum of **1b** in CDCl_3_ (298 K, 500 MHz).


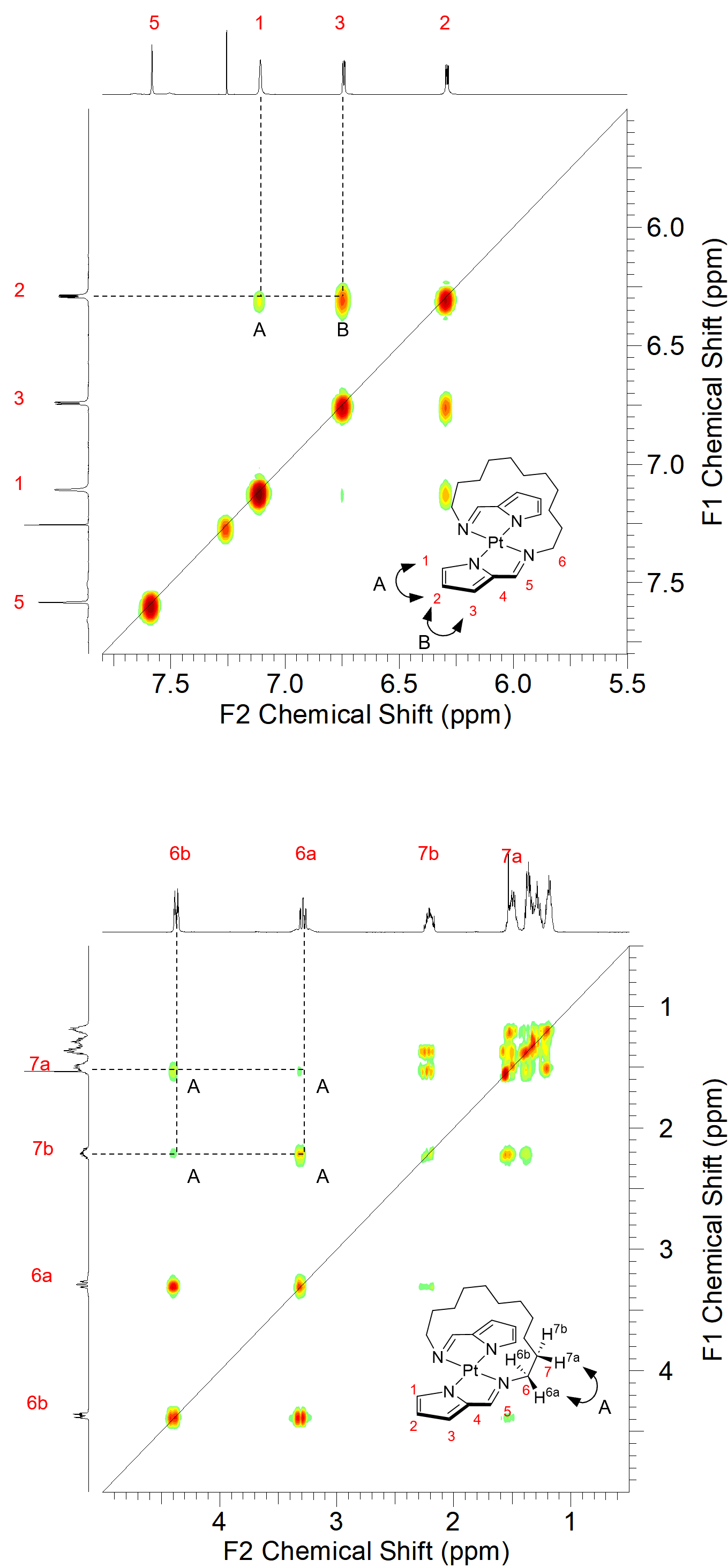


1. COSY spectrum of **1c** in CDCl_3_ (298 K, 500 MHz).


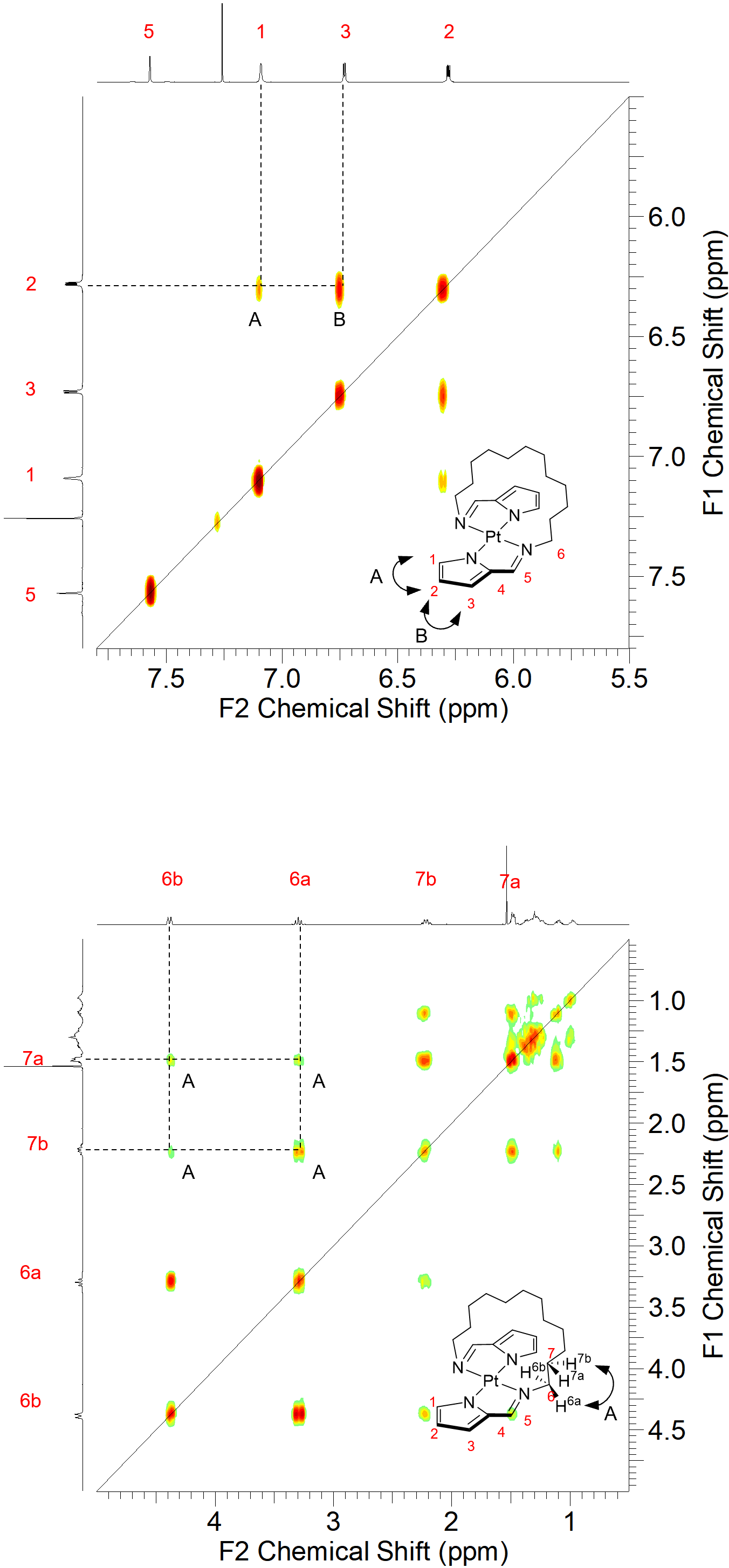


1. COSY spectrum of **1d** in CDCl_3_ (298 K, 500 MHz).

**
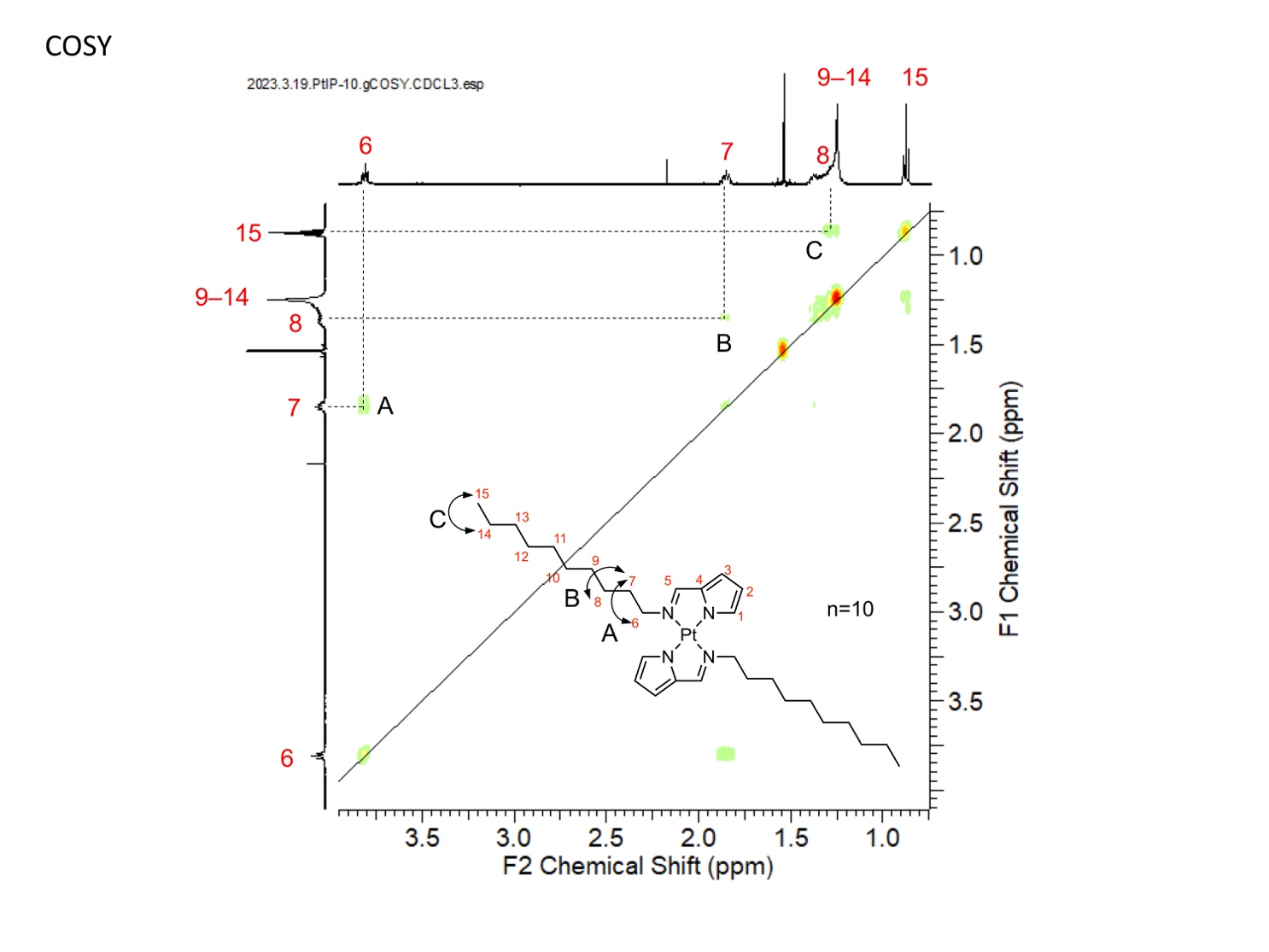
**

**
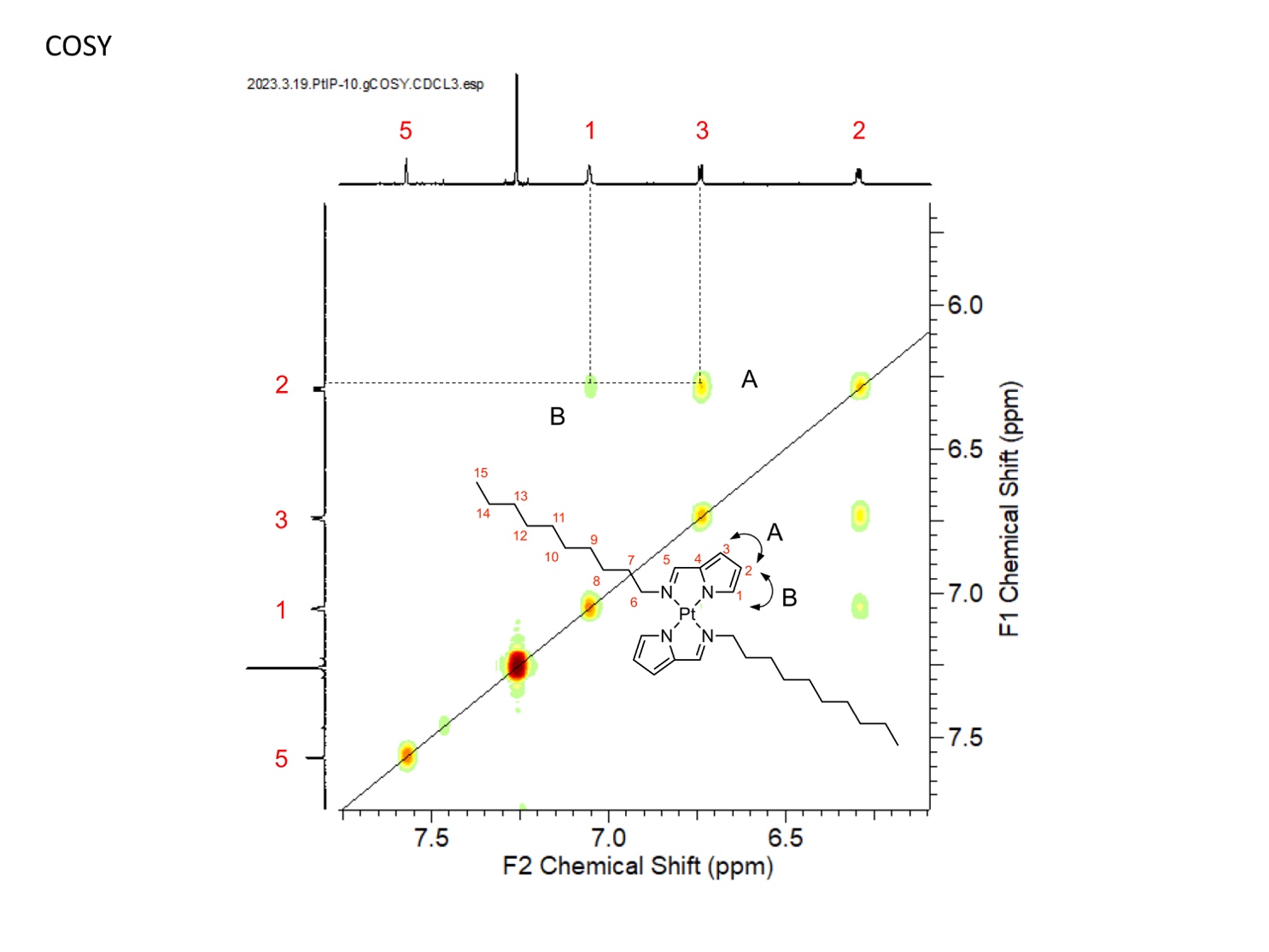
**

1. COSY spectrum of **2** in CDCl_3_ (298 K, 500 MHz).

.


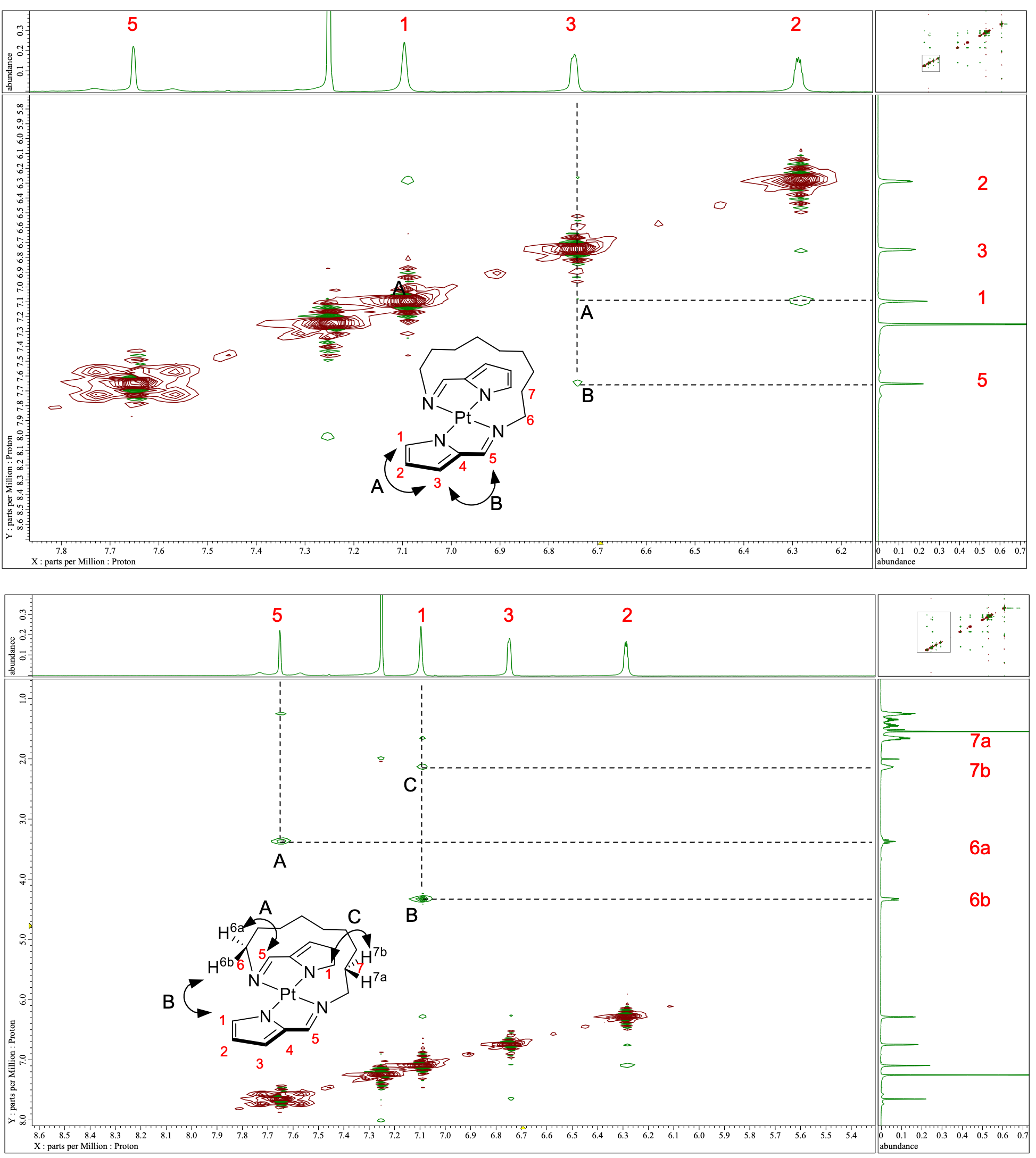


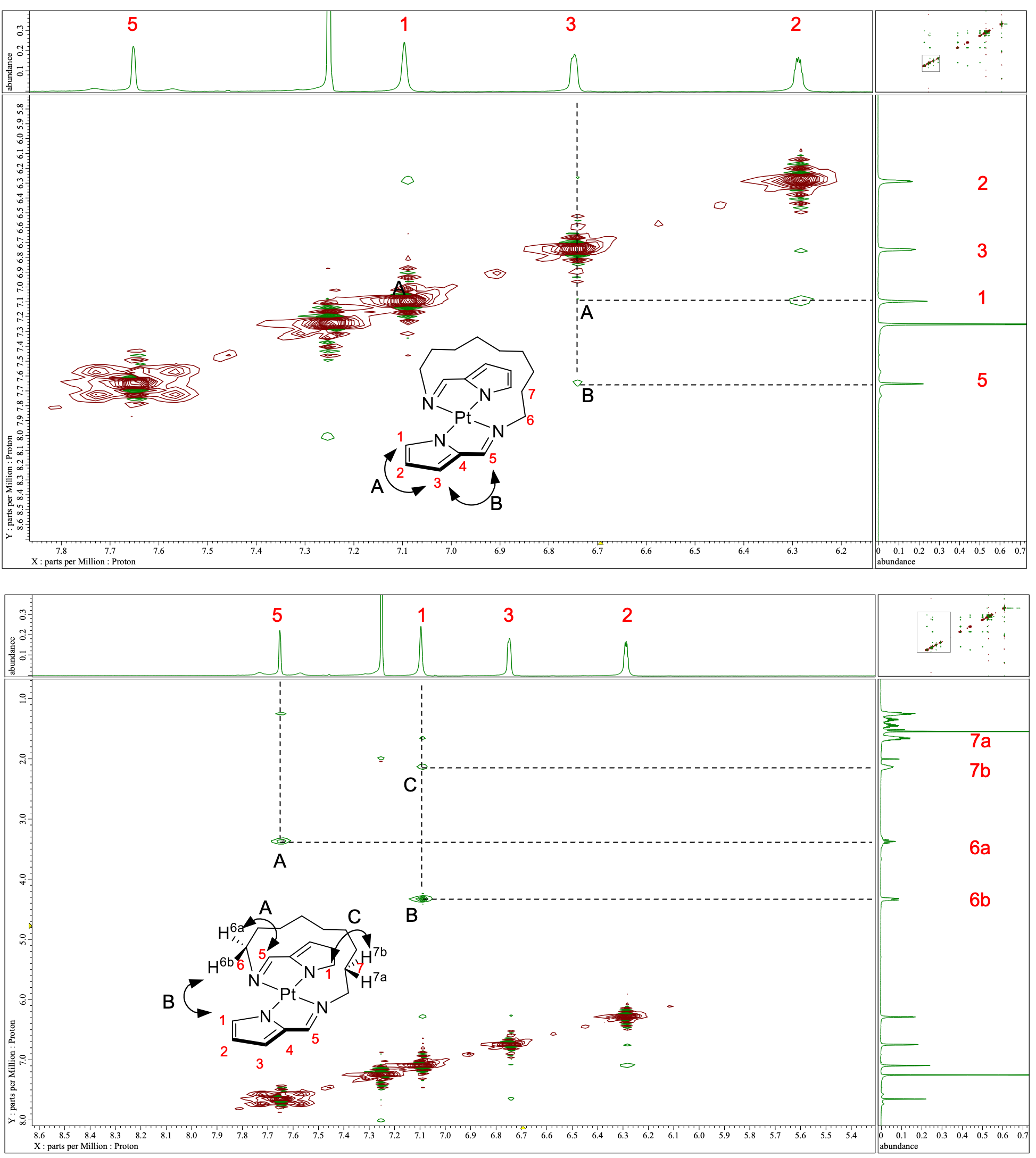


1. NOESY spectrum of **1a** in CDCl_3_ (298 K, 500 MHz)


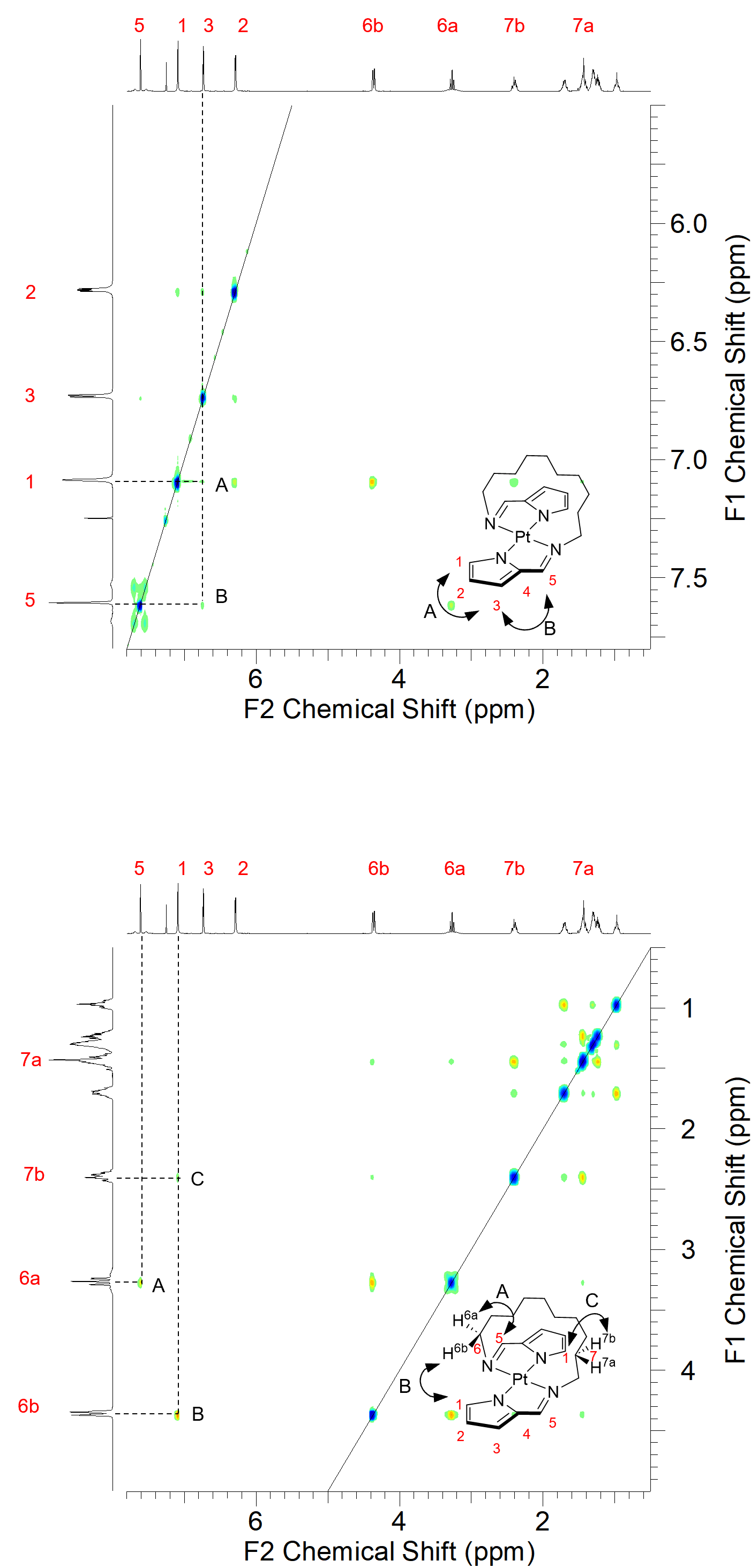


1. NOESY spectrum of **1b** in CDCl_3_ (298 K, 500 MHz)


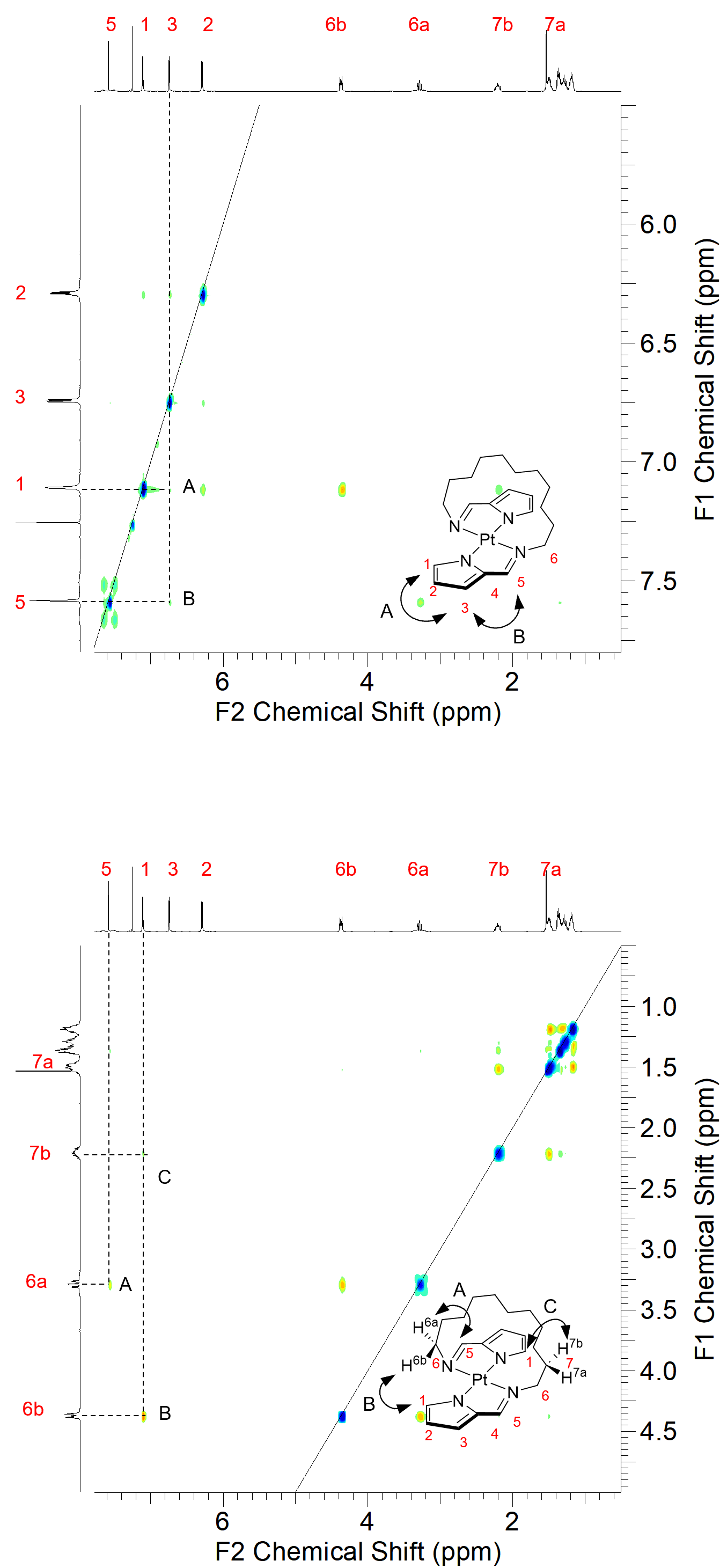


1. NOESY spectrum of **1c** in CDCl_3_ (298 K, 500 MHz)


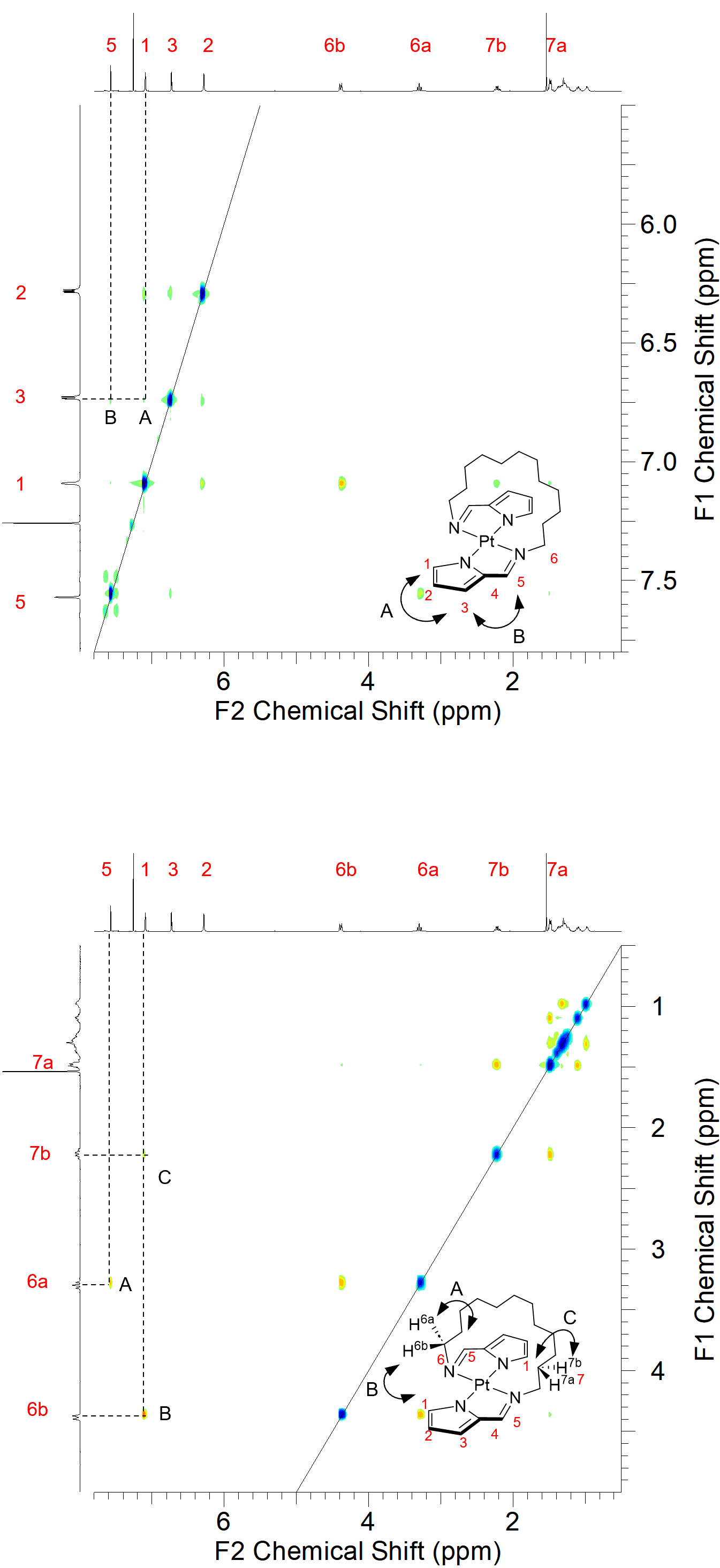


1. NOESY spectrum of **1d** in CDCl_3_ (298 K, 500 MHz)

**
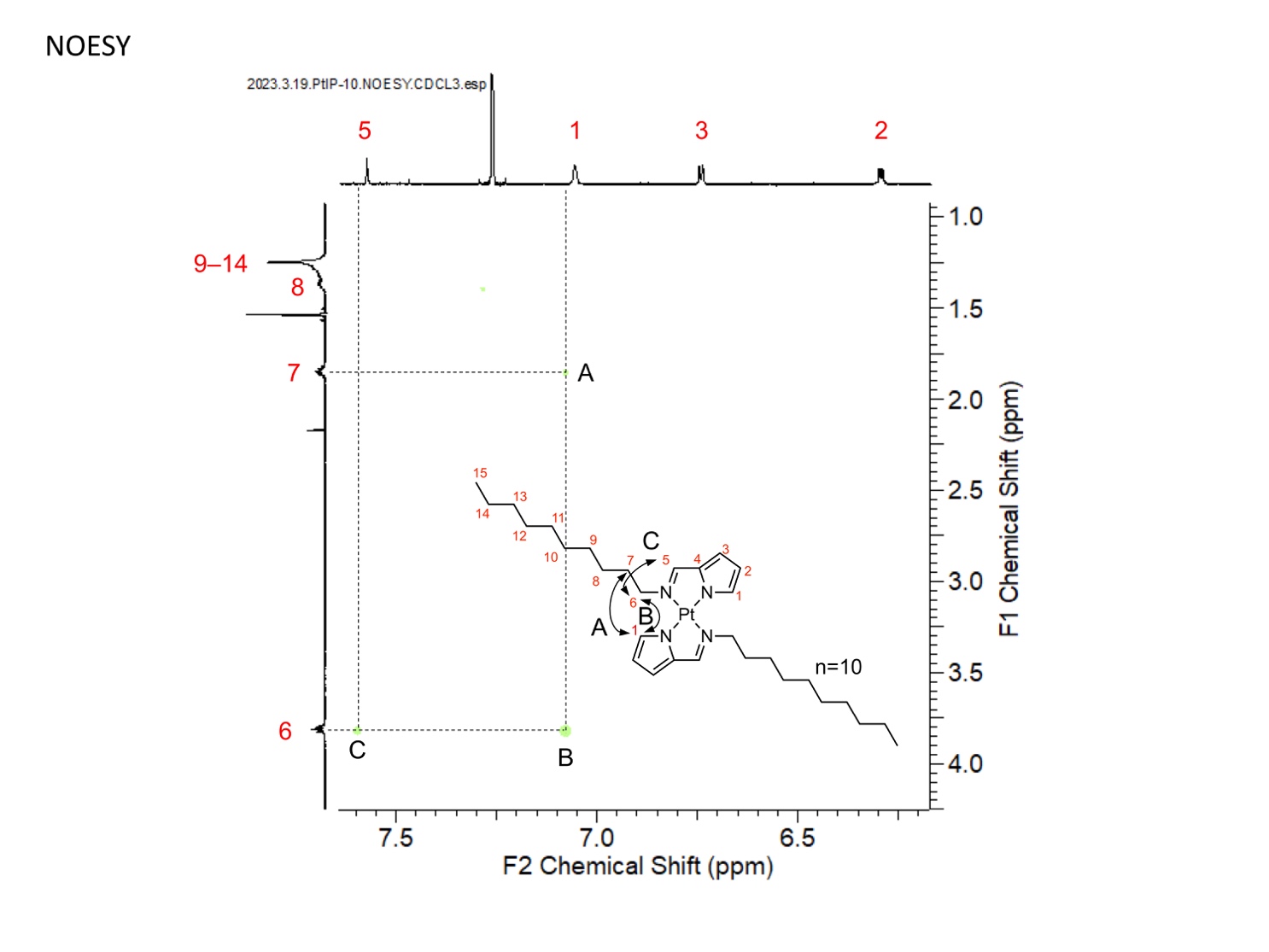
**

**
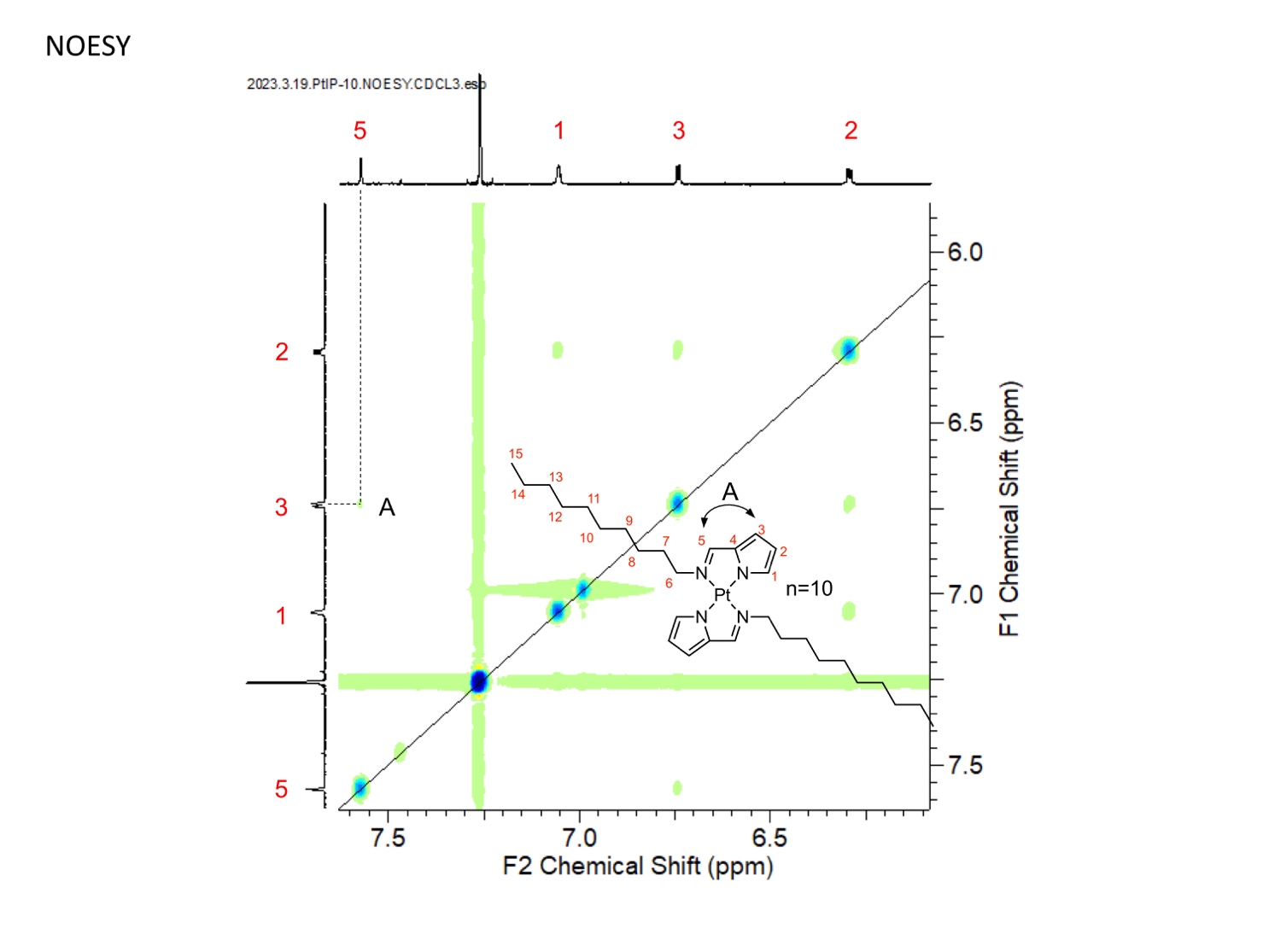
**

1. NOESY spectrum of **2** in CDCl_3_ (298 K, 500 MHz)

**
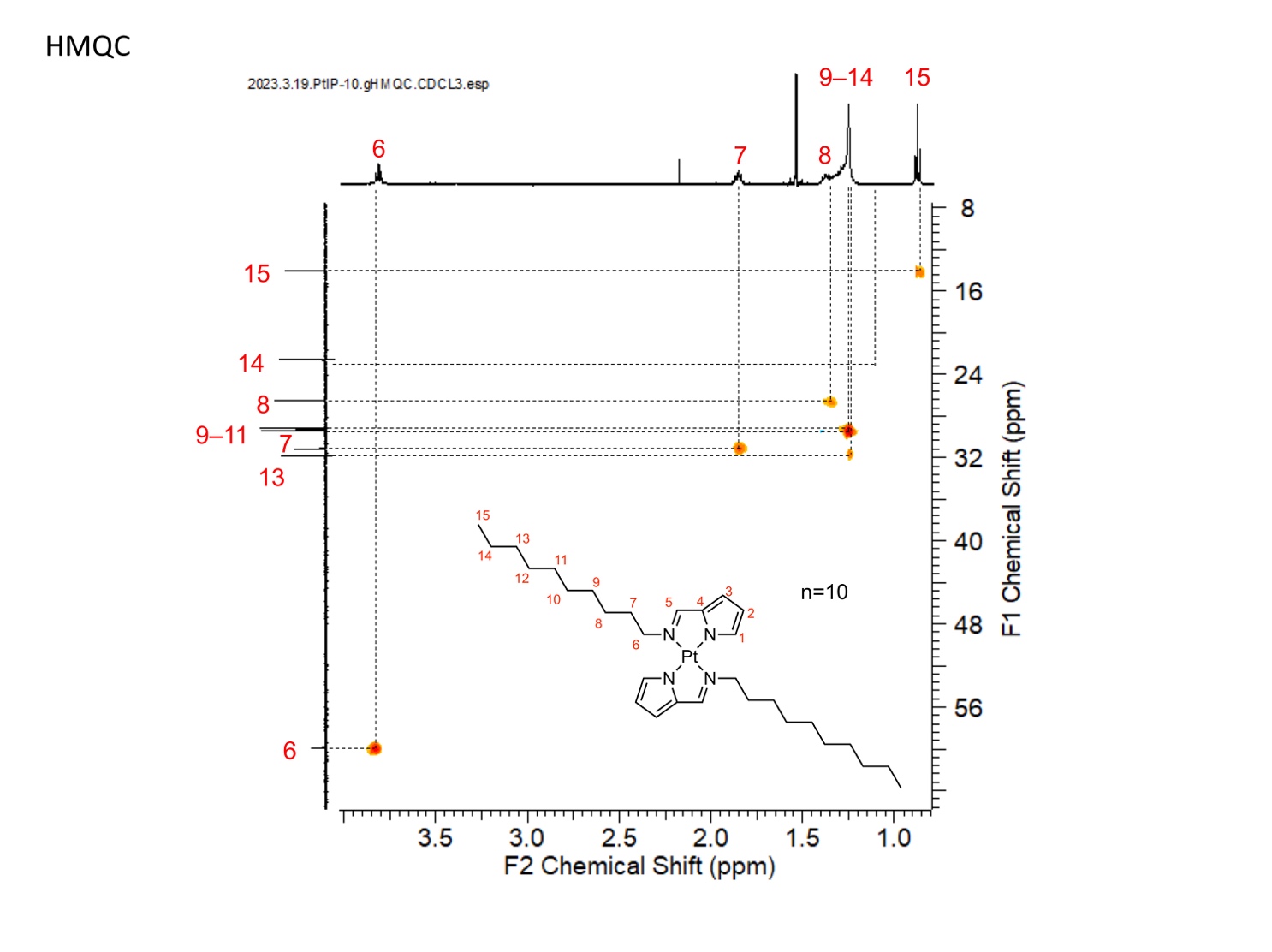
**

**
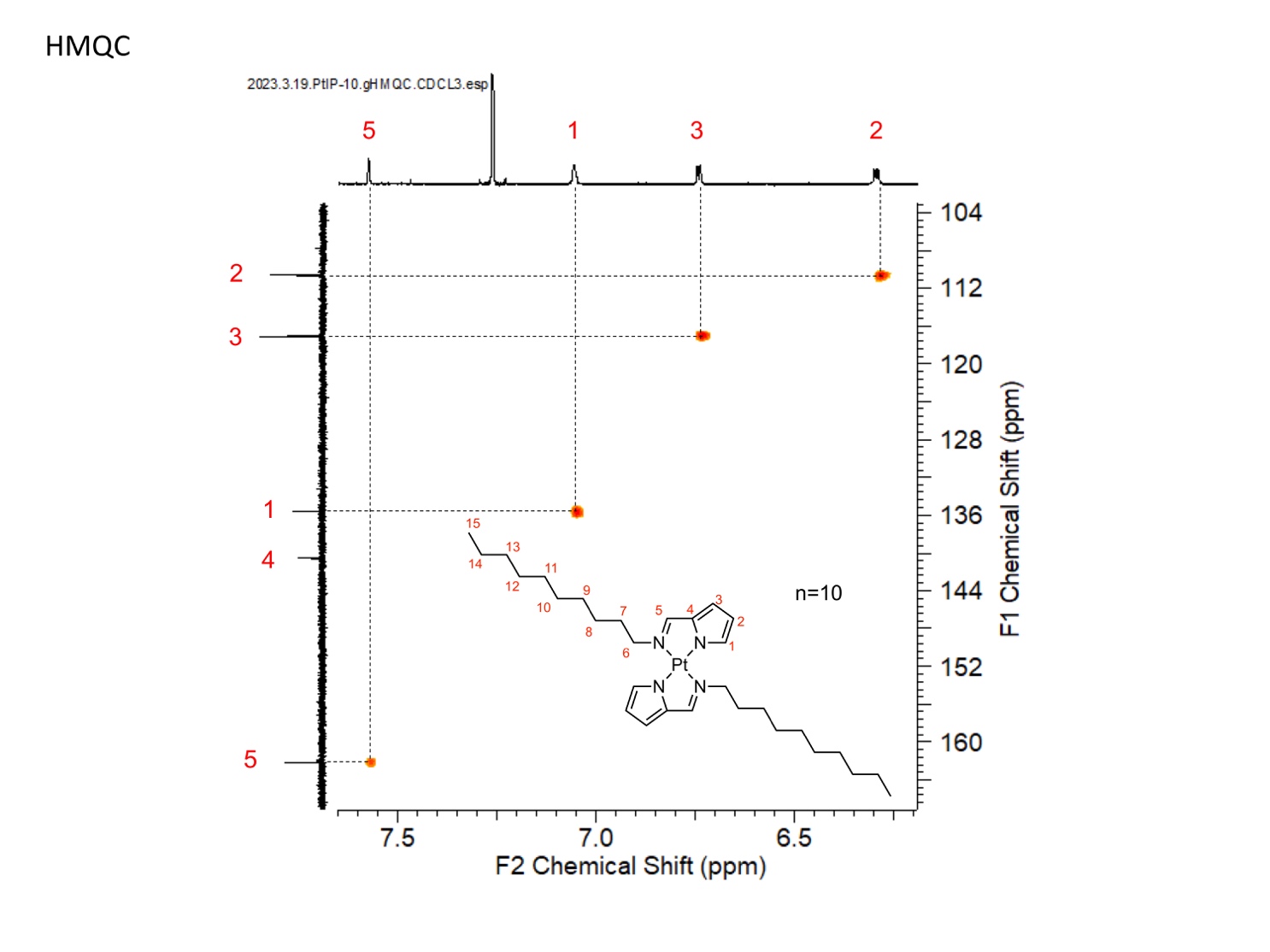
**

1. HMQC spectrum of **2** in CDCl_3_ (298 K, 500 MHz)

1. UV–vis absorption spectra of *rac*-**1a**–**d** and **2** in 2-MeTHF (1.0 × 10⁻⁴ M) at room temperature.
2. Solid-state UV–vis absorption spectra of complexes *rac*-**1a**–**d** and **2** recorded using an integrating sphere (Kubelka–Munk function, F(*R*)) at room temperature, measured as KBr-dispersed samples.
3. Solid-state UV–vis absorption spectra of *rac*-**1d** crystals obtained from acetonitrile recrystallization at 5 °C, 25 °C, and 60 °C, recorded using an integrating sphere (Kubelka–Munk function F(R)) at room temperature, measured as KBr-dispersed samples.
4. Solid-state UV–vis absorption spectra of **2** crystals obtained from acetonitrile recrystallization at 5 °C, 25 °C, and 60 °C, recorded using an integrating sphere (Kubelka–Munk function F(R)) at room temperature, measured as KBr-dispersed samples.
5. Normalized excitation spectra of *rac*-**1d** crystals obtained by recrystallization from acetonitrile at 5 °C, 25 °C, and 60 °C. Measurements were performed at room temperature and monitoring at 557, 539 and 539 nm, respectively.
6. Normalized excitation spectra of **2** crystals obtained by recrystallization from acetonitrile at 5 °C, 25 °C, and 60 °C. Measurements were performed at room temperature and monitoring at 548, 546 and 547 nm, respectively.
7. Normalized emission spectra of *rac*-**1a**–**d** and **2** in 2-MeTHF (1.0 × 10⁻⁴ M) at 77 K (*λ*_ex_ = 420 nm for *rac*-**1a**–**d**, *λ*_ex_ = 430 nm for **2**).
8. Normalized emission spectra of *rac*-**1a** in 2-MeTHF solution (1.0 × 10^–4^ M) and their crystalline states obtained at the respective temperatures. Measurements were performed at room temperature (*λ*_ex_ = 420 nm).
9. Normalized emission spectra of *rac*-**1b** in 2-MeTHF solution (1.0 × 10^–4^ M) and their crystalline states obtained at the respective temperatures. Measurements were performed at room temperature (*λ*_ex_ = 420 nm).
10. Normalized emission spectra of *rac*-**1c** in 2-MeTHF solution (1.0 × 10^–4^ M) and their crystalline states obtained at the respective temperatures. Measurements were performed at room temperature (*λ*_ex_ = 420 nm).


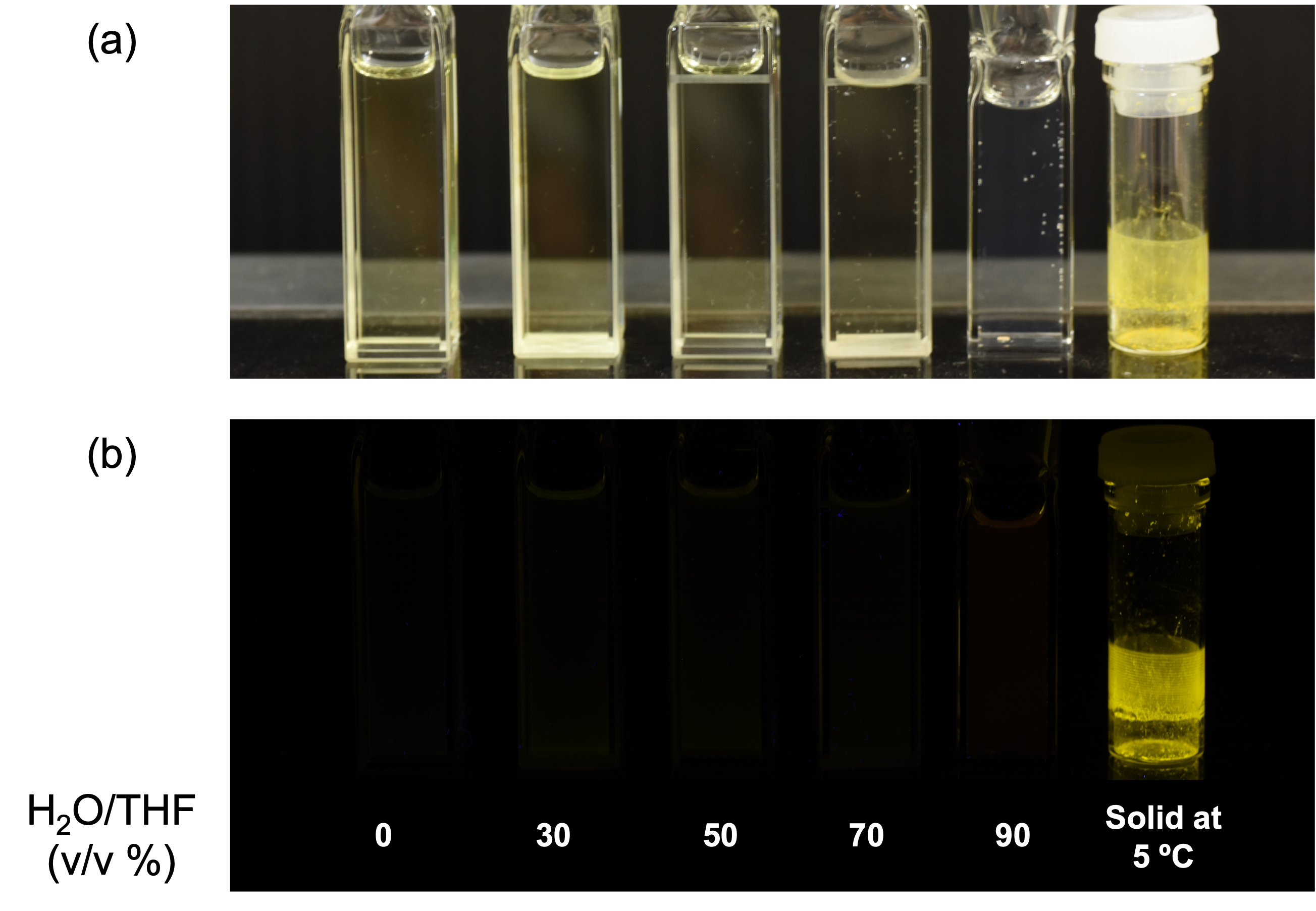


1. Photographs of *rac*-**1d** in H₂O/THF mixed solvents (1.0 × 10⁻⁴ M) with varying water fractions (v/v = 0, 30, 50, 70, and 90%) to evaluate aggregation-induced emission (AIE) properties. Images under (a) ambient light and (b) 365 nm UV irradiation. No emission was observed under any conditions, indicating the absence of AIE behavior in this system. At 90% water content, slight turbidity was observed due to the formation of precipitates. For comparison, a photo of the emissive solid-state sample (recrystallized from acetonitrile at 5 °C) is also included.


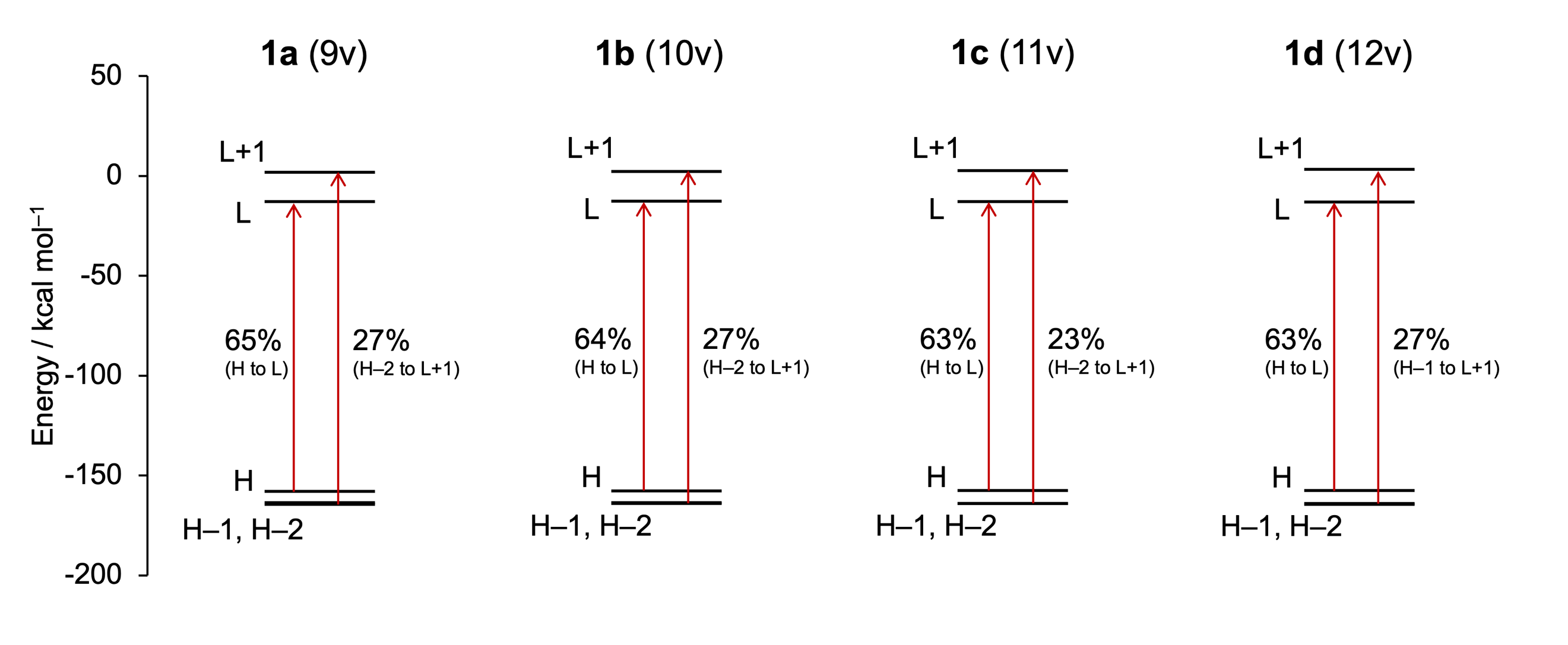


1. Molecular orbital energy diagrams of **1a**–**d**. The red arrows indicate the dominant electronic transitions contributing to the T₁, calculated by TDDFT (CAM-B3LYP/def2-TZVP).


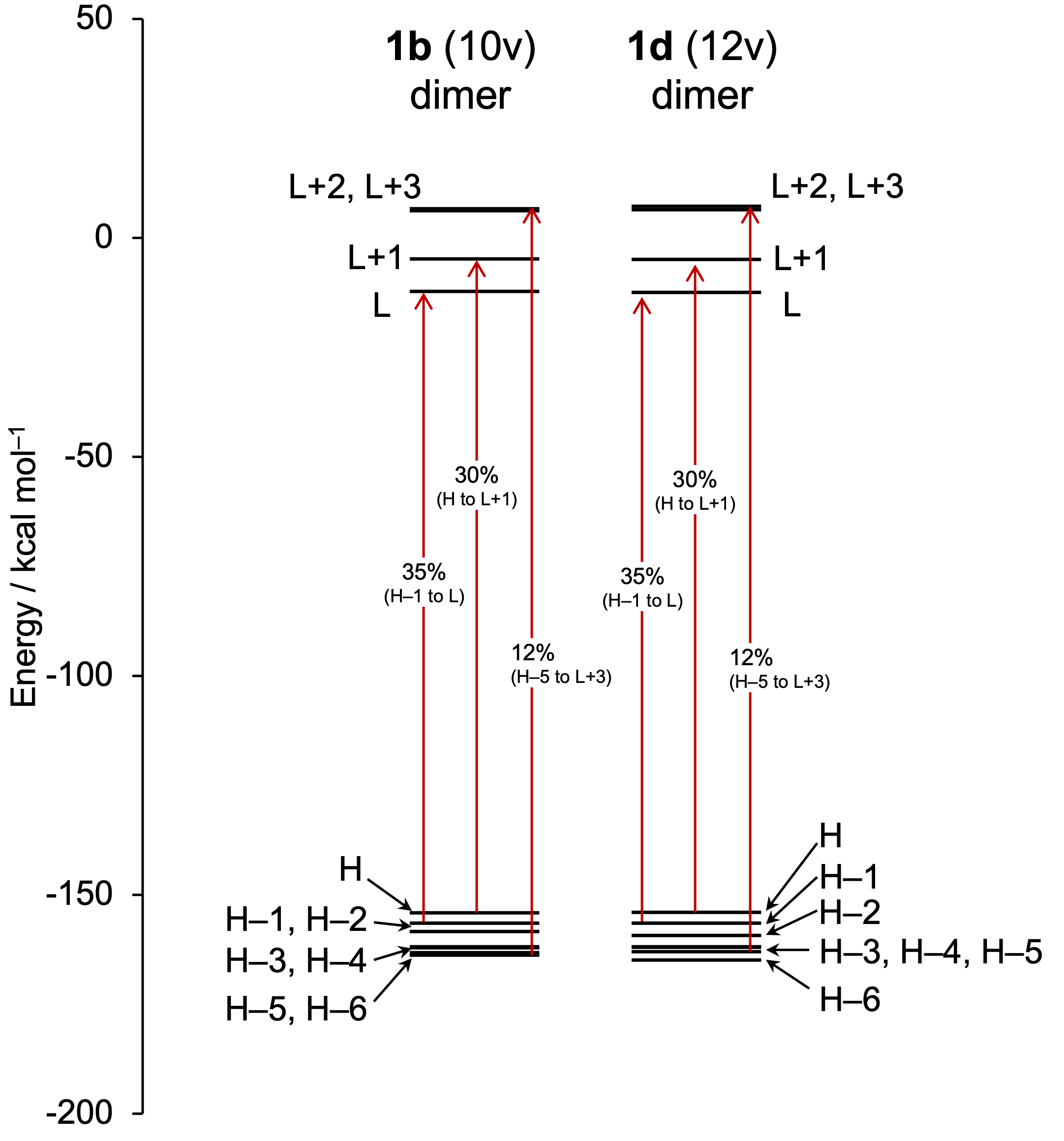


1. Molecular orbital energy diagrams of dimer of **1b** and **d**. The red arrows indicate the dominant electronic transitions contributing to the T₁, calculated by TDDFT (CAM-B3LYP/def2-TZVP).


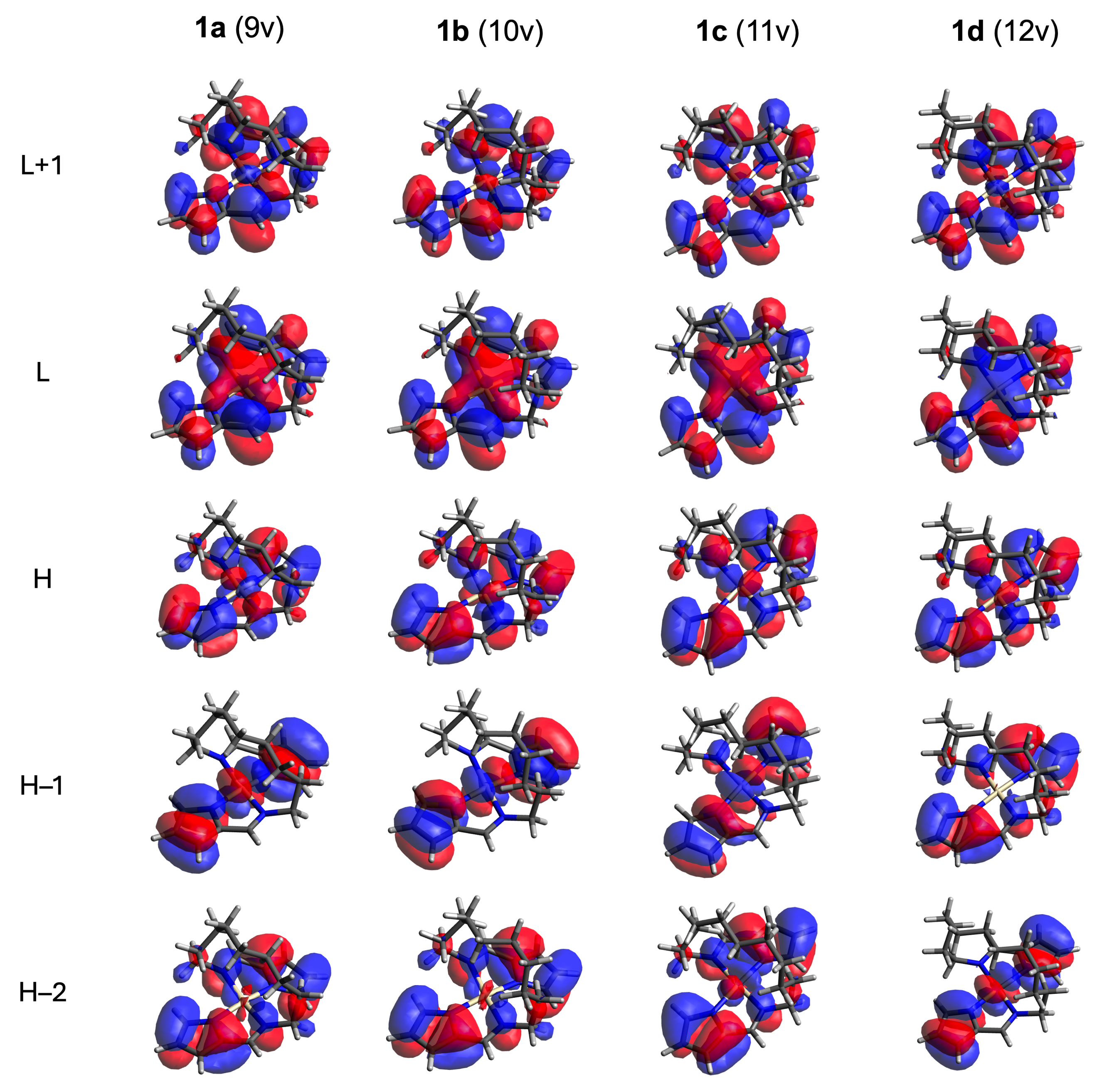


1. Selected molecular orbitals of **1a**–**d** relevant to T₁, visualized based on TDDFT calculations at the CAM-B3LYP/def2-TZVP level.


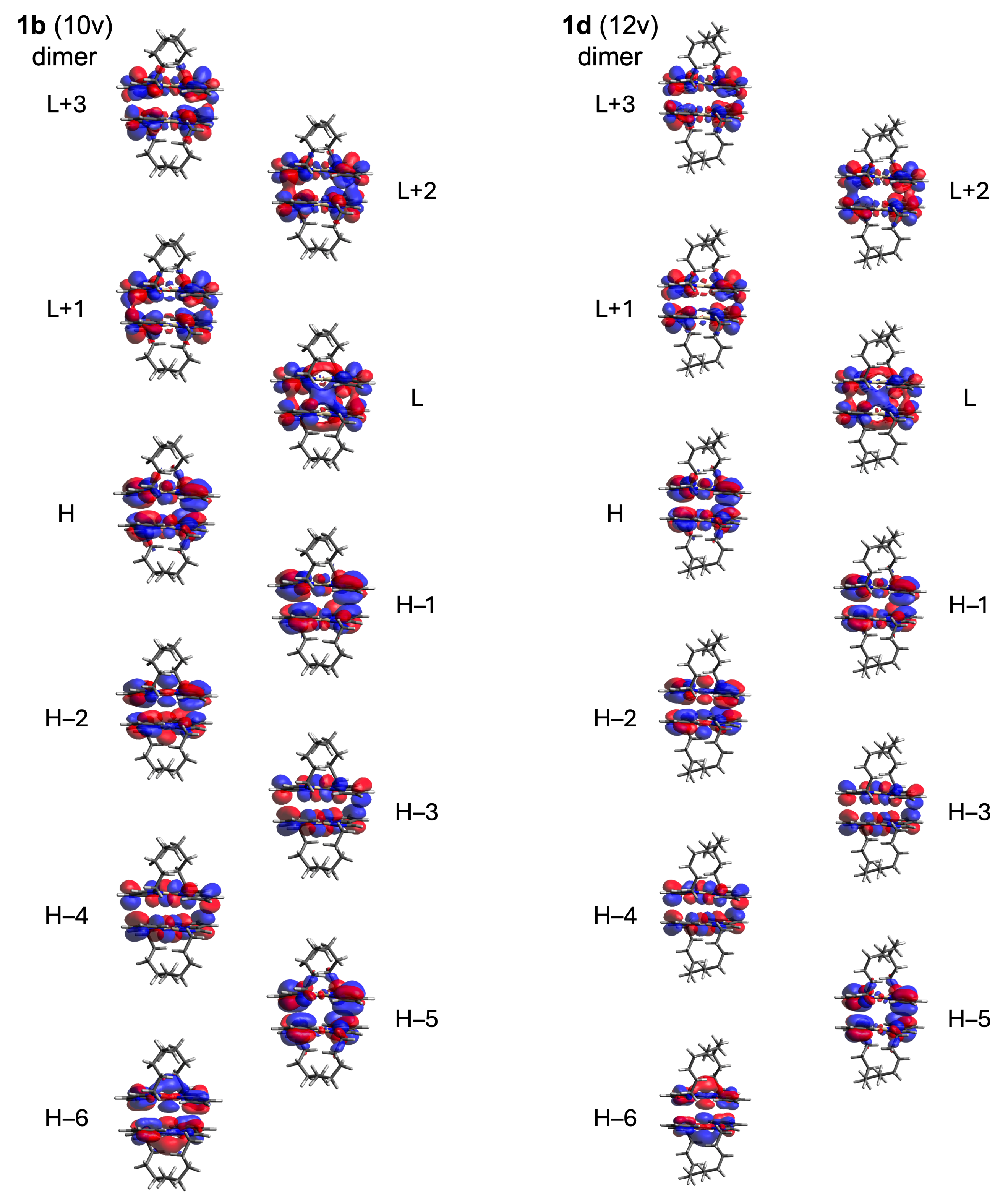


1. Selected molecular orbitals of dimer of **1b** and **d** relevant to T₁, visualized based on TDDFT calculations at the CAM-B3LYP/def2-TZVP level.
2. Calculated energies of selected molecular orbitals for **1a**–**d**.^[a]^

|  | MO energy (kcal/mol) | | | |
| --- | --- | --- | --- | --- |
|  | **1a** | **1b** | **1c** | **1d** |
| LUMO+1 | 2.159 | 2.673 | 3.050 | 3.564 |
| LUMO | –12.50 | –12.40 | –12.47 | –12.82 |
| HOMO | –157.5 | –157.4 | –157.3 | –157.3 |
| HOMO–1 | –163.3 | –163.3 | –163.6 | –163.7 |
| HOMO–2 | –164.0 | –163.7 | –163.7 | –164.0 |

[a] The calculation performed at CAM-B3LYP/def2-TZVP.

1. Calculated energies of selected molecular orbitals for dimer of **1b** and **d**.^[a]^

|  | MO energy (kcal/mol) | |
| --- | --- | --- |
|  | **1b** dimer | **1d** dimer |
| LUMO+3 | 6.846 | 7.405 |
| LUMO+2 | 6.382 | 6.645 |
| LUMO+1 | –4.600 | –4.763 |
| LUMO | –12.07 | –12.27 |
| HOMO | –153.9 | –153.8 |
| HOMO–1 | –156.4 | –156.3 |
| HOMO–2 | –158.2 | –159.1 |
| HOMO–3 | –161.7 | –161.8 |
| HOMO–4 | –162.0 | –161.8 |
| HOMO–5 | –163.0 | –162.8 |
| HOMO–6 | –163.6 | ­–164.8 |

[a] The calculation performed at CAM-B3LYP/def2-TZVP.

1. Photophysical data for *rac*-**1a**–**1d** and **2** in 2-MeTHF (1.0 × 10^–4^ M).^[a]^

| Complex | *λ*_abs_ [nm] | *λ*_em_ [nm]^[b]^ | *Φ*^[b,c]^ | *τ* [μs]^[d]^ | *k*_r_ × 10^–4^ [s^–1^] | *k*_nr_ × 10^–4^ [s^–1^] |
| --- | --- | --- | --- | --- | --- | --- |
| *rac*-**1a** | 378, 410, 439 | -  (534, 579, 617) | <0.005  (0.70) | -  (34.8) | -  (2.01) | -  (0.86) |
| *rac*-**1b** | 376, 418, 447 | -  (534, 580, 616) | <0.005  (0.61) | -  (32.0) | -  (1.91) | -  (1.22) |
| *rac*-**1c** | 375, 421, 448 | -  (531, 576, 618) | <0.005  (0.60) | -  (32.7) | -  (1.83) | -  (1.22) |
| *rac*-**1d** | 376, 422, 446 | 542, 582  (532, 578, 619) | <0.005  (0.63) | 0.15  (38.6) | -  (1.63) | -  (0.96) |
| **2** | 375, 422, 446 | 547, 587  (534, 589) | <0.005  (0.58) | 1.09  (30.3) | -  (1.92) | -  (1.39) |

[a] Data are measured at 298 K and 77 K (in parentheses). [b] *λ*_ex_: 420 nm for *rac*-**1a**–**1d** and 430 nm for **2**. [c] Determined by the absolute method using an integrating sphere. [d] *λ*_ex_: 355 nm.

1. Photophysical data of *rac*-**1a**–**1d** and **2** in crystalline state.^[a]^

| Complex | Recrystallization temp. | *λ*_em_ [nm]^[b]^ | *Φ*^[b,c]^ | *τ* [μs]^[d]^ | *k*_r_ × 10^–4^ [s^–1^] | *k*_nr_ × 10^–4^ [s^–1^] |
| --- | --- | --- | --- | --- | --- | --- |
| *rac*-**1a** | 5 ^o^C (278 K) | 569 | 0.005 | 20.73 | 0.024 | 4.80 |
| *rac*-**1b** | 5 ^o^C (278 K) | 564 | 0.24 | 9.30 | 2.58 | 8.18 |
| *rac*-**1c** | 5 ^o^C (278 K) | 558 | 0.01 | 10.82 | 0.09 | 9.15 |
| *rac*-**1d** | 5 ^o^C (278 K) | 557  (555, 607) | 0.33  (0.66) | 2.55  (8.85) | 13.0  (7.46) | 26.3  (3.84) |
| *rac*-**1a** | 25 ^o^C (278 K) | 566 | 0.02 | - | - | - |
| *rac*-**1b** | 25 ^o^C (278 K) | 564 | 0.20 | 16.98 | 1.18 | 4.71 |
| *rac*-**1c** | 25 ^o^C (278 K) | 556 | 0.01 | - | - | - |
| *rac*-**1d** | 25 ^o^C (278 K) | 539  (542) | 0.02  (0.14) | 0.07  (0.22) | 29.4  (64.1) | 1440  (394) |
| *rac*-**1a** | 60 ^o^C (333 K) | 579 | <0.005 | - | - | - |
| *rac*-**1b** | 60 ^o^C (333 K) | 589 | 0.009 | - | - | - |
| *rac*-**1c** | 60 ^o^C (333 K) | 556 | 0.01 | - | - | - |
| *rac*-**1d** | 60 ^o^C (333 K) | 539 | <0.005 | - | - | - |
| (*S*)-**1a** | 5 ^o^C (278 K) | 579 | <0.005 | - | - | - |
| (*S*)-**1b** | 5 ^o^C (278 K) | 539 | <0.005 | - | - | - |
| (*S*)-**1c** | 5 ^o^C (278 K) | 541 | 0.01 | - | - | - |
| (*S*)-**1d** | 5 ^o^C (278 K) | 537 | <0.005 | - | - | - |
| (*R*)-**1a** | 5 ^o^C (278 K) | 572 | 0.02 | - | - | - |
| (*R*)-**1b** | 5 ^o^C (278 K) | 540 | 0.01 | - | - | - |
| (*R*)-**1c** | 5 ^o^C (278 K) | 539 | 0.02 | - | - | - |
| (*R*)-**1d** | 5 ^o^C (278 K) | 536 | <0.005 | - | - | - |
| **2** | 5 ^o^C (278 K) | 548 | <0.005 | - | - | - |
| **2** | 25 ^o^C (298 K) | 546 | <0.005 | - | - | - |
| **2** | 60 ^o^C (333 K) | 547 | <0.005 | - | - | - |

[a] Data were obtained at room temperature and (–196 ℃, 77 K, in parentheses). [b] *λ*_ex_: 420 nm for **1a**–**1d** and 430 nm for **2**. [c] Determined by the absolute method using an integrating sphere. [d] *λ*_ex_: 355 nm.


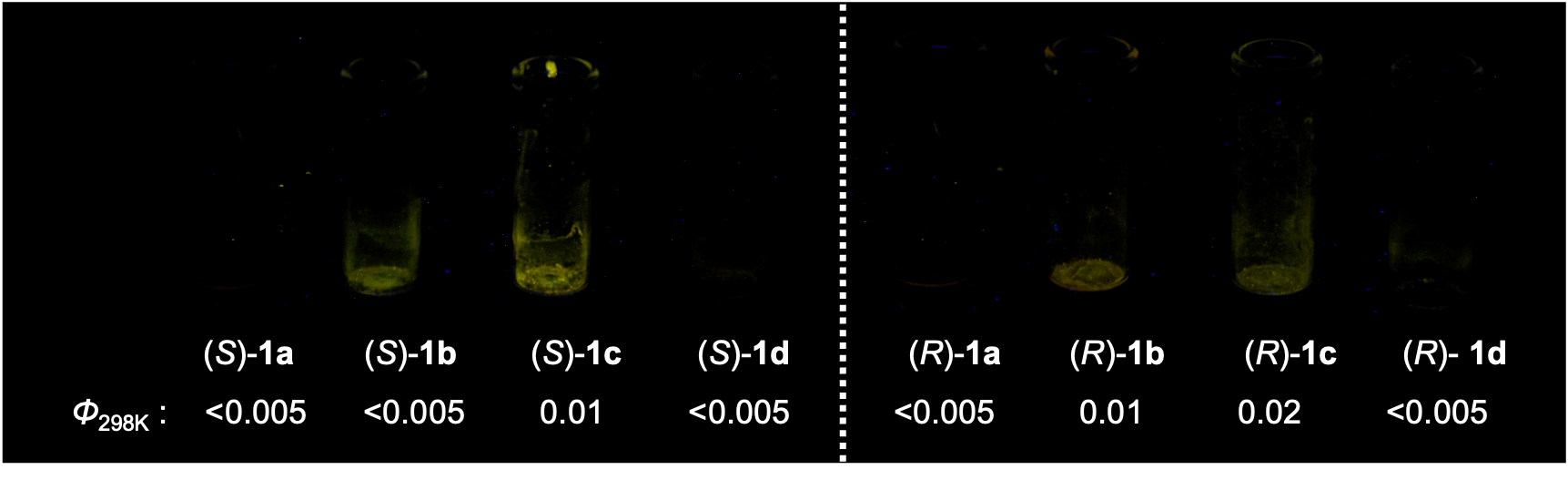


1. Photographs of (*S*) and (*R*)-**1a**–**d** crystals (recrystallized from acetonitrile at 5 ℃ under UV irradiation (365 nm).


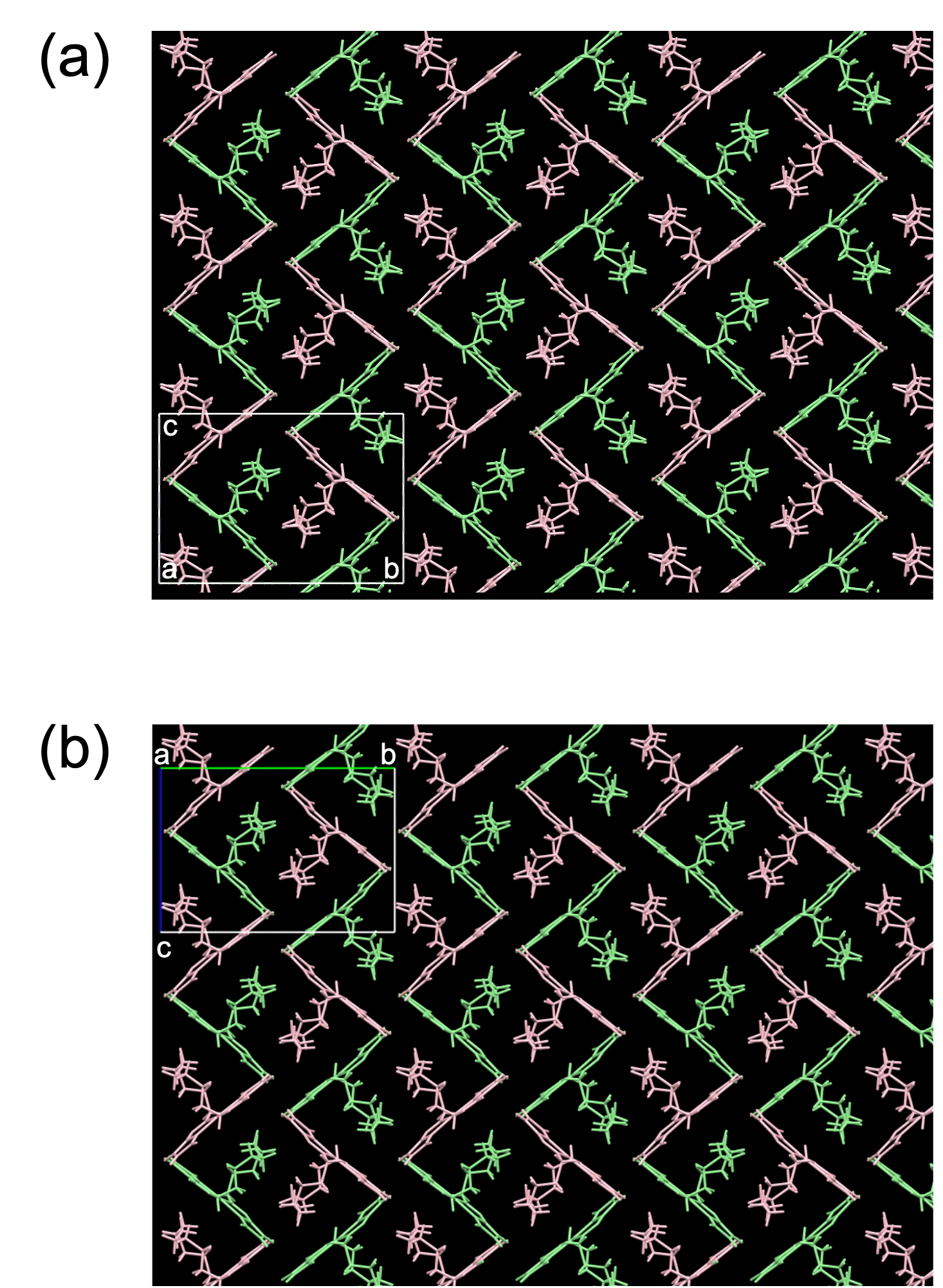


1. Crystal packing structure of *rac*-**1d** obtained from acetonitrile at (a) 25 and (b) 60 °C, as determined by single-crystal X-ray analysis. The pink and green molecules represent the *S-* and *R-*enantiomers, respectively.


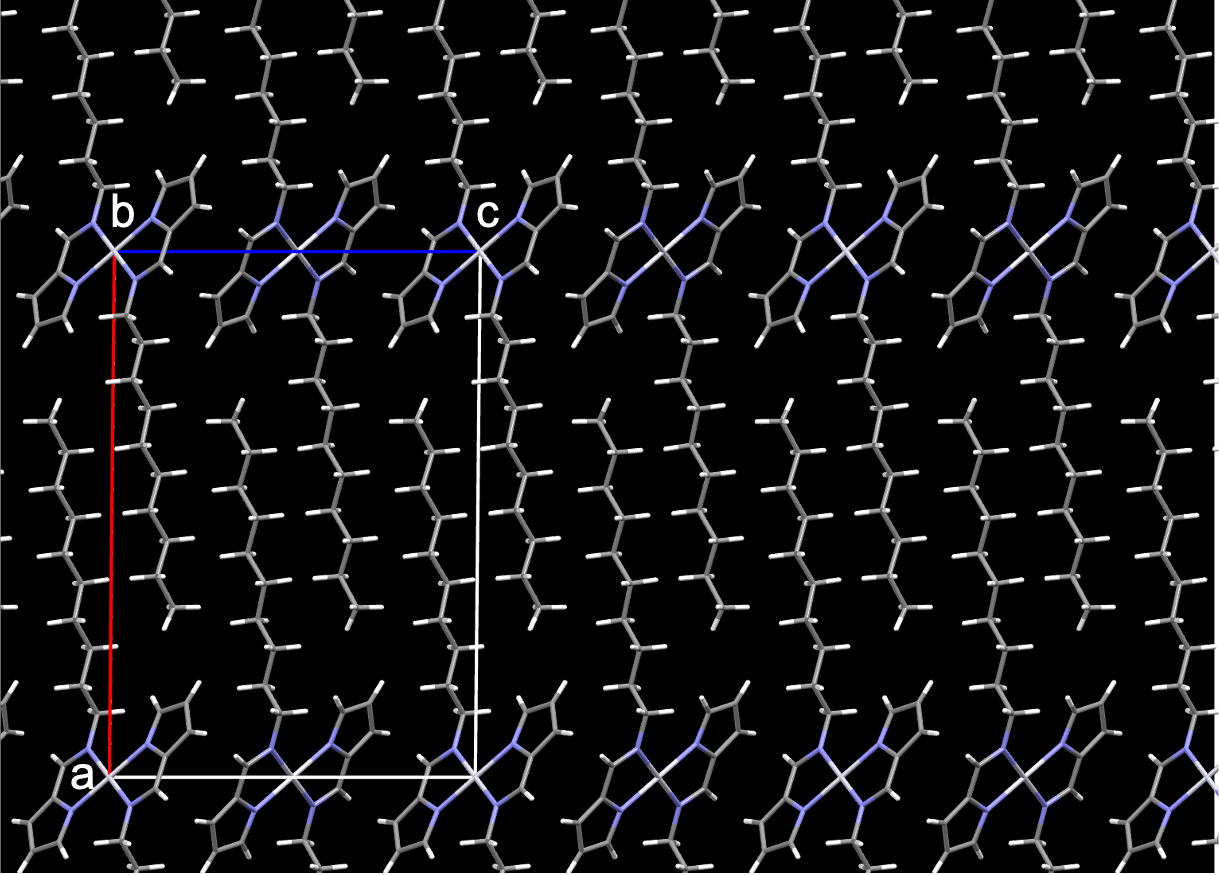


1. Crystal packing structure of **2** obtained from acetonitrile at 5 °C, as determined by single-crystal X-ray analysis.


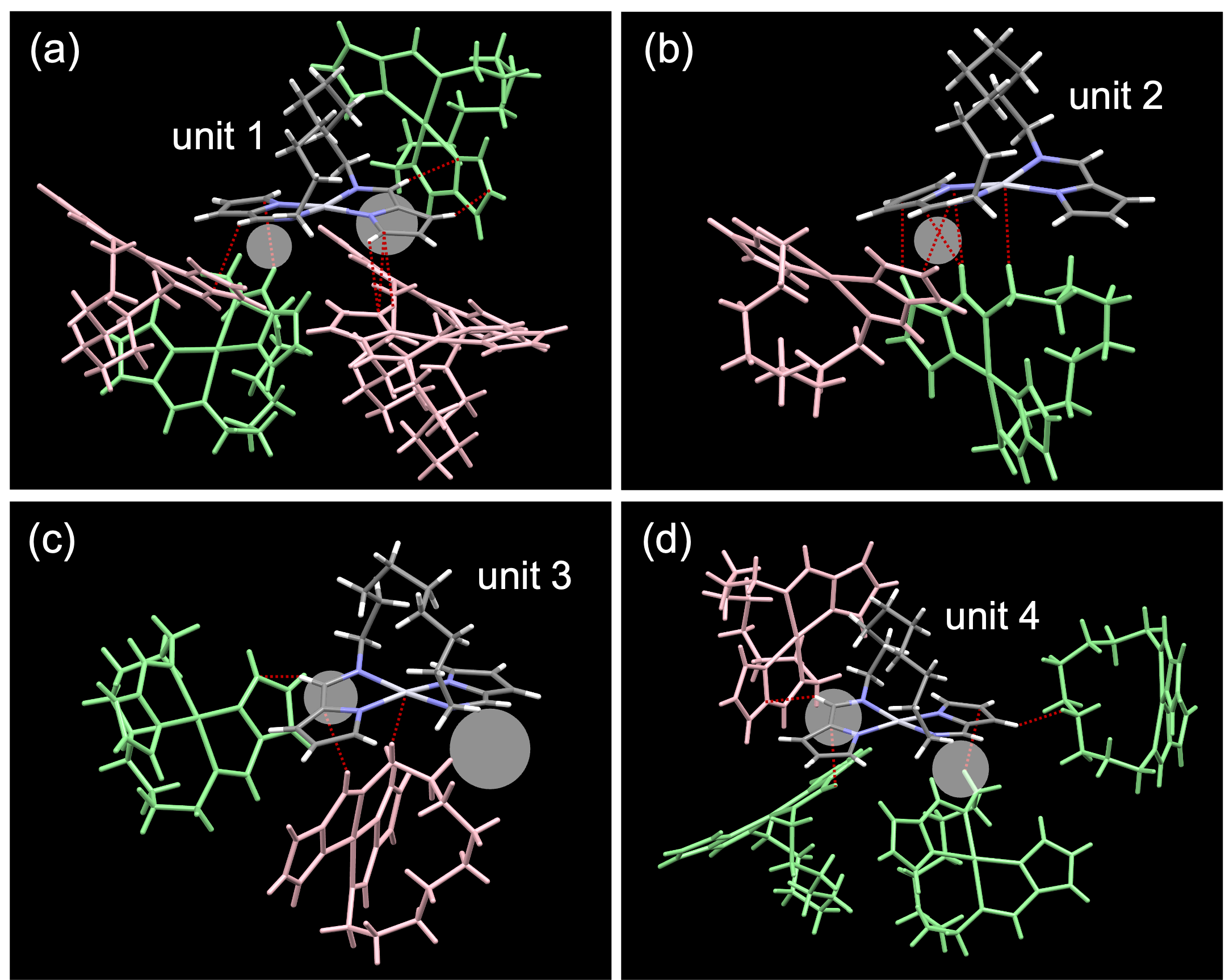


1. Intermolecular interactions (red dotted lines) observed around the square-planar Pt(II) planes in the crystal packing of *rac*-**1a**. The structures shown in (a–d) correspond to the four crystallographically independent molecules in the asymmetric unit. The gray translucent circles highlight void spaces beneath the imine moieties, suggesting that these regions can accommodate downward bending.


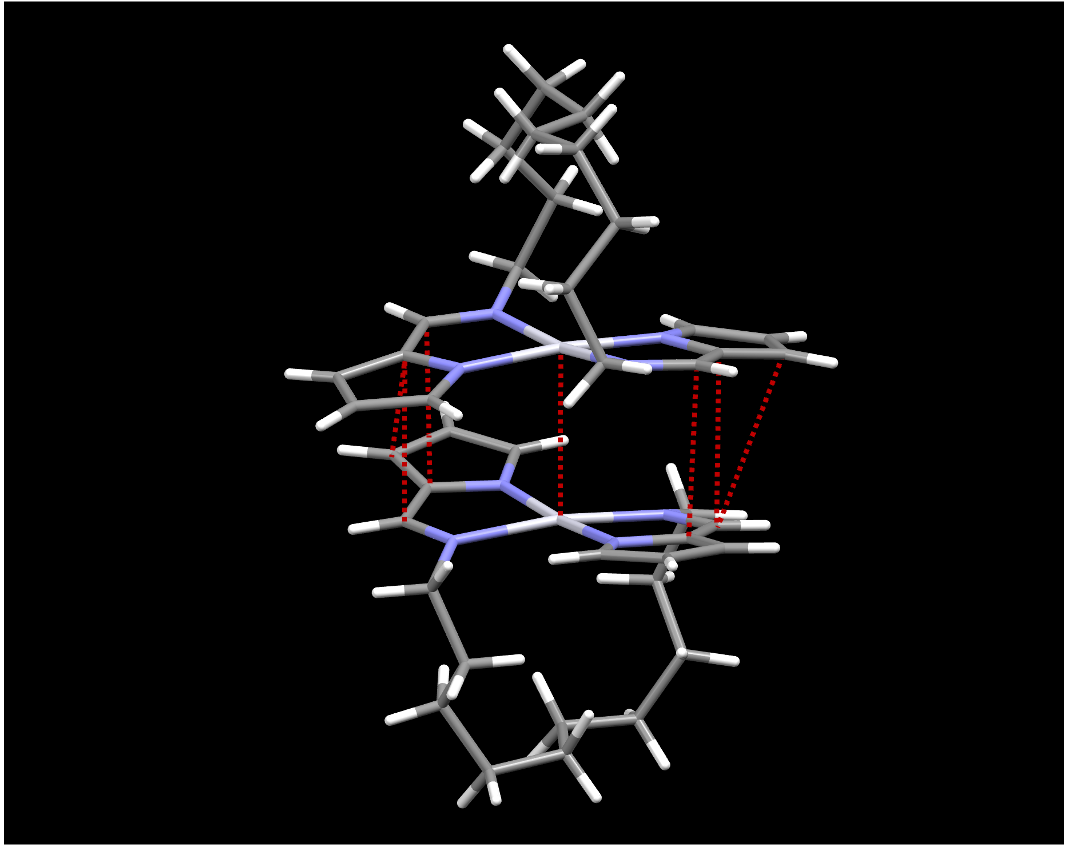


1. Intermolecular interactions (red dotted lines) observed around the square-planar Pt(II) planes in the crystal packing of *rac*-**1b**.

**
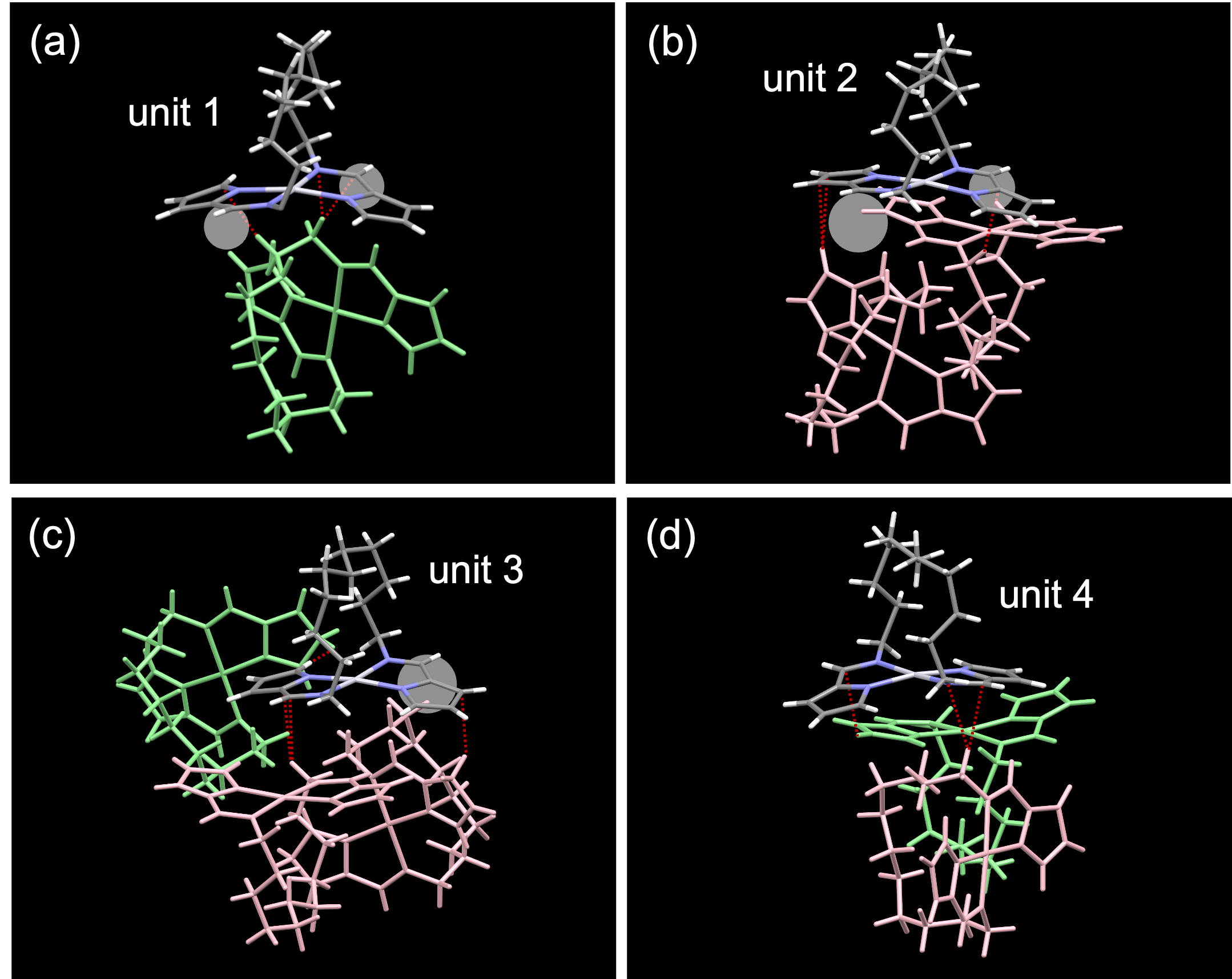
**

1. Intermolecular interactions (red dotted lines) observed around the square-planar Pt(II) planes in the crystal packing of *rac*-**1c**. The structures shown in (a–d) correspond to the four crystallographically independent molecules in the asymmetric unit. The gray translucent circles highlight void spaces beneath the imine moieties, suggesting that these regions can accommodate downward bending.

**
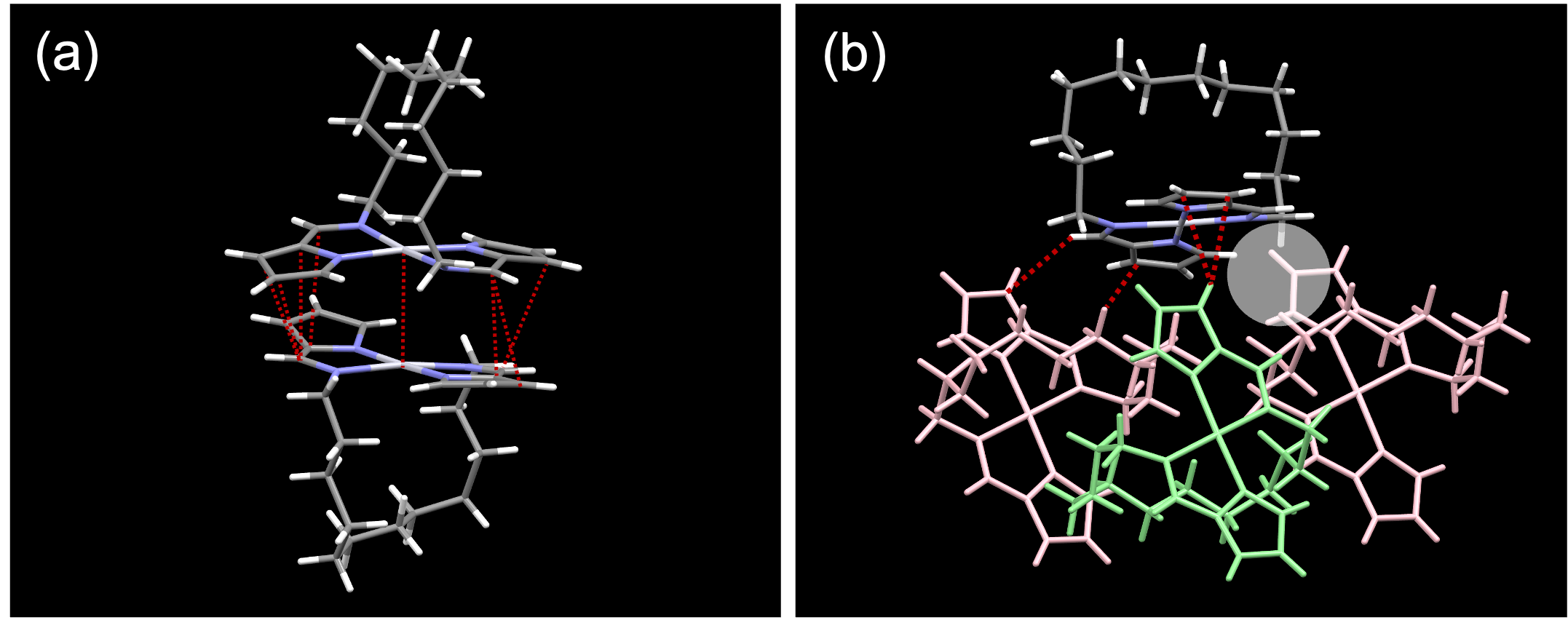
**

1. Intermolecular interactions (red dotted lines) observed around the square-planar Pt(II) planes in the crystal packing of *rac*-**1d**. The structures shown in (a) and (b) were obtained by recrystallization from acetonitrile at 5 °C and 25 °C, respectively. The gray translucent circles highlight void spaces beneath the imine moieties, suggesting that these regions can accommodate downward bending.


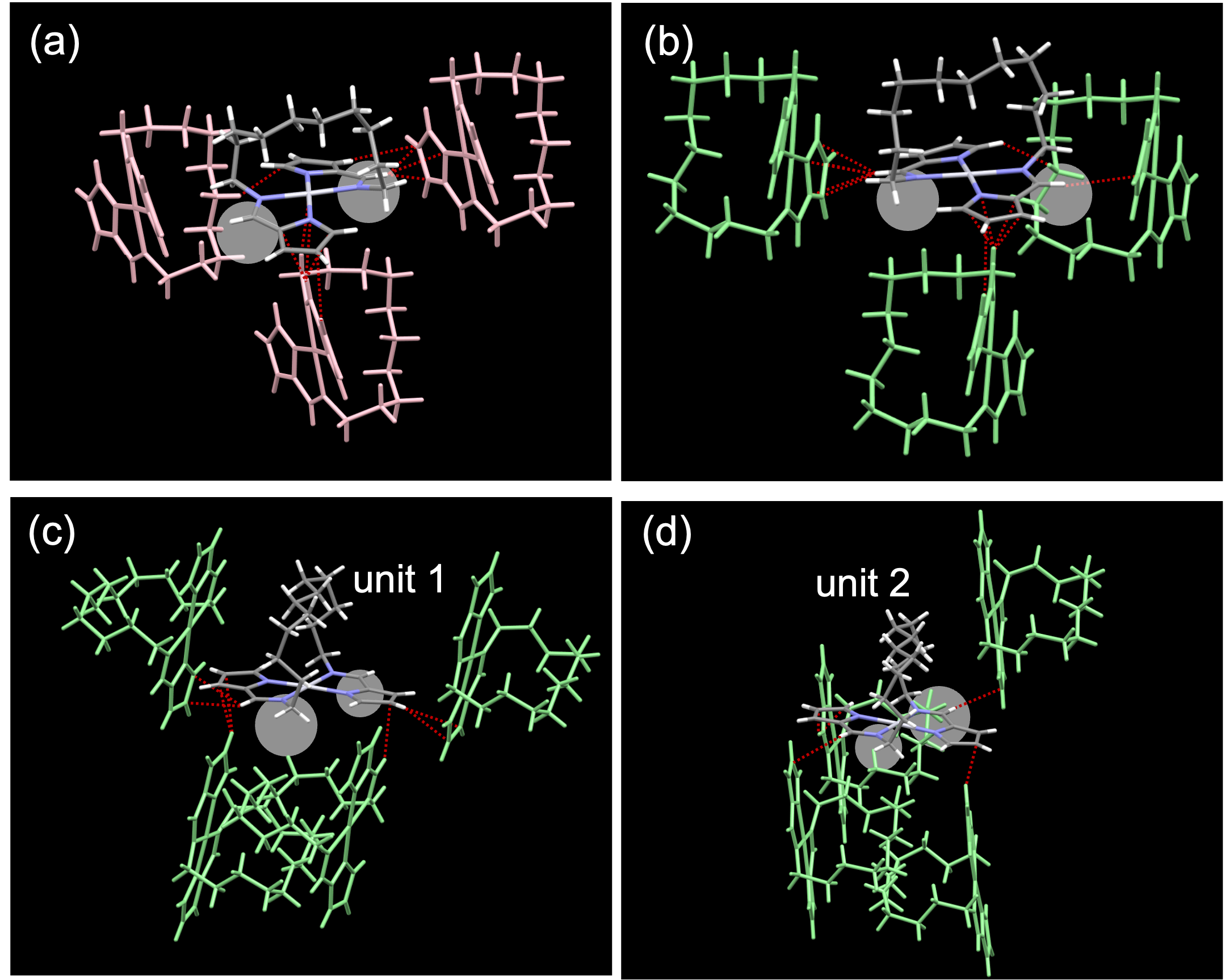


1. Intermolecular interactions (red dotted lines) observed around the square-planar Pt(II) planes in the crystal packing of (a) (*S*)-**1b**, (b) (*R*)-**1c**, and (c,d) (*R*)-**1d**. The structures shown in (c) and (d) correspond to the two crystallographically independent molecules of **1d** in the asymmetric unit. The gray translucent circles highlight void spaces beneath the imine moieties, suggesting that these regions can accommodate downward bending.

**
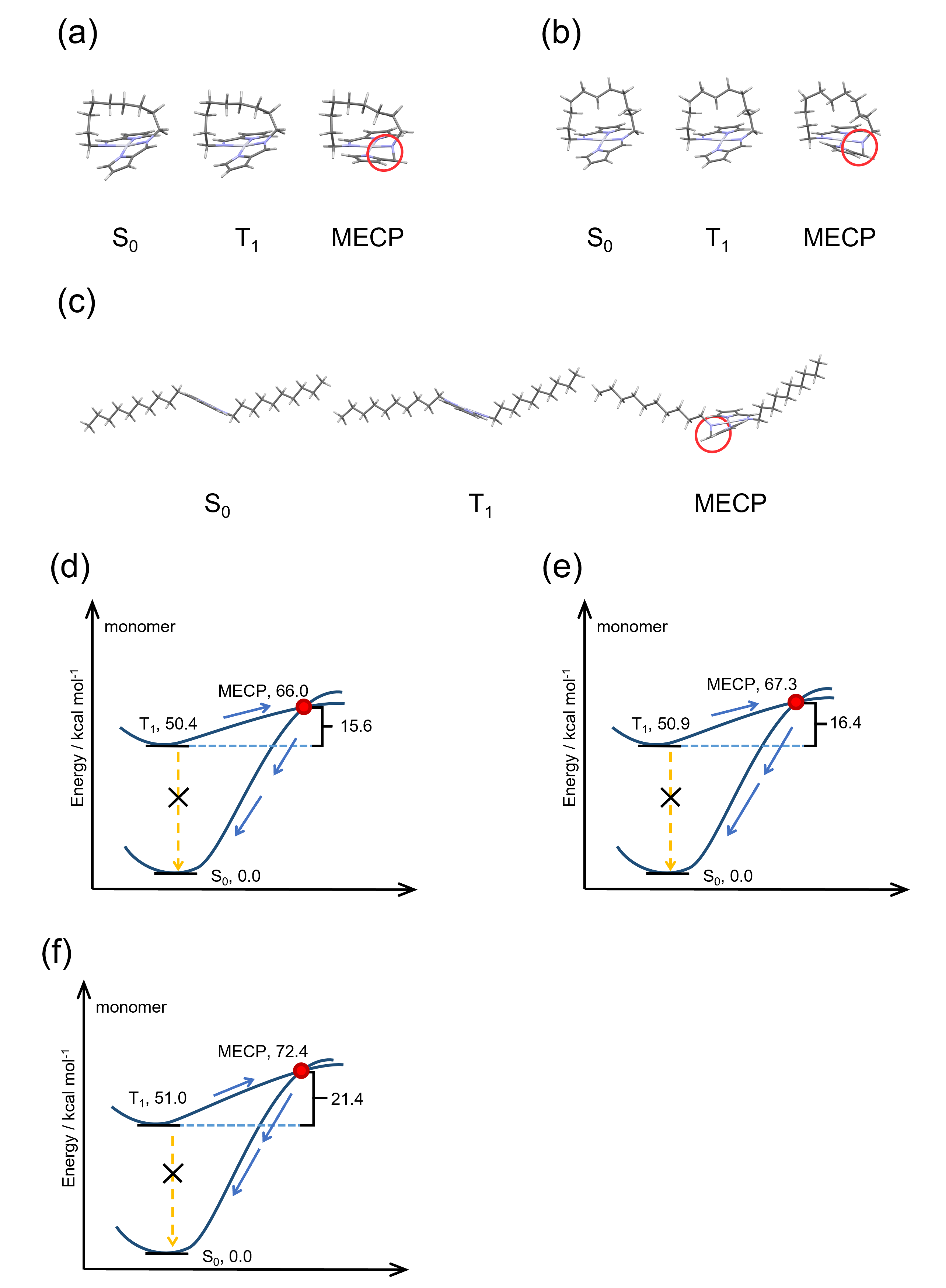
**

1. DFT-calculated molecular structures and energy diagrams of S₀, T₁ and MECP states for complexes **1a**, **1c** and **2**. (a–c) Optimized molecular structures of **1a**, **1c**, and **2** in the monomer state; (d–f) Energy diagrams of the S₀, T₁, and MECP states for each complex.

**
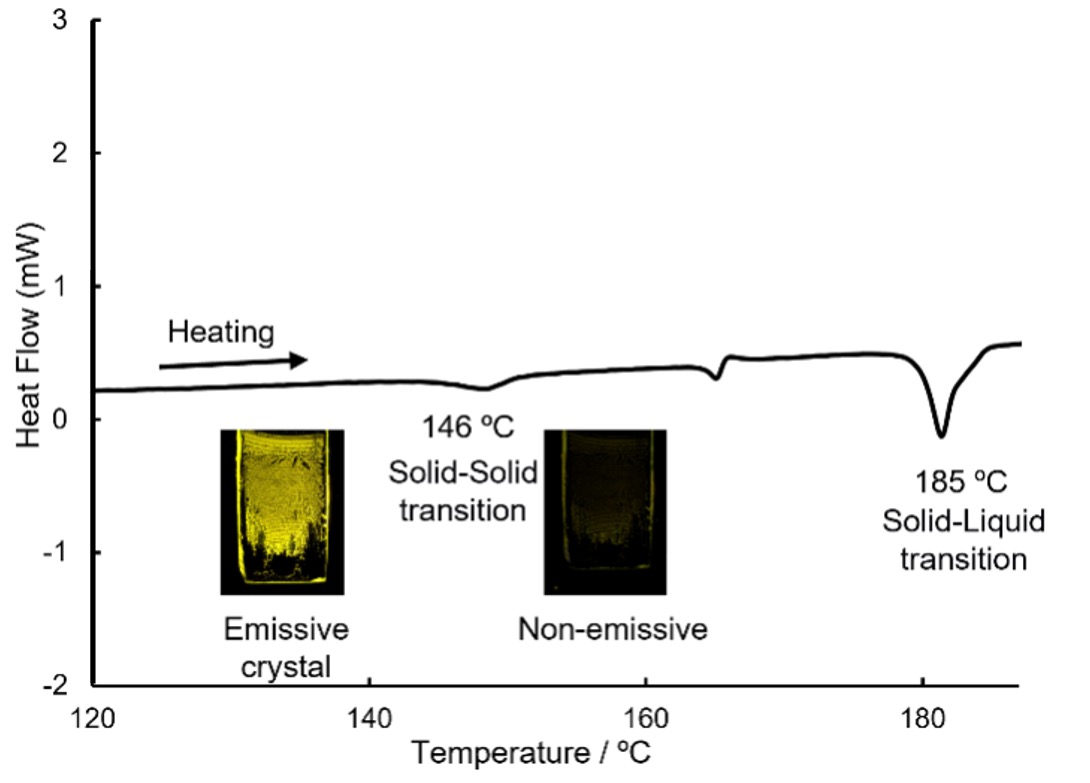
**

1. Differential scanning calorimetry diagram of emissive *rac*-**1d** crystal (0.95 mg) showing the solid–solid transition with a scan rate of 5 °C/min.


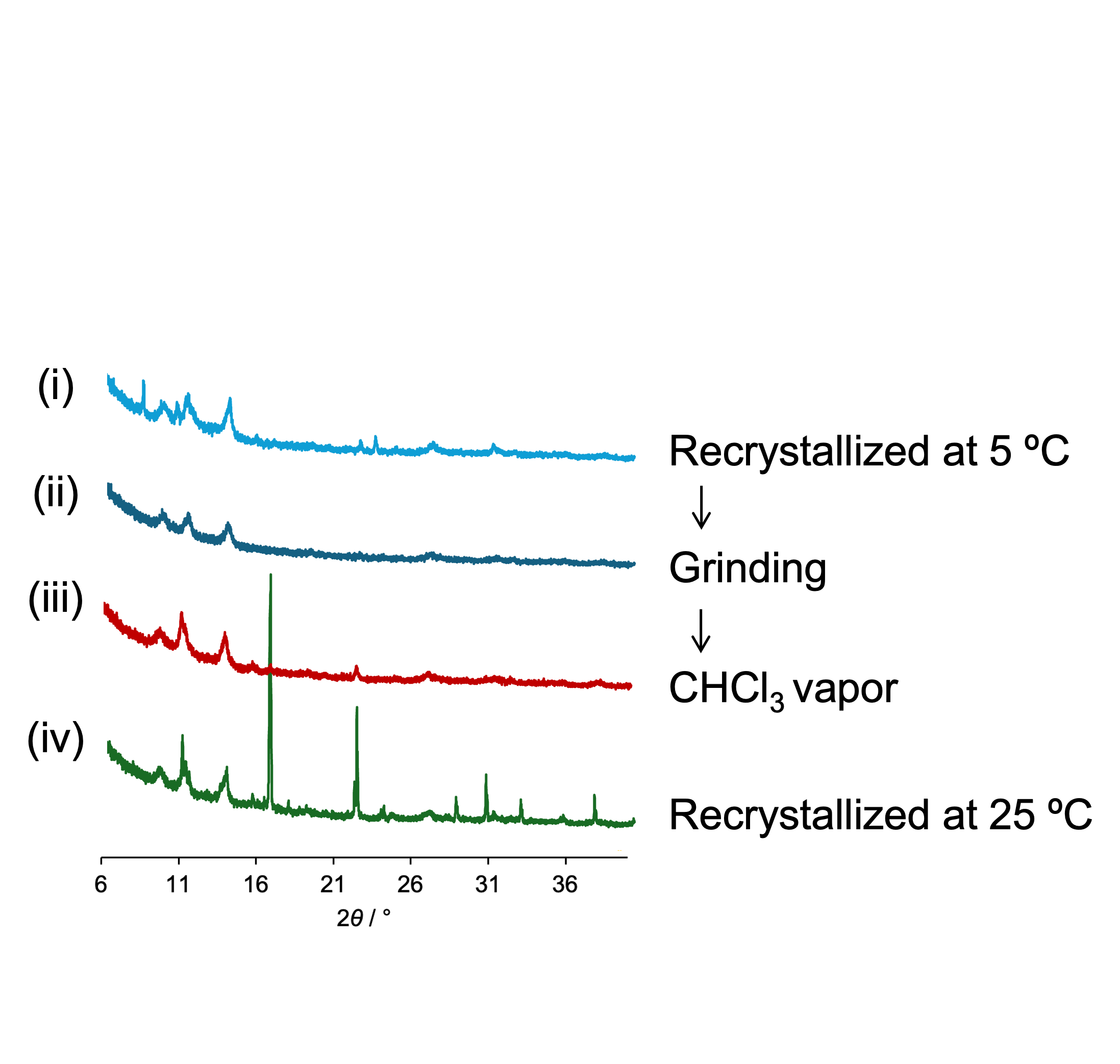


1. Powder X-ray diffraction (PXRD) patterns of *rac*-**1d** under different conditions. From top to bottom: (i) a crystalline sample obtained by recrystallization from acetonitrile at 5 °C (emissive crystal), (ii) the same sample after being ground (non-emissive), (iii) the ground sample after exposure to chloroform vapor (non-emissive), and (iv) a crystalline sample obtained by recrystallization from acetonitrile at 25 °C.


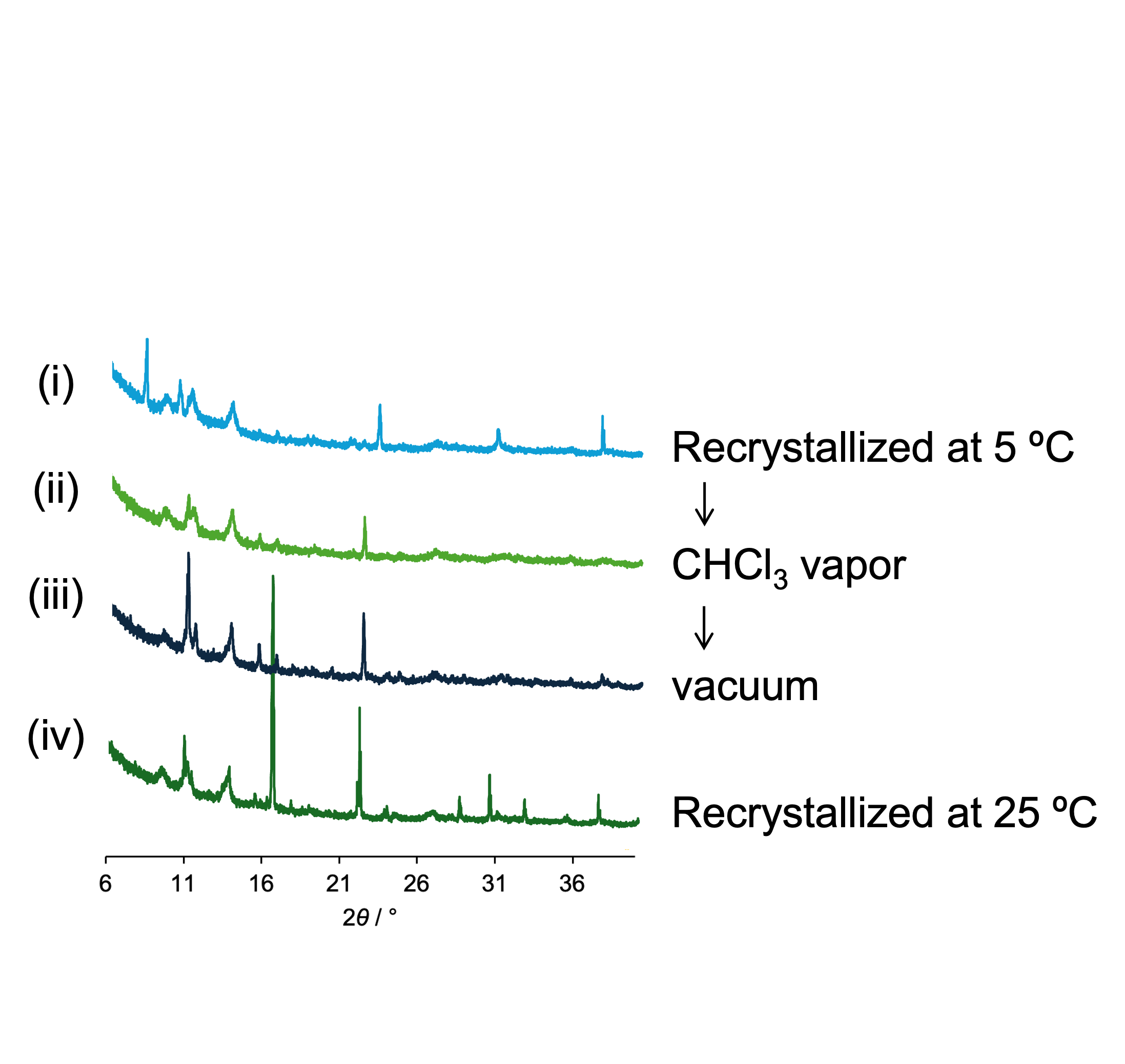


1. Powder X-ray diffraction (PXRD) patterns of *rac*-**1d** under different conditions. From top to bottom: (i) a crystalline sample obtained by recrystallization from acetonitrile at 5 °C (emissive crystal), (ii) the same sample after exposure to chloroform vapor (non-emissive), (iii) the chloroform-exposed sample after vacuum drying (non-emissive), (iv) a crystalline sample obtained by recrystallization from acetonitrile at 25 °C.


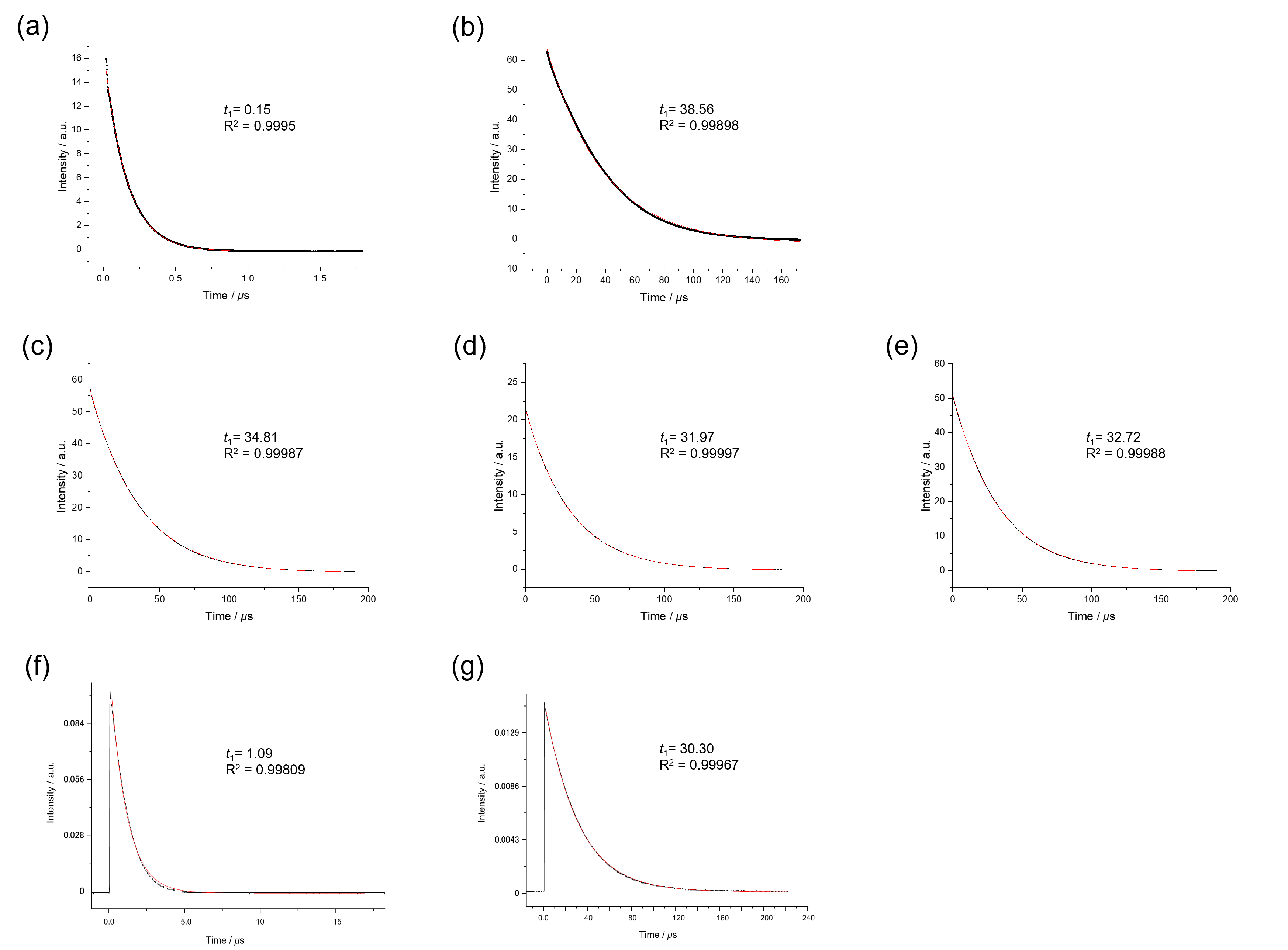


1. Emission decay curves (black lines, *λ*_ex_ = 355 nm) for *rac*-**1a**–**d** and **2** in 2-MeTHF (1.0 × 10⁻⁴ M). (a, b) *rac*-**1d** at 298 K and 77 K, (c–e) *rac*-**1a**–**1c** at 77 K and (f, g) **2** at 298 K and 77 K, respectively. Detection wavelengths: 540 nm for (a); 530 nm for (b–e); 550 nm for (f, g). Red lines indicate single-exponential fitting curves. Each decay curve was recorded with 6000 time points (horizontal axis) and 10,000 intensity units (vertical axis), averaged over 100 measurements to achieve a high signal-to-noise ratio. The data are plotted on a linear scale.


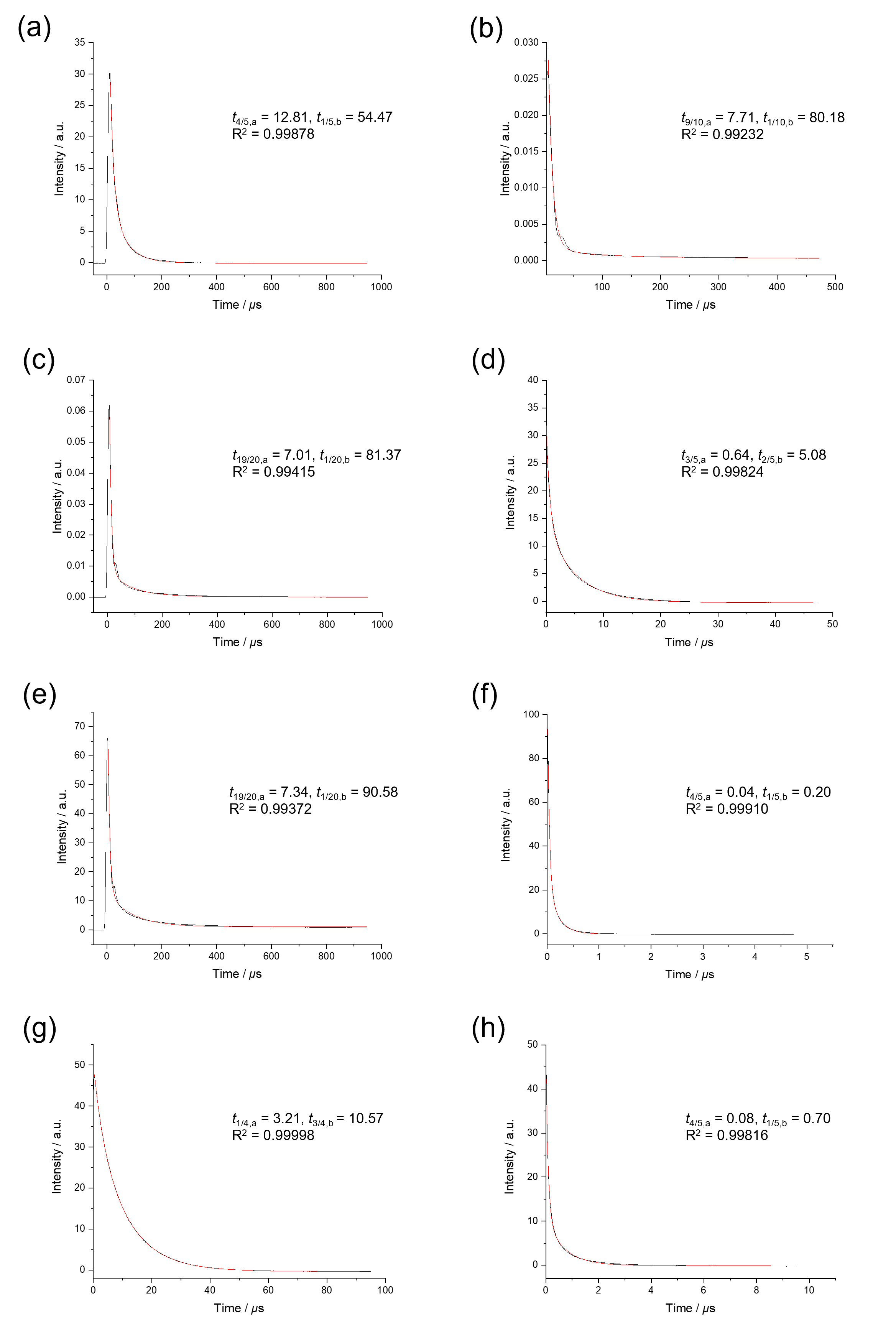


1. Emission decay curves (black lines, *λ*_ex_ = 355 nm) for crystals of *rac*-**1a–1d** obtained from acetonitrile under the following conditions: (a–d) *rac*-**1a–1d** recrystallized at 5° C (278 K) and measured at 25 °C (298 K); (e, f) *rac*-**1b** and **1d** recrystallized at 25 °C (298 K) and measured at 25 °C (298 K); (g, h) *rac*-**1d** recrystallized at 5 or 25 °C (278 or 298 K) and measured at –196 ℃ (77 K), respectively. Detection wavelengths: 570 nm for (a); 560 nm for (b–e, g); and 540 nm for (f, h). Red lines indicate single-exponential fitting curves. Each decay curve was recorded with 6000 time points (horizontal axis) and 10,000 intensity units (vertical axis), averaged over 100 measurements to achieve a high signal-to-noise ratio. The data are plotted on a linear scale.
2. Crystallographic data for *rac*-**1a**–**1d** recrystallized from acetonitrile at 5 °C (278 K). Data were collected at –160 ℃ (113 K).

|  | *rac*-1a | *rac*-1b | *rac*-1c | *rac*-1d |
| --- | --- | --- | --- | --- |
| formula | C_19_H_26_N_4_Pt | C_20_H_28_N_4_Pt・ CH_3_CN | C_21_H_30_N_4_Pt・ 0.5 CH_3_CN | C_22_H_32_N_4_Pt |
| *M*_r_ | 505.53 | 560.61 | 554.11 | 547.60 |
| *T*/K | 113 | 113 | 113 | 113 |
| crystal color, habit | yellow, block | yellow, block | yellow, block | yellow, block |
| crystal size/mm | 0.15×0.10×0.05 | 0.11×0.10×0.10 | 0.08×0.05×0.02 | 0.05×0.02×0.02 |
| crystal system | monoclinic | monoclinic | triclinic | monoclinic |
| space group | *C*2/c | *P*2/n | *P*-1 | *I*2/m |
| *a*/Å | 31.1766 | 14.5138(12) | 13.3221(4) | 12.6922(14) |
| *b*/Å | 12.5171 | 9.0888(7) | 14.2616(5) | 9.1476(9) |
| *c*/Å | 40.5209 | 16.3641(14) | 25.3077(8) | 19.4154(12) |
| *α*/° | 90 | 90 | 74.9920(3) | 90 |
| *β*/° | 102.029 | 98.277(18) | 85.811(2) | 106.225(9) |
| *γ*/° | 90 | 90 | 68.5730(3) | 90 |
| *V*/Å^3^ | 15465.7 | 2136.2(3) | 4322.1(3) | 2164.4(4) |
| *Z* | 32 | 4 | 8 | 4 |
| *ρ*_calcd_/g•cm^–3^ | 1.737 | 1.743 | 1.703 | 1.680 |
| *μ* (Mo_Kα_)/cm^–1^ | 72.36 | 65.60 | 65.08 | 64.96 |
| *F*(000) | 7872 | 1104 | 2184 | 1080 |
| 2*θ*_max_/° | 58.9 | 55.0 | 59.1 | 49.7 |
| No. of reflns measd | 140663 | 19956 | 41493 | 7289 |
| No. of obsd reflns | 20102 | 4889 | 19811 | 2087 |
| No. variables | 721 | 254 | 885 | 241 |
| *R*_1_ (*I* > 2*σ*(*I*))^a^ | 0.1189 | 0.0151 | 0.0551 | 0.0901 |
| *wR*_2_ (all reflns)^b^ | 0.2846 | 0.0346 | 0.1242 | 0.2254 |
| Goodness of fit | 1.150 | 1.009 | 1.007 | 1.056 |

[a] *R*_1_ = Σ(|*F*_o_|–|*F*_c_|)/Σ(|*F*_o_|). [b] *wR*_2_ = [Σ[w(*F*_o_^2^–*F*_c_^2^)^2^]/Σw(*F*_o_^2^)^2^]^1/2^.

1. Crystallographic data for (*S*)-**1b**, (*R*)-**1c**, (*R*)-**1d** and **2** recrystallized from acetonitrile at 5 °C (278 K). Data were collected at –160 ℃ (113 K).

|  | (*S*)-1b | (*R*)-1c | (*R*)-1d | 2 |
| --- | --- | --- | --- | --- |
| formula | C_20_H_28_N_4_Pt | C_21_H_30_N_4_Pt | C_22_H_32_N_4_Pt | C_30_H_50_N_4_Pt |
| *M*_r_ | 519.55 | 533.58 | 547.60 | 661.83 |
| *T*/K | 113 | 113 | 113 | 113 |
| crystal color, habit | yellow, block | yellow, block | yellow, block | yellow, block |
| crystal size/mm | 0.03×0.02×0.01 | 0.08×0.05×0.02 | 0.15×0.10×0.10 | 0.04×0.02×0.01 |
| crystal system | monoclinic | orthorhombic | monoclinic | monoclinic |
| space group | *P*2_1_ | *P*2_1_2_1_2_1_ | *P*2_1_ | *P*2_1_/*c* |
| *a*/Å | 9.4846(2) | 9.5791(11) | 10.3722(2) | 20.1666(14) |
| *b*/Å | 10.1557(3) | 10.5491(14) | 16.9540(3) | 5.1504(3) |
| *c*/Å | 9.9659(3) | 19.9568(2) | 11.9744(2) | 14.0406(8) |
| *α*/° | 90 | 90 | 90 | 90 |
| *β*/° | 99.081(2) | 90 | 93.863(2) | 90.539(6) |
| *γ*/° | 90 | 90 | 90 | 90 |
| *V*/Å^3^ | 947.9(5) | 2016.7(4) | 2100.9(7) | 1458.3(16) |
| *Z* | 2 | 4 | 4 | 2 |
| *ρ*_calcd_/g•cm^–3^ | 1.820 | 1.757 | 1.731 | 1.507 |
| *μ* (Mo_Kα_)/cm^–1^ | 74.11 | 69.70 | 66.93 | 48.35 |
| *F*(000) | 508 | 1048 | 1080 | 672 |
| 2*θ*_max_/° | 58.8 | 58.5 | 58.4 | 49.4 |
| No. of reflns measd | 9594 | 38735 | 20565 | 13281 |
| No. of obsd reflns | 4244 | 5206 | 9543 | 3655 |
| No. variables | 226 | 235 | 487 | 161 |
| *R*_1_ (*I* > 2*σ*(*I*))^a^ | 0.0240 | 0.0135 | 0.0231 | 0.0496 |
| *wR*_2_ (all reflns)^b^ | 0.0561 | 0.0277 | 0.0483 | 0.0786 |
| Goodness of fit | 1.048 | 1.025 | 0.967 | 0.948 |

[a] *R*_1_ = Σ(|*F*_o_|–|*F*_c_|)/Σ(|*F*_o_|). [b] *wR*_2_ = [Σ[w(*F*_o_^2^–*F*_c_^2^)^2^]/Σw(*F*_o_^2^)^2^]^1/2^.

1. Crystallographic data for *rac*-**1a** recrystallized from acetonitrile at 5 °C (278 K) and 60 °C (333K). Data were collected at –160 ℃ (113 K).

|  | *rac*-1a from 25 °C | *rac*-1a from 60 °C |
| --- | --- | --- |
| formula | C_22_H_32_N_4_Pt | C_22_H_32_N_4_Pt |
| *M*_r_ | 547.60 | 547.61 |
| *T*/K | 113 | 113 |
| crystal color, habit | yellow, block | orange, block |
| crystal size/mm | 0.04×0.01×0.01 | 0.10×0.05×0.05 |
| crystal system | monoclinic | monoclinic |
| space group | *P*2_1_/*n* | *P*2_1_/*n* |
| *a*/Å | 10.4527(2) | 10.4268(2) |
| *b*/Å | 17.0981(4) | 17.0322(3) |
| *c*/Å | 11.9661(2) | 11.9996(2) |
| *α*/° | 90 | 90 |
| *β*/° | 97.584(2) | 97.035(2) |
| *γ*/° | 90 | 90 |
| *V*/Å^3^ | 2119.9(7) | 2115.0(7) |
| *Z* | 4 | 4 |
| *ρ*_calcd_/g•cm^–3^ | 1.716 | 1.720 |
| *μ* (Mo_Kα_)/cm^–1^ | 66.06 | 120.30 |
| *F*(000) | 1080 | 1080 |
| 2*θ*_max_/° | 59.1 | 152.1 |
| No. of reflns measd | 22531 | 13862 |
| No. of obsd reflns | 5388 | 4120 |
| No. variables | 244 | 244 |
| *R*_1_ (*I* > 2*σ*(*I*))^a^ | 0.0401 | 0.0512 |
| *wR*_2_ (all reflns)^b^ | 0.1221 | 0.1443 |
| Goodness of fit | 1.136 | 1.045 |

[a] *R*_1_ = Σ(|*F*_o_|–|*F*_c_|)/Σ(|*F*_o_|). [b] *wR*_2_ = [Σ[w(*F*_o_^2^–*F*_c_^2^)^2^]/Σw(*F*_o_^2^)^2^]^1/2^.

**References**

[18] F. Neese, F. Wennmohs, U. Becker, C. Riplinger, *J. Chem. Phys.* **2020**, *152*, 224108.

[19] F. P. Fanizzi, F. P.; Intini, L. Maresca G. Natile, *J. Chem. Soc., Dalton Trans.* **1990**, 199–202.
